# Exploring self-experience practices in dementia care: A scoping review

**DOI:** 10.1371/journal.pone.0302929

**Published:** 2024-05-07

**Authors:** Janina Wittmann, Anja Bieber, Joanne Carroll, Kealan Forristal, Louise Hopper, Niels Janssen, Gabriele Meyer, Marianna Riello, Marjolein de Vugt, Dorothee Bauernschmidt

**Affiliations:** 1 Institute of Health and Nursing Science, University Medicine Halle, Martin Luther University Halle-Wittenberg, Halle (Saale), Germany; 2 School of Psychology, Dublin City University, Dublin, Ireland; 3 Department of Psychiatry and Neuropsychology, Alzheimer Centre Limburg, School for Mental Health and Neuroscience, Maastricht University, Maastricht, The Netherlands; 4 SPES Group, Trento, Italy; Erasmus University Rotterdam, NETHERLANDS

## Abstract

**Background:**

Recognised as essential for high-quality dementia service, person-centred care aims to understand and respect the unique needs of each individual. Self-experience practices may offer caregivers an opportunity to acquire knowledge, empathy, and skills related to person-centred care, especially through recreating experiences similar to dementia. Given the need to enhance the understanding of self-experience practices in dementia care, a more comprehensive investigation of these training interventions for (future) caregivers is needed.

**Methods:**

We conducted a scoping review to map the evidence on the use of self-experience practices in dementia training. We systematically searched Cochrane Library, MEDLINE via PubMed, CINAHL, and Web of Science. We also searched for grey literature, as well as registry entries, and conducted backward citation tracking of included reviews. We analysed data on intervention characteristics, factors influencing the implementation, and learning outcomes based on Kirkpatrick’s model.

**Results:**

We included 44 reports across 30 intervention programmes. The majority of reports (91%) were published from 2016 onwards, with 32% originating from the USA and 25% from the UK. We identified passive, interactive, immersive, and multicomponent self-experience interventions in dementia education and training. Learning outcomes based on Kirkpatrick’s model were fairly distributed across all identified modalities. Both consumers and providers emphasised aspects related to the development and implementation of practices, particularly organisational-related considerations such as temporal and spatial planning of trainings.

**Conclusions:**

Our review highlights diverse interventions incorporating self-experience practices, with an increasing role for technological tools. While self-experience interventions engage participants, the impact on individuals with dementia and organisational levels remain largely unreported. Our overview, informed by current literature, underscores unique considerations and challenges associated with dementia-related self-experience practices. Implementing and evaluating complex training interventions using self-experience practices should consider ethical aspects.

**Trial registration:**

**Registry:** Registered within the Open Science Framework (available at https://osf.io/fycxa/).

## Introduction

A central aspect of tailored care for individuals with dementia lies in understanding how to provide appropriate and individualised support. Acknowledging the individuality and uniqueness of a person with dementia is essential [1] to develop suitable care strategies that address their needs and preserve personhood and dignity. Realising the perspectives and experiences of individuals with dementia is crucial [1], considering the diverse, varied and complex nature of their lived experiences [2]. In addition to understanding the condition itself, genuine empathy for individuals with dementia plays a crucial role in truly grasping the reality of their circumstances. By integrating knowledge of dementia and empathy into care, a more holistic approach can emerge [3].

Person-centred care represents an individualised, values-based approach that aims to understand the needs of the person with dementia, and subsequently strengthen their personhood [4]. Due to the often dynamic and progressive nature of dementia, person-centred practice is particularly relevant in terms of the core values it encompasses, such as “respect for personhood”, “sharing autonomy”, and “demonstrating mutual respect and understanding” [5]. As an approach that acknowledges the importance of individual needs and preferences, person-centred care enables people with dementia to actively participate in decisions [4,5].

Person-centredness places the focus on individuals as a whole, shifting the focus from mere “patient care” to encompass the broader context of the “person”. Related concepts such as “individualism” or “value-based care” have similarities with person-centred care in terms of shared values and objectives but are less expansive in their scope [5]. Person-centred care also recognises the indispensable role of caregivers. The dyadic relationship between caregivers and individuals with dementia is pivotal, as caregivers play a crucial role in providing tailored support and maintaining the personhood of individuals with dementia [4,5].

Person-centred care may lead to positive outcomes for people with dementia, including a reduced risk of behavioural problems [6], neuropsychiatric symptoms [7,8], and improved quality of life [7–9]. Person-centred care can have a positive impact on the caregivers themselves, influencing staff behaviour, and job satisfaction of professional caregivers, as well as family satisfaction of informal caregivers [10–13]. As a result, this may enhance further adoption of person-centred care on the organisational level. Further research indicates that person-centred care is associated with a decrease in length of hospital stays, lower hospitalisation rates, and fewer diagnostic procedures [14].

Although person-centred care has an impact on the individual level (persons with dementia, dyads), on the meso level (institutions, organisations, communities), and on the macro level (health services system), and is a recognised international priority [15], there are issues with its integration into practice [16–18]. This gap may be ascribed to potential reservations in the staff attitudes towards person-centred care [16,17], insufficient resources [16–18], or inadequate education of both professional and informal caregivers [16].

The complexity of interventions aimed at promoting person-centred care approaches pose a particular challenge for implementation, as they require rigorous methods to be successfully implemented in the long term [19,20]. Already in 2014 [21] a so called “implementation error” was postulated in the context of dementia-specific interventions, which is often overlooked in evaluation methods. This error can be observed during pragmatic trials of effectiveness and thus propose a paradigm shift in the methodology for psychosocial research. Among others, it emphasises not only the need to assess the effectiveness of interventions but also to ensure their successful implementation, respectively, their implementation effectiveness.

Training programmes that enable formal and informal caregivers to acquire knowledge, empathy, and care skills are considered crucial for treating individuals with dementia adequately and effectively taking on the caregiver role [15]. One way to achieve this is through training approaches that try to convey what is it like to live with dementia and see the world from the perspective of a person with dementia. Learning through self-experience, also known as experiential learning, has been defined as “the process whereby knowledge is created through the transformation of experience” [22]. Self-experience practices as experiential learning methods offer participants a tangible experience from which they can gain their own knowledge, derive actions, and cultivate empathy [22]. For instance, in self-experience interventions, caregivers may engage in role-play exercises or virtual simulations to gain insight into the physical, cognitive, and emotional challenges faced by individuals with dementia. Self-experience training sessions may involve activities including guided reflection, group discussions, and interactive workshops.

In recent years, self-experience training has been increasingly utilised in healthcare settings, using various methods (e.g., virtual simulation, theatre) with different modes of action [23]. Compared to more conventional lectures or didactic teaching strategies, self-experience practices in healthcare education and training have proven beneficial in achieving learning goals. The immersive nature of these training interventions means that outcomes are often driven by participants’ own motivation, personal feelings, and attitudes towards the experience [23].

So far, studies have described self-experience practices as beneficial for training caregivers of people with dementia to promote empathy for and understanding of (the person with) dementia [24,25]. However, existing recommendations for dementia training [26,27] mainly focus on general training methods (without specific focus on self-experience practices) related to dementia, with few addressing experiential learning methods, thus revealing knowledge gaps in this type of training. The implementation of training interventions, in general, is an essential aspect, which may impact the effectiveness of experiential learning methods [27].

A systematic and comprehensive overview on self-experience training programmes in dementia care is currently lacking. Therefore, our objective was to systematically identify, map, and compile the breadth of evidence available on self-experience practices in dementia care. Furthermore, this study aims to identify what helps (i.e., facilitating factors) and what hinders (i.e., barriers) the implementation of these types of training practices to improve our understanding of how self-experience training should be implemented in practice. The learning outcomes of these interventions will also be determined. The following questions guided our research:

a) What self-experience practices are reported to help gain a deeper understanding of the experiences of individuals with dementia in the international literature?
b) What are the potential barriers and facilitators in the development and implementation of self-experience practices in dementia care?
c) What level(s) of outcomes are addressed in the evaluation of self-experience practices?

This study is part of the transnational (Germany, Italy, Ireland, the Netherlands) Erasmus+ funded research project INTenSE (Improving demeNtia care Through Self-Experience [28]). The primary goal of INTenSE is to develop an innovative self-experience toolkit that can educate, equip, and train health and social care professionals to provide the best possible care for people living with dementia. By employing innovative learning methods, participants can gain an understanding of the lived experience of individuals with dementia. To develop the INTenSE training platform, it was essential to obtain a comprehensive overview of existing self-experience practices and their application in the context of dementia training and education.

## Methods

A scoping review was conducted to map the available evidence on the use of self-experience practices in dementia education and training. We registered our review in the Open Science Framework (available at https://osf.io/fycxa/). The initial search was first conducted from January to February 2021 and updated between November 2022 to February 2023. The review followed the methodological approach of Arksey and O’Malley [29], along with the extension proposed by Levac et al. [30] and the updated guidance from the Joanna Briggs Institute (JBI) by Peters et al. [31]. We critically appraised the included studies by means of the Mixed Methods Appraisal Tool (MMAT; [32]) in order to determine the quality of the evidence and the implications of the evidence for practice. We adhered to the “Preferred Reporting Items for Systematic Reviews” Statement for Scoping Reviews (PRISMA-ScR [33]; checklist provided in S1 File).

### Eligibility criteria

We included studies evaluating self-experience practices in the context of dementia, irrespective of their design and type of reporting. Reports in English, German, Dutch or Italian published from 2010 onwards were included. The timeframe was chosen based on an initial exploratory search to determine the temporal scope for the final search.

Reports on self-experience interventions for informal and/or professional caregivers (including those in training) of people with dementia were included. Passive interventions, where participants were initially not actively involved (e.g., by watching a theatrical performance), were considered as long as the intervention included a discussion or reflection in which the caregivers actively participated. Additionally, multicomponent training programmes that included self-experience practices as one component were included.

Exclusion criteria were as follows: 1) interventions specifically targeting people with dementia as participants and 2) reports describing generic self-experience practices or those related to other conditions (e.g., Parkinson’s disease, depression).

### Information sources, search strategy and selection of sources

To ensure coverage of a wide range of self-experience practices, the search strategy comprised seven components. Each component included synonyms, descriptions and database-specific vocabulary combined with respective permutations. To specify our comprehensive search approach, we limited the search to the title and abstract field. The search strategies are provided in the S1 File.

We searched Cochrane Library, MEDLINE via PubMed, CINAHL, and Web of Science in November 2022. Research protocols were included to take into account upcoming or ongoing research projects. In addition, relevant information from the protocols that exceeded the content of publications related to the included studies was considered during data extraction. All authors searched for grey literature to identify reports from leading national and international professional societies and health organisations (e.g., health departments, research institutes) in the respective countries. Furthermore, in January and February 2023, the International Clinical Trials Registry Platform (ICTRP) and the International prospective register of systematic reviews (PROSPERO) were searched to identify ongoing studies. This helped to obtain a comprehensive picture of research activities and to gain insights into potentially emerging self-experience practices, encompassing published works, ongoing studies, and reports in grey literature. The search strategy was complemented by backward citation tracking of the included reviews.

A stepwise screening process was performed using the Rayyan web app, which supports the two-stage blinded screening process [34]. Two researchers (JW and DB) independently screened titles and abstracts; then assessed full texts for eligibility. Discrepancies were discussed until consensus was reached.

### Charting the data and data items

To provide a comprehensive overview of the interventions and their components, an extraction sheet was developed using criteria of the Template for Intervention Description and Replication (TIDierR) checklist [35] and the revised Criteria for Reporting the Development and Evaluation of Complex Interventions (CReDECI 2) guideline [36]. In addition, study characteristics (aim, design, country, population, etc.), barriers and facilitators in the development and implementation of practices as well as data on learning outcomes based on Kirkpatrick’s model [37,38] were extracted. The model encompasses the learner’s immediate reaction to the training (level 1), the acquisition of knowledge, skills, and attitude through the training (level 2), the resulting skills and behaviour (level 3), and the ultimate outcomes and practice results (level 4).

Data extraction of a subsample of 10% of the reports was performed independently by two reviewers (JW and DB) to enhance inter-rater agreement. Extracted data of remaining reports were cross-checked for accuracy. For reports in Dutch and Italian, the respective national teams handled the data extraction and provided the data in English. We ensured homogeneity and consistency of data collection and extraction across languages through various mechanisms. These included adopting a jointly agreed study protocol, approving of a collective data extraction tool, and conducting consultations with respective countries via video conferences to address any queries regarding the content and process of data extraction.

### Critical appraisal

Critical appraisal in scoping reviews is not mandatory; however, we appraised the literature using the Mixed Methods Appraisal Tool (MMAT; [32]). Two authors (JW and DB) assessed the reported methodological quality of the reports to identify any potential source of systematic bias. This included an independent assessment of 10% of the reports. Any discrepancies in the assessment were resolved through discussion and consensus finding. Furthermore, reports in Dutch and Italian language were appraised by the respective research teams. The critical appraisal of all included reports can be found in S1 File.

### Synthesis of results

All the analyses were conducted by JW, validated by DB, and subsequently discussed within the research team (GM and AB). We aimed (a) to develop an understanding of the characteristics of the included reports and the range of self-experience interventions reported. In addition, (b) factors reported in the included publications that influenced the development and implementation of self-experience practices were categorised based on the type of intervention and the perspective of reporting (provider or consumer; [39]), and classified as either having a positive (facilitator) or negative (barrier) effect. A tabular overview of barriers and facilitators per intervention type can be found in S1 File. Another aim was (c) to gain insights into the intended and reported learning outcomes. These were categorised into one or several of the four levels defined within Kirkpatrick’s model [37,38]. Considering that empathy includes both an emotional and a cognitive dimension [40], we assumed that empathy can influence both the participants’ reaction and their learning. Therefore, in cases where empathy was addressed and examined as a learning outcome, we considered both levels (1 and 2). The results of the analysis are presented in narrative, tabular, and graphical form.

## Results

**Fig 1:**
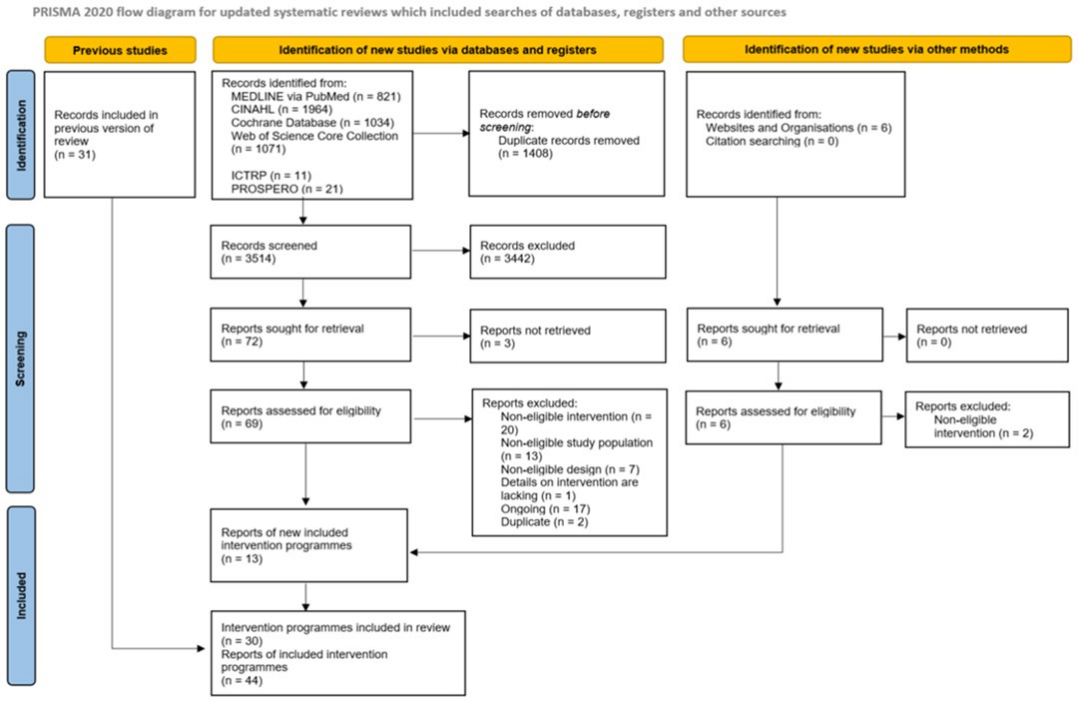
PRISMA flow diagram of study selection.

Our search yielded a total of 4928 references. In the backward citation tracking of the included reviews, we did not identify any additional references. A total of 44 reports on 30 intervention programmes were included in our review (Fig 1).

### Characteristics of sources of evidence

The majority (n = 40; 91%) of reports were published from 2016 onwards; only four sources were published before this period ([41–44]; see S1 File).

Fourteen studies (32%; [44–57]) were conducted in the USA, eleven (25%; [58–68]) in the UK, five in the Netherlands (11%; [42,69–72]), four in South Korea (9%; [73–76]), three in Australia (7%; [77–79]), two in Canada (5%; [41,43]), and one report (2%) in Ireland [80], Norway [81], Lithuania [82], China [83], and Taiwan [84], respectively.

The type of study design was evenly distributed across the included publications. Twelve publications [41,43,51,52,56,60,65–67,73,74,77] followed a qualitative approach and mostly focused on describing participants’ experiences and perspectives on the (impact of the) intervention. Fewer publications were aimed at developing a new intervention with qualitative methods. Eighteen studies reported a quantitative design [44–47,49,53,54,68,70–72,75,76,78,79,82–84], including four (Cluster-) RCTs [44,75,79,84]. These studies primarily focused on evaluating the clinical effectiveness of a (new) intervention by assessing different outcomes related to caregivers. Most RCTs evaluated self-experience practices in comparison to conventional education methods (e.g., lectures, e-book modules). Only one RCT [75] evaluated the self-experience training programme, including an evaluation of participant satisfaction with the training. The remaining publications (n = 12; [42,48,50,55,57–59,61,62,64,80,81]) applied qualitative and quantitative research methods, partly with the integration of both data strands in terms of a mixed-methods design. These studies mostly focused on the development of an intervention prototype or served as a pilot study. Additionally, two study protocols of included studies were identified [63,69]. Few publications of grey literature [61,66,76,80] were identified. The search for study registrations did not provide insights into any emerging self-experience practices or any additional relevant information.

### Modalities and types of intervention programmes

Table 1 provides an overview of the intervention programmes. We distinguished between the modality and the type of intervention.

**Table 1:** Modality and type of intervention programmes.

Modality of intervention programme	Type of intervention programme	Name of intervention programme^a^	Reports included
Passive interventions	Film interventions	Barbara’s Story & Barbara’s Evolving Story	[60]
		The Alzheimer Experience (AlzExp)	[71]
	Theatre interventions	I’m Still Here	[41]
Interactive interventions	Physical simulation interventions	Dementia Live^™^ (DL) *(includes nationally adapted version*: *Korean dementia simulation program)*	[73–76]
		De Abreu 2017*	[47]
		Virtual Dementia Tour^®^ (VDT) *(includes adapted version*: *Physical Dementia Tour (PDT))*	[46,48,52,56,57,66–68,80]
	Role-play interventions	Haugland 2018*	[81]
		Maharaj 2015*	[44]
Immersive interventions	Game interventions	IDO serious game	[82]
	Virtual Reality interventions	A walk through dementia (AWTD)	[61]
		Beatriz Lab	[45]
		Educational Dementia Immersive Experience (EDIE)	[79]
		myShoes	[58]
		Virtual Reality Dementia Tour (VRDT)	[57]
	Mixed Reality interventions	Into D’mentia	[42,69,70]
Multicomponent interventions	Combination of self-experience practices	Argyle 2016*	[59]
		Kontos 2010*	[43]
		Leah 2017*	[65]
		Peng 2020*	[83]
	Combination of self-experience practices with other learning methods	Lorio 2016*	[53]
		Tier 2 Programme	[62]
		Dementia Education and Learning Through Simulation 2 (DEALTS 2)	[63,64,73]
		Alzheimer’s Australia Vic Virtual Dementia Experience^™^	[77,78]
		Kimzey 2021*	[51]
		Sung 2022*	[84]
		Kimzey 2018*	[49]
		Kimzey 2020*	[50]
		Dementia Care Boot Camp	[54,55]
		Through the D’mentia Lens (TDL)	[72]
		Han 2020*	[75]

Note:

^a^ When no name was reported, the name of the first author and the year of publication were used to designate the intervention.

*Abbreviation*: IDO serious game = Innovative Digital Training Opportunities on Dementia for Direct Care Workers (IDO) serious game.

In our case, modality refers to the methodical delivery of an intervention programme and how the presentation of the content facilitated interaction with the participants. This includes both active and passive roles that participants may assume. We were able to distinguish between four modalities, defined as passive, interactive, immersive, and multicomponent interventions.

In passive interventions [41,60,71], for example theatre interventions, the participants’ experiences occur through reflective observation. Participants are exposed to a concrete scenario in which they are not actively involved. Afterwards, participants discuss their observations and experiences and reflect on the appropriateness of the caregivers’ behaviour in the simulated care environment. Interactive interventions [44,46–48,52,56,57,66–68,73–76,80,81] are non-virtual simulation activities in which participants are actively engaged by using materials (e.g., goggles, headphones) that can physically alter their perception. This modality is often supplemented by reflections or discussions, which usually serve to reflect upon their experience and support individual learning processes. Immersive interventions [42,45,57,58,61,69,70,79,82] offer the possibility to introduce participants to a simulated environment, for example through Virtual Reality (VR), and to allow active immersion and interaction with this environment with the help of technological tools. Multicomponent interventions [43,49–51,53–55,59,62–65,72,75,77,78,83,84] refer to the use of educational components to deliver the programme (e.g., lectures combined with VR).

We defined the type of intervention in our study as the nature of the intervention programme, encompassing factors such as use of technology and the incorporation of multiple components. We distinguish between nine different types of self-experience practices: film, theatre, physical simulation, role-play, game, Virtual Reality, Mixed Reality interventions, a combination of self-experience practices, and a combination of self-experience practices with other learning methods.

Table 2 provides an overview of the characteristics of the intervention programmes. The target groups of intervention programmes varied. In addition to informal caregivers, the target audience also included healthcare professionals (such as nurses, therapists, or physicians) as well as students (such as nursing or medical students). If specified, the duration of the training ranged from 30 minutes to 2 days.

**Table 2:** Characteristics of intervention programmes included.

Intervention programme^a^ (References)	Objective(s)	Setting^b^	Population	Material^c^	Duration (total)/ Duration (SE component)/ Frequency
Barbara’s Story & Barbara’s Evolving Story [60]	Improving dementia awareness; Emotional engagement with the experience of people with dementia	National health service system “Trust”	Healthcare professionals; Other professionals (non-clinical staff)	Film ‘Barbara’s Story’; Additional films ‘Barbara’s evolving story’; Resource pack	N/A N/A Once
AlzExp [71]	Improving public awareness and enhancing knowledge and understanding about the experiences and needs of people with dementia	Online (dissemination via the Dutch Alzheimer’s Society)	Informal caregivers; Healthcare professionals	Film; Website	N/A N/A Once
I’m Still Here [41]	Improving understanding about the experiences of people with dementia	Public	Informal caregivers; Healthcare professionals; Students	Drama ‘I’m still here’	N/A N/A Once
Dementia Live^™^ [73–76]	Providing a realistic simulation of life with dementia	N/A	Informal caregivers; Healthcare professionals	Headphones with MP3 players; Eyewear; Gloves	30–40 min N/A Once
De Abreu 2017* [47]	Increasing awareness of and attentiveness to the physical and cognitive changes experienced by a person with dementia	N/A	Students	Headphones; Goggles; Gloves	N/A 10 min Once
VDT^®^ [46,48,52,56,57,66–68,80]	Communicating the physical and mental challenges of people with dementia to better understand what it is like to live with this condition	University; Healthcare facility	Informal caregivers; Students; Healthcare professionals; Other professionals (such as managers, lecturers); Voluntary groups; General public	Goggles; Headphones; Gloves; Shoe inserts; Lighting; (Partly: additional materials for tasks)	Max. 2 hours 8–10 min Once
Haugland 2018* [81]	Gaining enhanced competence regarding issues such as communication, cooperation, legislation, documentation and attitudes	University	Students	Simulation information for participants	N/A Approx. 15 min (per scenario) 2 sessions
Maharaj 2015* [44]	Changing students’ attitudes and improving knowledge in the context of dementia	University	Students	Setup of the room as a medical unit (hospital beds, monitors, etc.); Preparation of the participants (make-up, wigs etc.); Simulation information for participants	N/A N/A Once
IDO serious game [82]	Improving knowledge and ability to apply care concepts in real life situations; Improving behavioral skills, attitudes, motivation, and commitment to dementia care; Increasing general job satisfaction	Online (access via website)	Social care professionals; Informal caregivers; People with early signs of dementia	Website	N/A N/A Once
Into D’mentia [42,69,70]	Increasing understanding of and empathy for people with dementia	N/A	Informal caregivers; Healthcare professionals	Into D’mentia-Simulator; Speaker vest with microphones	40 min 20 min Once
AWTD [61]	Increasing understanding of the lived experience of dementia; Influencing patient care in a humane way	N/A	Healthcare professionals; Students	Smartphone-App	N/A N/A N/A
Beatriz Lab [45]	Generating understanding and empathy for people with dementia and their families	University	Students	Smartphone-App; VR goggles; Gaming laptops	N/A Max. 30 min Once
EDIE [79]	Increasing empathy and understanding of the dementia care environment among dementia care workers: Improving knowledge and attitudes towards dementia	Public (access via Dementia Australia Centre)	Healthcare professionals	Smartphone-App; VR goggles; Headset	Approx. 3 hours N/A Once
myShoes [58]	Increasing awareness of the symptoms and lived experiences of people with dementia; Supporting an increase in empathy; Encouraging participants to reflect on their practice	N/A	Students	VR device (head-mounted display, goggles); Mouse; Keyboard	N/A 15 min Once
Virtual Reality Dementia Tour [57]	Building empathy among caregivers	N/A	Students	VR device (head-mounted display, goggles); Headphones; Remote hand controls	N/A N/A Once
Argyle 2016* [59]	Improving insight, awareness, empathy and communication towards people with dementia and their families; Demonstrating relevant skills in person-centred care	University	Healthcare professionals	N/A	1 day 3 hours 15 min Once
Kontos 2010* [43]	Introduction to person-centred care; Promoting understanding of people with dementia; Promoting critical reflection on one’s own practice; Improving quality in practice	N/A	Healthcare professionals	DVDs with five vignettes; Scripts for role-play	2 hours/session N/A 12 weeks^d^
Leah 2017* [65]	Gaining a greater understanding of person-centred care for people with dementia	Hospital	Healthcare professionals	Episode 3 of ‚Barbara’s Story‘; Glasses; Gloves; Popcorn; Paper tape; Medicine bottles with child-safe lids; Different types of medication; Dosette boxes	2 days N/A Once
Peng 2020* [83]	Increasing empathy for people with dementia	University	Students	Movie ‚Still Alice‘; Slippers; Goggles; Gloves; Tape Furnished room; Audiotapes of radio noise; Socks; Towels; Teeth brush; Small balls	N/A 8 min Once
Lorio 2016* [53]	Increasing knowledge and confidence in caring for people with dementia	N/A	Students	Headphones; Glasses; Shoe inserts; Gloves	12 hours 8 min Once
Tier 2 Programme [62]	Imparting basic skills relevant for people in contact with people with dementia; Increasing knowledge and confidence	National health service system “Trust”	Healthcare professionals; Other professionals (such as managers)	Ageing suits	2 days N/A Once
DEALTS 2 [63,64]	Placing staff into the shoes of a person with dementia to facilitate positive impact on practice	N/A	Healthcare professionals; Educators	Simulation instructions; Handouts; (Party: additional simulation materials, such as goggles)	1 day N/A Once
Alzheimer’s Australia Vic Virtual Dementia Experience^™^ [77,78]	Experiencing the cognitive and perceptual difficulties of people with dementia	Public (access via learning centre; dissemination via Dementia Australia)	Students	Equipment for multisensory, virtual simulation of light, sound, colour, and visual content (not otherwise specified)	1,5 hours N/A Once
Kimzey 2021* [51]	Experiencing altered sensory perceptions that simulate what it feels like for a person with dementia	University	Students	Patented devices (VDT materials)	N/A 8 min Once
Sung 2022* [84]	Putting someone in the shoes of a person with dementia	Healthcare facility	Healthcare professionals	VR device	3 months 5 min Once
Kimzey 2018* [49]	Increasing knowledge, attitudes, and empathy for (people with) dementia; Gaining self-confidence for dementia care	University	Students	Patented devices (VDT materials)	N/A 8 min Once
Kimzey 2020* [50]	Increasing knowledge and empathy for people with dementia	University	Students	Glasses; Headphones; Gloves	N/A 7 min Once
Dementia Care Boot Camp [54,55]	Promoting attitudes towards dementia, empathy, dementia knowledge, and confidence for dementia care	University	Students	Patented VDT devices; Materials for role-play (not otherwise specified	16 hours N/A Once
TDL [72]	Improving understanding for people with dementia	Healthcare facility	Informal caregivers	Simulation movie; VR device	N/A 13 min Once
Han 2020* [75]	Providing a realistic simulation of life with dementia	N/A	Informal caregivers; Healthcare professionals	Headphones with MP3 players; Eyewear; Gloves; VR device (Materials not otherwise specified)	N/A N/A Once

Notes:

^a^ When no name was reported, the name of the first author and the year of publication were used to designate the intervention;

^b^ related to the self-experience intervention;

^c^ only materials described during the SE component were taken into account;

^d^ we assume that participants had weekly sessions over a 12-week-period.

*Abbreviations*: AlzExp = The Alzheimer Experience; AWTD = A walk through dementia; DEALTS 2 = Dementia Education and Learning Through Simulation 2; EDIE = Educational Dementia Immersive Experience; IDO serious game = Innovative Digital Training Opportunities on Dementia for Direct Care Workers serious game; min = minutes: N/A = not applicable/not available; SE = self-experience; TDL = Through the D’mentia Lens; VDT = Virtual Dementia Tour.

#### Passive interventions

Within the category of passive interventions, we differentiated between film interventions and theatre interventions.

*Film interventions*. Film interventions create a cinematic scenario for all participants to experience. By showing the everyday challenges of living with dementia, the films convey dementia-specific information and present the different perspectives of the main characters. Participants explore their experiences through a reflection activity. Two film intervention programmes [60,71] were identified: ‘Barbara’s Story’ and ‘Barbara’s Evolving Story’ [60] were designed to train an entire health facility by having personnel watch a film in groups, accompanied by facilitated discussions. The online media production ‘The Alzheimer Experience’ [71] aimed to provide public education via a website. Each scene was followed by information about the content and its relation to dementia. While one report emphasised comprehensive support and guidance for participants [60], the second publication [71] did not provide any detailed information for this purpose.

*Barriers*: According to providers, the chosen dissemination channels (mainly through the nation’s Alzheimer Society) posed barriers, as they resulted in a more homogeneous target audience and the intended audience was not reached [71]. Furthermore, people tended to watch the first scenes rather than the last scenes of a film [71]. Consumers expressed time constraints with their professional work as hindering, as it resulted in lower participation than was expected by the providers [60].

*Facilitators*: From the consumers’ perspective, supportive roles (e.g., managers or dementia officers) played a crucial role in promoting the importance and maintenance of the intervention. Involving the entire organisation, having multiple ways to access the intervention, and delivering the programme over a longer period of time were described as beneficial from both consumer and provider perspectives [60]. Providers also highlighted the significance of adopting a first-person perspective in the development of a film intervention, as it enhances consumers’ understanding of the caring situation [60].

*Theatre interventions*. Theatre interventions involve live performances by (amateur) actors, allowing participants to gain an outside perspective on various aspects of dementia. The aim is to emotionally engage the audience and stimulate reflection on their professional experiences. The theatre intervention ‘I’m still here’ [41] was developed following qualitative interviews with individuals living with dementia. Experiences of individuals across all stages of dementia—from initial diagnosis to admission to a long-term care—were included. Information on facilitation processes or support offered to participants was not available.

*Barriers and facilitators*: None reported.

#### Interactive interventions

Within the modality of interactive interventions, we distinguished between physical simulation and role-play interventions.

*Physical simulation interventions*. Under physical simulation interventions, we grouped three intervention programmes that aimed to simulate the symptoms of dementia using tools that physically alter perceptions. Participants are asked to perform various tasks and activities using these tools. Physical simulation interventions help to improve understanding of the everyday challenges faced by individuals with dementia. The interaction occurs on multiple levels—the simulated environment, the tools, and the participants themselves, allowing for an increased sense of empathy and awareness. Four publications [73–76] reported on an intervention programme entitled ‘Dementia Live^*TM*^’, originally developed in the USA, and with South Korean adaptations. The entire programme consists of three sessions (preparation, simulation, and empowerment), with participants testing materials designed to mimic sensory, perceptual, or cognitive distortions in the first phase. Facilitation by a trained coach was reported for two cases [73,74].

Another publication [47] reported on a similar 10-minute self-experience intervention programme to raise awareness of the physical and cognitive changes experienced by people with dementia. No information was provided about facilitation processes.

The ‘Virtual Dementia Tour^®^’ (VDT^®^) was noted as another intervention programme in nine reports [46,48,52,56,57,66–68,80]. One report [57] referred to the VDT^®^ but outlined an adapted version tailored to national contexts. Trained facilitators were the most common interventionists [46,48,52], with some having prior experience in running the self-experience programme [56]. Five reports did not provide data on this subject [57,66–68,80].

*Barriers*: From the providers’ perspective, physical simulation exercises are considered as resource-intensive due to the need for trained facilitators and technical support [66]. From the consumers’ perspective, more time is required compared to conventional learning methods to cover various aspects of dementia and create a comprehensive understanding of the disease [75]. Consumers reported experiencing stress during the training [56], thus highlighting the need for additional educational sessions following the simulation [75]. Time conflict with work was identified as a limiting factor by consumers [47].

*Facilitators*: Both providers and consumers have expressed the benefits of exchanging experiences and engaging in debriefing sessions [52,56,75], which should be possible at any time and carried out by people with relevant expertise [56].

*Role-play interventions*. Role-play interventions aim to have participants act out coherent patient scenarios in a predefined setting that approximates the environment in which people with dementia may live. These interventions utilise live model simulation as a teaching tool in educational settings. Two publications [44,81] reported on intervention programmes. One of them [81] utilised two different scenarios related to dementia, while the second programme [44] incorporated two scenarios with only one involving caring for a person with dementia. In both cases, the participants took turns playing different roles. Specific information regarding facilitation was not provided in either interventions.

*Barriers*: Providers pointed out the high resource intensity and the need for intensive supervision of the amateur actors [44] as well as the importance of joint reflection with participants who have limited experience of supporting people with dementia [81].

*Facilitators*: Student participation in a live model simulation was seen as beneficial due to the constant availability and therefore low resource requirements [44]. Briefings and clear role descriptions were identified as key elements in creating a realistic simulation [81]. The consumers expressed positive feedback, reporting that experiential learning could be a suitable approach as an alternative to clinical practice [44].

#### Immersive interventions

We categorised three types of immersive interventions: Game interventions, Mixed Reality interventions, and Virtual Reality interventions.

*Game interventions*. This type of intervention aims to develop a computer game in terms of a serious game, which simulates dementia and relevant aspects associated with the condition. The concept is based on existing games where each player takes care of their own virtual entity. Social interactions and a simplified needs system challenges the players while creating a non-traditional environment to promote an empathetic understanding of dementia. One report [82] described an intervention programme that can be classified as a game intervention. The game provides feedback, making it an experiential learning activity. Data on support and facilitation processes are missing.

*Barriers and facilitators*: None reported.

*Virtual reality interventions*. Virtual Reality interventions involve a technical device that stimulates participants’ perceptions and senses. The practices can be delivered through various channels, including Virtual Reality goggles, screens, audio overlay, and other systems. Five reports [45,57,58,61,79] describe five different Virtual Reality programmes. Three of them [45,61,79] utilised smartphone apps (in combination with VR goggles) to create an immersive first-person simulation that allows participants to directly experience situations people with dementia may encounter. In some cases, participants were given the option to choose between different scenarios. Another report [58] describes an intervention programme where participants were completely immersed in a virtual environment and had to solve tasks, including misdirection to create a sense of confusion. The VR simulation in this case was controlled via screen, mouse, and keyboard. One further report [57] discusses the implementation of the ‘Virtual Dementia Tour’ (VDT) through VR, utilising materials such as displays, VR goggles, headphones, and handheld remote controls to manipulate objects and complete tasks. Detailed information on facilitation was lacking in most publications, however, one report mentioned the potential for debriefing participants [79].

*Barriers*: Providers noted that the use of VR goggles can be challenging for participants who wear glasses or have visual impairment, as well as for those prone to motion sickness [45]. They also pointed out that the initial cost of equipment and software can be prohibitive [45].

*Facilitators*: Previous exposure to VR experience was seen as beneficial, as it improved participants’ perception of the intervention [45].

*Mixed reality interventions*. Mixed Reality interventions involve a combination of VR and real-life conditions created through specific materials. Two studies and one protocol [42,69,70] reported on the intervention programme entitled ‘Into D’mentia’, in which a portable cabin replicates a home environment. Audio-visual elements enable participants to interact through video projections, accompanied by the voice of an informal caregiver using a speaker vest with microphones. Information is missing regarding facilitation.

*Barriers*: Providers pointed out the high cost of Mixed Reality interventions, making it potentially unaffordable for individual caregivers [70].

*Facilitators*: Consumers emphasised the importance of group meetings or follow-up sessions to share experiences, as well as the need to portray the progression of dementia in self-experience interventions [70].

#### Multicomponent interventions

Interventions consisting of a combination of self-experience practices, as well as interventions comprising both self-experience and other learning methods were subsumed as ‘multicomponent interventions’.

*Combination of self-experience practices*. This type of intervention involves combining at least two self-experience practices to improve outcomes by combining the practices and/or providing a more comprehensive account of dementia. Four reports each described four different intervention programmes, all of which combined passive and interactive measures [43,59,65,83]. The passive components included films or short case vignettes [43,65,83], with one intervention programme incorporating a theatrical production [59]. The interactive practices, which followed the passive practices in all cases, involved group exercises such as physical simulations [59,65,83] and/or role-plays [43,59,65]. Three multicomponent interventions [43,59,65] included elements of reflection, dialogue, and debriefing, while one report gave no detailed information on participant facilitation [83].

*Barriers*: Providers identified tension between providing information and entertainment during the intervention [59], and the resource-intensive nature of the interventions as barriers compared to conventional learning methods [65]. Consumers reported the risk of emotional reactions such as fear, anger, frustration, and helplessness during the interventions [83].

*Facilitators*: Enabling factors from the provider perspective included offering flexible sessions to accommodate staff [43], availability of resources [59], collaboration with local healthcare providers [59], facilitator’s debriefing skills and experience [65], as well as time to practice the skills in a safe environment [65].

*Combination of self-experience practices with other learning methods*. These intervention programmes combine self-experience practices with other (conventional) teaching and learning methods, such as lectures. A total of 14 reports, including one study protocol [49–51,53–55,62–64,72,75,77,78,84], describe eleven intervention programmes. Among these programmes, nine used one self-experience practice, while two programmes [54,55,77,78] incorporated two components (which comprised either role-play and virtual dementia tour, or theatre- and VR-based simulation). All the intervention programmes combined self-experience practices with knowledge transfer, primarily through didactic lectures (classroom- or online-based), videos, and/or case vignettes. Some programmes integrated guest speakers, including individuals living with dementia [54,55,62], or clinical placements [53–55]. Additionally, debriefing measures in the form of discussion and reflection (either group-based or individual) were described in all programmes.

*Barriers*: The fact that the interventions may sometimes cause anxiety and stress [54,55] was seen as a hindering factor from the consumers’ perspective. Providers highlighted that involving external actors in the training creates a level of dependency but also incurs corresponding costs [64]. They also noted that the interventions often focus on the early stage of Alzheimer’s disease, while other forms and stages of dementia should be considered [72].

*Facilitators*: Facilitating factors identified from the provider perspective include mutual support among participants [64], face-to-face contact with people with dementia (when internships are not feasible for students as part of their practical training) [53], either face-to-face [62] or hybrid training programmes [72]. Additionally, consecutive days of training [62], comprehensive and coordinated content integrated into curricula [50], team-based approaches [54], reflection and discussion sessions [75,77,78], affordability [72], and ease of access [72,84] were all identified as facilitators. Consumers highlighted the benefit of having a short conversation or reflection immediately after watching a film [72].

### Learning outcomes

**Fig 2:**
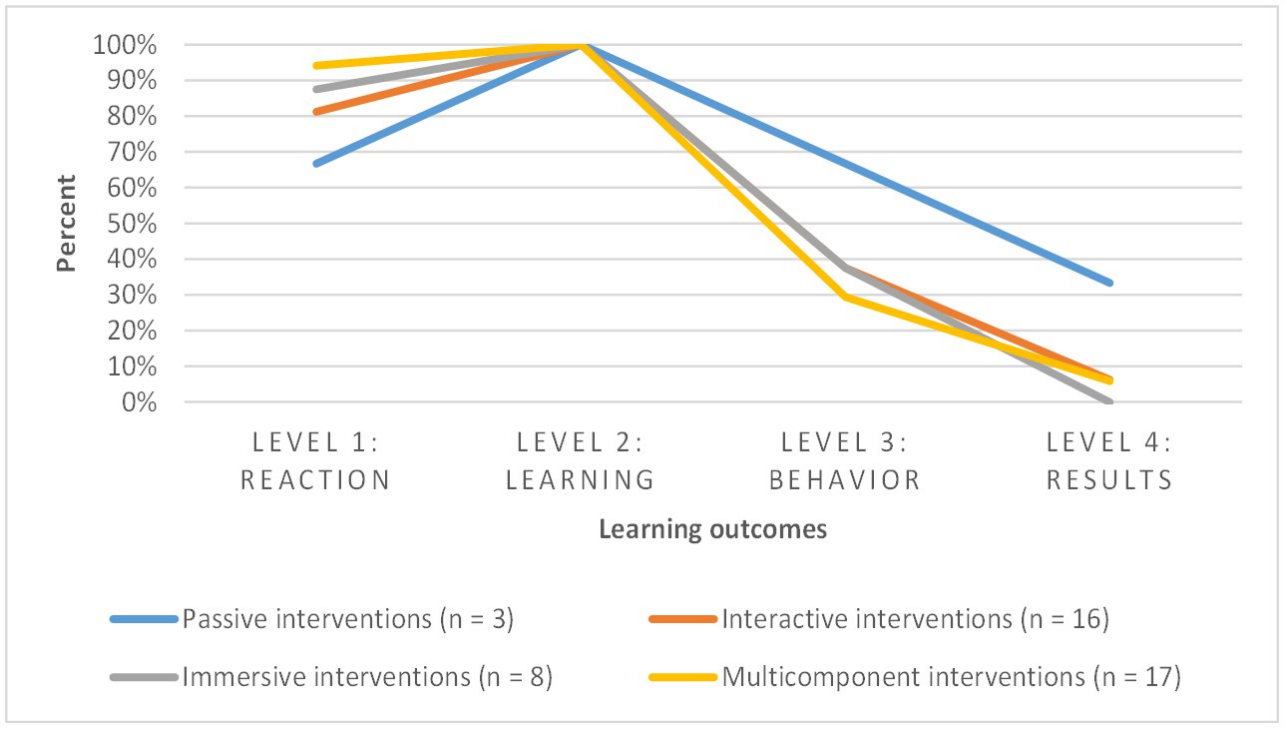
Percentage of reported learning outcomes depending on the modality of the interventions.

Fig 2 displays the percentage of reported learning outcomes based on the modality of the interventions. A table containing all intended and reported learning outcomes (categorised by modality and type of interventions) can be found in S1 File. “Intended” outcomes refer to ones that were targeted for exploration in the research questions and aims of a paper. "Reported" learning outcomes indicate the outcomes that were ultimately reported in the results section of the publication.

Publications investigating participants’ experiences with and (emotional) reactions to the self-experience interventions were classified as level 1 outcomes, according to the Kirkpatrick model [37,38]. Additionally, level 1 outcomes were considered when ‘empathy’ was specified as an outcome, as it encompasses several dimensions, including emotional response [40]. Overall, we included 38 out of 44 publications that reported on level 1 outcomes (86%; [42,43,45,46,48–62,64–68,70–76,78–80,83,84]). These outcomes were measured either qualitatively through interviews or written reflections, or quantitatively using validated instruments (e.g., the Interpersonal Reactivity Index; IRI), or self-developed questionnaires.

Level 2 outcomes referred to knowledge gain, acquired skills, changed attitudes towards dementia, and empathy resulting from the self-experience interventions [37,38]. All the reports (44 out of 44; [41–62,64–68,70–84]) in this scoping review reported level 2 outcomes, assessed through focus groups, interviews, or questionnaires.

Skills and behaviour resulting from the training (level 3; [37,38]) were reported in 15 publications describing 16 intervention programmes (36%; [41–43,59–62,64,66,67,73–75,80,82]). Many reports mentioned changed strategies for caring for people with dementia, the influence of the intervention programme, and the facilitating and inhibiting factors in practice when implementing new behaviours. The results were evaluated primarily through focus groups and interviews.

Level 4 outcomes referred to targeted outcomes and practice results. In our case, only three publications (7%; [48,60,64]) reported on cultural change in a healthcare facility and disseminating the respective self-experience training in education or nursing practice. These findings were obtained through either qualitative focus group interviews or quantitative data collection. In one case, information is missing.

### Methodological quality of sources of evidence

The methodological quality of the included reports varied widely. In most cases, the included publications of qualitative design fulfilled most, if not all, of the assessed criteria. However, in some instances, the lack of information in the study reports made it difficult to conduct a thorough appraisal.

Studies with a quantitative study design had qualitative shortcomings in the methodological approach, leading to limitations in the validity of these studies. In particular, information about the target group was often missing, as were measures to control for possible confounding. Additionally, the use of self-developed and non-validated instruments, as well as incomplete outcome data, frequently led to the downgrading of quality. The four included (Cluster-) RCTs lacked information on patient adherence in the assigned group in all cases as well as mostly on the blinding and randomisation processes. However, across all included reports, no imbalances in baseline characteristics of study groups were evident that could have influenced outcomes.

The reports that combined two data strands did not exhibit high quality in any of the included cases. Specifically, the rationale for using two data strands and the intention to apply a mixed-methods design were often lacking. Furthermore, the lack of integration between the data strands in the mixed-methods designs also led to a downgrade in the assessed quality. In cases where only two data strands were combined side by side, the critical appraisal of the 5^th^ section (“Mixed methods”) of the MMAT was not applied.

## Discussion

We reviewed various types of self-experience practices in dementia training, focusing on (future) professional and informal caregivers. We integrated 44 reports describing 30 intervention programmes for this purpose. The interventions were categorised into four different modalities and a total of nine intervention types. They ranged from passive interventions without technological components (e.g., role-playing interventions) to high-tech interventions, such as Virtual or Mixed Reality. A significant portion comprised multicomponent interventions. By analysing the reports, we were able to portray differences in the design, application, and in some aspects of the implementation of the interventions and their components. Our findings show considerable heterogeneity across intervention programmes in terms of objectives, target groups, dosage (frequency and duration), and participant support, notwithstanding the similarity of their underlying rationale.

The publications suggest that self-experience practices in the context of dementia represent a valuable learning and teaching strategy, as they provide participants with the chance to step into the shoes of a person with dementia [25]. Our review emphasises the various opportunities available to simulate this experience. Although these interventions encompass different modalities and types, they seem to share the same underlying rationale—that is to foster a deeper understanding of the lived experience of individuals with dementia through a shift in perspective. This may enable participants to gain greater empathy and knowledge of the needs and challenges faced by people with dementia, which in turn may enhance participants’ ability to provide person-centred dementia care.

Our review highlights several facilitating and hindering factors for the implementation of different types of self-experience practices in the dementia training context. Some of these factors align with established best practice for experiential learning in general, as demonstrated in a systematic review [23]. Furthermore, the ‘*What works in dementia education and training’* study also addressed this topic within comprehensive research [24,26,27]. However, the research focused on general training methods and recommendations within the dementia context. A systematic review [27] conducted as a part of the study, with reports up until 2015, aligns with our findings in emphasising the importance of sufficient time for debriefing and discussion in experiential learning, as well as the need for experienced trainers or facilitators. However, our specific focus on self-experience practices in dementia showed some key differences from the previous recommendations made for generic training methods. For instance, for generic dementia training courses, an overall duration of more than 8 hours with individual training sessions of at least 90 minutes is suggested to be effective, as this may lead to better outcomes (e.g., reduction of stress and burden, job satisfaction) for participants compared to shorter training courses [27]. In contrast, the interventions using self-experience practices in the current review were commonly described as being short in time, often lasting only a few minutes. This seemed to be justified by the intensity of experiences reported by participants.

To date, reviews have not yet highlighted the specifications of experience-based learning in the context of dementia, such as high resource intensity (potentially due to costs of materials and technology), professional actors, and continued facilitation led by trained moderators. Additionally, there is the potential for distress and anxiety caused by training with self-experience practices, which necessitates a safe environment for the participants. The self-experience interventions identified in our review were frequently conducted at the participants’ workplace or familiar learning environments (e.g., universities), thus taking place in their accustomed surroundings, with limited use of external training facilities.

We applied Kirkpatrick’s model of training programme evaluation [37,38] to provide an overview of the learning outcomes of each self-experience programme included. The analysis revealed that despite varying assumed mechanisms among the different modalities, the distribution of learning outcomes remained consistent across all intervention modalities. Most of the identified reports focused on evaluating the participants’ immediate reactions (level 1) and outcomes on participants’ knowledge and skills (level 2) in self-experience practices. However, there was limited consideration given to evaluating the impact of training on participants’ behaviour (level 3) and the environment of caregiving practice (level 4). As a result, the learning outcomes observed in these interventions primarily show effects on participants in the short-term, with little consideration of long-term effects on people with dementia and practice. This finding is consistent with other reviews, for example on evidence-based practices in healthcare, where educational formats often focus on short-term effects on knowledge and skills rather than long-term changes in behaviour or on establishing a culture of best practice in organisations [85].

This gap in the evaluation of self-experience practices is particularly relevant in the context of person-centred care for people with dementia. Evaluating the impact of self-experience practices on participants’ behaviour and the practice environment can offer valuable insights into how these programmes might contribute to the application of person-centred dementia care. While publications describe a positive impact on participants’ skills and knowledge, there is uncertainty about whether these extend to practice. It is emphasised that skills acquired through experiential trainings are not retained over time without continuous practice [86]. Therefore, it is crucial to conduct further research to investigate the long-term impact of self-experience practices on behaviour and the practice environment. This would provide a more comprehensive understanding of the sustained effectiveness of these programmes and the potential need for continued support and reinforcement to ensure the transfer of acquired skills into different practice settings.

Kirkpatrick’s model is a frequently used evaluation model for training programmes in healthcare [87]. It has been criticised for the overemphasis on the hierarchy of levels, the neglect of the lower levels of the evaluation model, and the implication of causal linkage between levels [88,89]. Furthermore, it should be noted that different interpretations of Kirkpatrick’s levels exist in the literature [90]. While acknowledging the criticisms, we considered this approach as helpful for our study, as the model has been used in other significant reviews within the healthcare sector [26], thereby enhancing comparability among publications.

Our findings are broadly in line with previous findings in the field of experiential learning. A review on 153 resources within the general healthcare context reported positive outcomes for simulation compared to other didactic teaching strategies, such as conventional methods [23]. An important characteristic of experiential learning is the participants’ first-person experience, which makes self-experience practices particularly valuable for training caregivers. However, Meyer et al. [56] emphasise the need for caution, as participants may mistakenly believe they have fully experienced what it is like to live with dementia, overlooking the inherent heterogeneity of symptoms and experiences of dementia. Hence, the integration of training measures with knowledge transfer is crucial in the context of self-experience practices [56].

The ethical aspects related to self-experience practices are particularly relevant in the context of person-centred dementia care. As these interventions aim to provide participants with a deeper understanding of the lived experience of (people with) dementia through a shift in perspective. As such, it is essential to consider the ethical implications of such experiences. Participants are immersed in self-experience practices that attempt to recreate the challenges and emotions faced by people with dementia. This immersive and emotionally intense nature of self-experience practices necessitates careful consideration of the well-being of both the participants and the individuals with dementia who are represented in the simulations.

Ethical considerations should also be extended to the use of technology, such as VR, in self-experience interventions. The discussion has gained increased attention in recent years, particularly due to advancements in technology. Our analysis of publication trends indicates a growing use of self-experience practices and an increased utilisation of high-tech approaches since 2016 (available in S1 File). For instance, the use of virtual embodiment has the potential to directly influence or modify emotions, behaviours, attitudes, values, or beliefs [91,92]. VR enables individuals to virtually inhabit the body of another person [92], providing them with an experience that feels authentic despite being virtual and simulation-based. Therefore, it is crucial to approach the utilisation of these technologies in dementia education and training with caution and sensitivity. However, there is a risk of depicting situations that could potentially cause psychological harm [92].

Ensuring that participants are adequately prepared and supported is essential. Since we found limited data on participant guidance and authors’ engagement with ethical considerations, these should be given greater consideration in the development, implementation, and in the evaluation of self-experience practices. This includes the need to address varying levels of digital literacy, which present challenges in the development of appropriate tools. To address all these challenges, it is advisable to develop ethical guidelines for the development of self-experience interventions that can be universally applied but also include specific practices. These guidelines should not only include technological aspects, but also ethical considerations such as potential impact on the privacy and autonomy of participants. Integrating these aspects into the development, implementation and evaluation of self-experience practices is crucial to avoid ethical conflicts and to safeguard the well-being of both learners and individuals with dementia, as has already been done for certain self-experience practices [93].

Our review has addressed research areas summarised in a previous review [23] by capturing barriers in the implementation of self-experience practices in dementia training and by providing an overview of organisational aspects related to the different types of these interventions. This investigation underscores the critical role of identifying and analysing barriers and facilitators to enhance the effectiveness of future training programmes. Our study reveals that a multitude of factors can influence the successful implementation of such programmes, including organisational, technological, and ethical considerations.

Other reviews addressing barriers and facilitators of dementia-specific interventions yield similar results, particularly at the meso level of implementing organisations. Reports on the implementation of eHealth interventions [94] highlight financial and time constraints, and the integration of the intervention into existing care systems. High staff turnover and the perceived time and workload pressure are described as organisational barriers to the implementation of complex interventions in long-term care facilities [95]. Conversely, supportive factors identified [95] include management support, adequate resources, and support from relevant stakeholders, which are also reflected in our study.

We found a varying quality of evidence and a lack of methodological rigour in some of the included publications, such as the use of a pre-post design lacking a comparative analysis of the effectiveness of different training methods. Information on blinding and randomisation in randomised controlled trials was often missing, making it not possible to correctly assess the risk of bias. Data strands in mixed-methods studies were not appropriately integrated, resulting in the mere juxtaposition of data without merging.

In summary, the studies included in this review predominantly focused on the effectiveness of interventions. However, there is a lack of process evaluation studies examining the implementation process and its effectiveness. A similar result was found in another review on technology-based counselling interventions in dementia [96], indicating that adherence to the Medical Research Council Framework for developing and evaluating complex interventions [19,20] is not yet sufficient.

### Strengths and limitations

Our scoping review encompassed a broad spectrum of topics in the context of dementia training and integrated insights from latest publications, thereby updating existing works. The use of Kirkpatrick’s model allowed for a structured assessment of the intended and reported outcomes of self-experience practices, as it is a comprehensive model that covers all essential areas of outcomes.

The inclusion of a wide range of designs has revealed a comprehensive database; however, caution is necessary when interpreting the results. The classification of intervention programmes in terms of “modalities” and “types” may pose a limitation, as it could lead to overlap if an intervention can be assigned to multiple categories. Furthermore, the diversity of interventions may not be adequately captured by the categorisation, potentially failing to notice relevant aspects of the specific interventions. We attempted addressing this through discussions within the research team.

## Conclusions

Various interventions incorporate self-experience practices as a component of comprehensive training or form the entire design of training programmes focused on this methodology, with the use of technological tools steadily increasing in recent years.

Self-experience often refers to and engages the individual participant. However, the impact at an organisational level and especially the results for individuals living with dementia in terms of receiving high-quality, person-centred care are not typically reported, despite the rationale for such training approaches suggesting otherwise.

Our review specifically emphasises the unique considerations and challenges associated with self-experience practices in the context of dementia. Furthermore, our work can help identify potential approaches for the development of training programmes or strategies in the field of dementia care, as it provides insights into evaluated practices and methods. As demonstrated by the comprehensive presentation of the diverse interventions in this review, (future) complex interventions should be implemented and evaluated while considering ethical aspects.

## Supporting information

S1 File Supporting information on raw data and additional analysis result. (DOCX)
