# Correction: Exploring self-experience practices in dementia care: A scoping review

**DOI:** 10.1371/journal.pone.0327909

**Published:** 2025-07-09

**Authors:** Janina Wittmann, Anja Bieber, Joanne Carroll, Kealan Forristal, Louise Hopper, Niels Janssen, Gabriele Meyer, Marianna Riello, Marjolein de Vugt, Dorothee Bauernschmidt

There is an error in reference 5. The correct reference is: McCormack B, McCance T, Martin S. What is person-centredness? In: McCormack B, McCance T, Bulley C, Brown D, McMillan A, Martin S, editors. Fundamentals of person-centred healthcare practice. Hoboken, NJ: Wiley-Blackwell; 2021. p. 13–22.
