# Supplementary material for: Exploring self-experience practices in dementia care: A scoping review
Source: PLoS One. 2024 May 7;19(5):e0302929. doi: 10.1371/journal.pone.0302929 (PMC11075864; doi:10.1371/journal.pone.0302929)
Supplement: S1 File — (DOCX) [file pone.0302929.s001.docx]

**S1 File**

**Supporting information on raw data and additional analysis results**

[Chapter A: Preferred Reporting Items for Systematic reviews and Meta-Analyses extension for Scoping Reviews (PRISMA-ScR) Checklist 2](#_Toc162025581)

[Chapter B: Search strategies 5](#_Toc162025582)

[Chapter C: Critical Appraisal using Mixed Methods Appraisal Tool 10](#_Toc162025583)

[Chapter D: Tabular presentation of publication trend by modality of intervention* 113](#_Toc162025584)

[Chapter E: Graphical presentation of publication trend by modality of intervention^*,***^ 114](#_Toc162025585)

[Chapter F: Barriers and Facilitators per intervention type* 115](#_Toc162025586)

[Chapter G: Intended and reported learning outcomes of the included reports (categorised by modality and type of intervention) 116](#_Toc162025587)

[References 117](#_Toc162025588)

## Chapter A: Preferred Reporting Items for Systematic reviews and Meta-Analyses extension for Scoping Reviews (PRISMA-ScR) Checklist

| **SECTION** | **ITEM** | **PRISMA-ScR CHECKLIST ITEM** | **REPORTED ON PAGE #** |
| --- | --- | --- | --- |
| **TITLE** | | | |
| Title | 1 | Identify the report as a scoping review. | 1 |
| **ABSTRACT** | | | |
| Structured summary | 2 | Provide a structured summary that includes (as applicable): background, objectives, eligibility criteria, sources of evidence, charting methods, results, and conclusions that relate to the review questions and objectives. | 2 |
| **INTRODUCTION** | | | |
| Rationale | 3 | Describe the rationale for the review in the context of what is already known. Explain why the review questions/objectives lend themselves to a scoping review approach. | 6 |
| Objectives | 4 | Provide an explicit statement of the questions and objectives being addressed with reference to their key elements (e.g., population or participants, concepts, and context) or other relevant key elements used to conceptualize the review questions and/or objectives. | 6, 7 |
| **METHODS** | | | |
| Protocol and registration | 5 | Indicate whether a review protocol exists; state if and where it can be accessed (e.g., a Web address); and if available, provide registration information, including the registration number. | 3, 7 |
| Eligibility criteria | 6 | Specify characteristics of the sources of evidence used as eligibility criteria (e.g., years considered, language, and publication status), and provide a rationale. | 8 |
| Information sources* | 7 | Describe all information sources in the search (e.g., databases with dates of coverage and contact with authors to identify additional sources), as well as the date the most recent search was executed. | 8, 9 |
| Search | 8 | Present the full electronic search strategy for at least 1 database, including any limits used, such that it could be repeated. | 8, 9 |
| Selection of sources of evidence† | 9 | State the process for selecting sources of evidence (i.e., screening and eligibility) included in the scoping review. | 9 |
| Data charting process‡ | 10 | Describe the methods of charting data from the included sources of evidence (e.g., calibrated forms or forms that have been tested by the team before their use, and whether data charting was done independently or in duplicate) and any processes for obtaining and confirming data from investigators. | 9, 10 |
| Data items | 11 | List and define all variables for which data were sought and any assumptions and simplifications made. | 9, 10 |
| Critical appraisal of individual sources of evidence§ | 12 | If done, provide a rationale for conducting a critical appraisal of included sources of evidence; describe the methods used and how this information was used in any data synthesis (if appropriate). | 10 |
| Synthesis of results | 13 | Describe the methods of handling and summarizing the data that were charted. | 10, 11 |
| **RESULTS** | | | |
| Selection of sources of evidence | 14 | Give numbers of sources of evidence screened, assessed for eligibility, and included in the review, with reasons for exclusions at each stage, ideally using a flow diagram. | 11 |
| Characteristics of sources of evidence | 15 | For each source of evidence, present characteristics for which data were charted and provide the citations. | 11, 12 |
| Critical appraisal within sources of evidence | 16 | If done, present data on critical appraisal of included sources of evidence (see item 12). | 27 |
| Results of individual sources of evidence | 17 | For each included source of evidence, present the relevant data that were charted that relate to the review questions and objectives. | 12-26 |
| Synthesis of results | 18 | Summarize and/or present the charting results as they relate to the review questions and objectives. | 12-18 |
| **DISCUSSION** | | | |
| Summary of evidence | 19 | Summarize the main results (including an overview of concepts, themes, and types of evidence available), link to the review questions and objectives, and consider the relevance to key groups. | 27, 28 |
| Limitations | 20 | Discuss the limitations of the scoping review process. | 33 |
| Conclusions | 21 | Provide a general interpretation of the results with respect to the review questions and objectives, as well as potential implications and/or next steps. | 33, 34 |
| **FUNDING** | | | |
| Funding | 22 | Describe sources of funding for the included sources of evidence, as well as sources of funding for the scoping review. Describe the role of the funders of the scoping review. | 7 |

JBI = Joanna Briggs Institute; PRISMA-ScR = Preferred Reporting Items for Systematic reviews and Meta-Analyses extension for Scoping Reviews.

* Where *sources of evidence* (see second footnote) are compiled from, such as bibliographic databases, social media platforms, and Web sites.

† A more inclusive/heterogeneous term used to account for the different types of evidence or data sources (e.g., quantitative and/or qualitative research, expert opinion, and policy documents) that may be eligible in a scoping review as opposed to only studies. This is not to be confused with *information sources* (see first footnote).

‡ The frameworks by Arksey and O’Malley (6) and Levac and colleagues (7) and the JBI guidance (4, 5) refer to the process of data extraction in a scoping review as data charting*.*

§ The process of systematically examining research evidence to assess its validity, results, and relevance before using it to inform a decision. This term is used for items 12 and 19 instead of "risk of bias" (which is more applicable to systematic reviews of interventions) to include and acknowledge the various sources of evidence that may be used in a scoping review (e.g., quantitative and/or qualitative research, expert opinion, and policy document).

*From:* Tricco AC, Lillie E, Zarin W, O'Brien KK, Colquhoun H, Levac D, et al. PRISMA Extension for Scoping Reviews (PRISMAScR): Checklist and Explanation. Ann Intern Med. 2018;169:467–473. [doi: 10.7326/M18-0850](http://annals.org/aim/fullarticle/2700389/prisma-extension-scoping-reviews-prisma-scr-checklist-explanation).

## Chapter B: Search strategies

**Search strategy for Cochrane Library**

Date: 16.11.2022

| # | Entry | Hits |
| --- | --- | --- |
| 1 | (dement* OR alzheimer* OR "mild cognitive impairment*" OR MIC OR "cognitive dysfunct*" OR "cognitive impairment" OR "neurodegenerative disease*" OR "neurodegenerative disorder*" OR "cognitive function*" OR "cognitive disorder*" OR "memory disorder*"):ti,ab,kw OR mh dementia | 40.225 |
| 2 | ("simulation training*" OR "augmented realit*" OR simulation* OR "affective learning" OR training* OR practice OR intervention* OR construct* OR "mixed realit*" OR education* OR "experience-taking" OR "experience-based" OR "simulation-based" OR experiential*):ti,ab,kw OR mh simulation training | 642.922 |
| 3 | ("virtual dementia tour" OR virtual* OR "virtual dementia experience" OR "virtual tour*" OR "virtual realit*" OR "VR"):ti,ab,kw OR mh virtual reality | 13.202 |
| 4 | ("role play*" OR "role game*" OR "role playing game*"):ti,ab,kw OR mh role playing | 665 |
| 5 | ("theatre laborator*" OR drama OR theatre OR theater):ti,ab,kw | 2096 |
| 6 | ("self experience*" OR "self awareness*" OR "self perception*" OR "empathy" OR "social presence" OR "embodied knowledge" OR "being-there" OR "compassion*" OR "person centred attitude" OR "person centered attitude" OR "person centered*" OR "person centred*"):ti,ab,kw | 105.099 |
| 7 | ("caregiver*" OR "carer*" OR "health personnel" OR "health care professional*" OR "health professional*" OR "nurse*" OR "physician*" OR "doctor*" OR "GP" OR "general practitioner*" OR "psychologist*" OR "psychotherapist*" OR "family caregiver*" OR "informal caregiver*" OR "nursing student*" OR "medical student*" OR "social care professional*" OR "social worker*" OR "physiotherapist*" OR "occupational therapist*" OR "therapist*" OR "professional*" OR "health student*" OR "health care student*" OR "clinician*"):ti,ab,kw OR MH caregivers OR mh health personnel | 125.239 |
| 8 | #2 OR #3 OR #4 OR #5 | 648.937 |
| 9 | #1 AND #8 AND #6 AND #7 | 1.072 |
| 10 | #1 AND #8 AND #6 AND #7 Filters: from 2010 - 2022 | 1.034 |

**Search strategy MEDLINE via PubMed**

Date: 16.11.2022

| # | Entry | Hits |
| --- | --- | --- |
| 1 | "dementia"[MeSH Terms] OR "dement*"[Title/Abstract] OR "alzheimer*"[Title/Abstract] OR "mild cognitive impairment*"[Title/Abstract] OR "MIC"[Title/Abstract] OR "cognitive dysfunct*"[Title/Abstract] OR "cognitive impairment"[Title/Abstract] OR "neurodegenerative disease*"[Title/Abstract] OR "neurodegenerative disorder*"[Title/Abstract] OR "cognitive function*"[Title/Abstract] OR "cognitive disorder*"[Title/Abstract] OR "memory disorder*"[Title/Abstract] | 511.124 |
| 2 | "simulation training"[MeSH Terms] OR "simulation training*"[Title/Abstract] OR "augmented realit*"[Title/Abstract] OR "simulation*"[Title/Abstract] OR "affective learning"[Title/Abstract] OR "training*"[Title/Abstract] OR "practice"[Title/Abstract] OR "intervention*"[Title/Abstract] OR "construct*"[Title/Abstract] OR "mixed realit*"[Title/Abstract] OR "education*"[Title/Abstract] OR "experience-taking"[Title/Abstract] OR "experience-based"[Title/Abstract] OR "simulation-based"[Title/Abstract] OR "experiential*"[Title/Abstract] | 3.764.479 |
| 3 | "virtual reality"[MeSH Terms] OR "virtual dementia tour"[Title/Abstract] OR "virtual*"[Title/Abstract] OR "virtual dementia experience"[Title/Abstract] OR "virtual tour*"[Title/Abstract] OR "virtual realit*"[Title/Abstract] OR "VR"[Title/Abstract] | 167.068 |
| 4 | "role playing"[MeSH Terms] OR "role play*"[Title/Abstract] OR "role game*"[Title/Abstract] OR "role playing game*"[Title/Abstract] | 24.807 |
| 5 | "theatre laborator*"[Title/Abstract] OR "drama"[Title/Abstract] OR "theatre"[Title/Abstract] OR "theater"[Title/Abstract] | 14.921 |
| 6 | "self-experience*"[Title/Abstract] OR "self-awareness*"[Title/Abstract] OR "self-perception*"[Title/Abstract] OR "empathy"[Title/Abstract] OR "social presence"[Title/Abstract] OR "embodied knowledge"[Title/Abstract] OR "being-there"[Title/Abstract] OR "compassion*"[Title/Abstract] OR "person-centred attitude"[Title/Abstract] OR "person-centered attitude"[Title/Abstract] OR "person-centered*"[Title/Abstract] OR "person-centred*"[Title/Abstract] | 50.557 |
| 7 | "caregivers"[MeSH Terms] OR "health personnel"[MeSH Terms] OR "caregiver*"[Title/Abstract] OR "carer*"[Title/Abstract] OR "health personnel"[Title/Abstract] OR "health care professional*"[Title/Abstract] OR "health professional*"[Title/Abstract] OR "nurse*"[Title/Abstract] OR "physician*"[Title/Abstract] OR "doctor*"[Title/Abstract] OR "GP"[Title/Abstract] OR "general practitioner*"[Title/Abstract] OR "psychologist*"[Title/Abstract] OR "psychotherapist*"[Title/Abstract] OR "family caregiver*"[Title/Abstract] OR "informal caregiver*"[Title/Abstract] OR "nursing student*"[Title/Abstract] OR "medical student*"[Title/Abstract] OR "social care professional*"[Title/Abstract] OR "social worker*"[Title/Abstract] OR "physiotherapist*"[Title/Abstract] OR "occupational therapist*"[Title/Abstract] OR "therapist*"[Title/Abstract] OR "professional*"[Title/Abstract] OR "health student*"[Title/Abstract] OR "health care student*"[Title/Abstract] OR "clinician*"[Title/Abstract] | 1.887.486 |
| 8 | #2 OR #3 OR #4 OR #5 | 3.914.165 |
| 9 | #1 AND #8 AND #6 AND #7 | 905 |
| 10 | #1 AND #8 AND #6 AND #7 Filters: from 2010 - 2022 | 832 |
| 11 | #1 AND #8 AND #6 AND #7 Filters: from 2010 - 2022, Dutch, English, German, Italian | 821 |

**Search strategy Web Of Science Core Collection**

Date: 16.11.2022

| # | Entry | Hits |
| --- | --- | --- |
| 1 | TS=(dement* OR alzheimer* OR "mild cognitive impairment*" OR MIC OR "cognitive dysfunct*" OR "cognitive impairment" OR "neurodegenerative disease*" OR "neurodegenerative disorder*" OR "cognitive function*" OR "cognitive disorder*" OR "memory disorder*") | 604.393 |
| 2 | TS=("simulation training*" OR "augmented realit*" OR simulation* OR "affective learning" OR training* OR practice OR intervention* OR construct* OR "mixed realit*" OR education* OR "experience-taking" OR "experience-based" OR "simulation-based" OR experiential*) | 7.950.721 |
| 3 | TS=("virtual dementia tour" OR virtual* OR "virtual dementia experience" OR "virtual tour*" OR "virtual realit*" OR VR) | 336.565 |
| 4 | TS=("role play*" OR "role game*" OR "role playing game*") | 46.516 |
| 5 | TS=("theatre laborator*" OR drama OR theatre OR theater) | 96.853 |
| 6 | TS=("self experience*" OR “self awareness*" OR "self perception*" OR "empathy" OR "social presence" OR "embodied knowledge" OR "being-there" OR "compassion*" OR "person centred attitude" OR "person centered attitude" OR "person centered*" OR "person centred*") | 91.078 |
| 7 | TS=("caregiver*" OR "carer*" OR "health personnel" OR "health care professional*" OR "health professional*" OR "nurse*" OR "physician*" OR "doctor*" OR "GP" OR "general practitioner*" OR "psychologist*" OR "psychotherapist*" OR "family caregiver*" OR "informal caregiver*" OR "nursing student*" OR "medical student*" OR "social care professional*" OR "social worker*" OR "physiotherapist*" OR "occupational therapist*" OR "therapist*" OR "professional*" OR "health student*" OR "health care student*" OR "clinician*") | 1.612.246 |
| 8 | #2 OR #3 OR #4 OR #5 | 8.288.903 |
| 9 | #1 AND #8 AND #6 AND #7 | 1.149 |
| 10 | #1 AND #8 AND #6 AND #7 Filters: from 2010 - 2022 | 1.081 |
| 11 | #1 AND #8 AND #6 AND #7 Filters: from 2010 - 2022, Dutch, English, German, Italian | 1.071 |

**Search strategy CINAHL**

Date: 16.11.2022

| # | Entry | Hits |
| --- | --- | --- |
| 1 | (TI (dement* OR alzheimer* OR "mild cognitive impairment*" OR MIC OR "cognitive dysfunct*" OR "cognitive impairment" OR "neurodegenerative disease*" OR "neurodegenerative disorder*" OR "cognitive function*" OR "cognitive disorder*" OR "memory disorder*")) OR (AB (dement* OR alzheimer* OR "mild cognitive impairment*" OR MIC OR "cognitive dysfunct*" OR "cognitive impairment" OR "neurodegenerative disease*" OR "neurodegenerative disorder*" OR "cognitive function*" OR "cognitive disorder*" OR "memory disorder*")) OR (MH "Dementia") | 145.438 |
| 2 | (TI ("simulation training*" OR "augmented realit*" OR simulation* OR "affective learning" OR training* OR practice OR intervention* OR construct* OR "mixed realit*" OR education* OR "experience-taking" OR "experience-based" OR "simulation-based" OR experiential*)) OR (AB ("simulation training*" OR "augmented realit*" OR simulation* OR "affective learning" OR training* OR practice OR intervention* OR construct* OR "mixed realit*" OR education* OR "experience-taking" OR "experience-based" OR "simulation-based" OR experiential*)) OR (MH "Simulations") | 1.471.836 |
| 3 | (TI ("virtual dementia tour" OR virtual* OR "virtual dementia experience" OR "virtual tour*" OR "virtual realit*" OR VR)) OR (AB ("virtual dementia tour" OR virtual* OR "virtual dementia experience" OR "virtual tour*" OR "virtual realit*" OR VR)) OR (MH "Virtual Reality") | 46.517 |
| 4 | (TI ("role play*" OR "role game*" OR "role playing game*")) OR (AB ("role play*" OR "role game*" OR "role playing game*")) OR (MM "Role Playing") | 5.690 |
| 5 | (TI ("theatre laborator*" OR drama OR theatre OR theater)) OR (AB ("theatre laborator*" OR drama OR theatre OR theater)) | 7.825 |
| 6 | (TI (“self experience*" OR "self awareness*" OR "self perception*" OR "empathy" OR "social presence" OR "embodied knowledge" OR "being-there" OR "compassion*" OR "person-centred attitude" OR "person-centered attitude" OR "person centered*" OR "person centred*")) OR (AB (“self experience*" OR "self awareness*" OR "self perception*" OR "empathy" OR "social presence" OR "embodied knowledge" OR "being-there" OR "compassion*" OR "person-centred attitude" OR "person-centered attitude" OR "person centered*" OR "person centred*")) | 357.321 |
| 7 | (TI ("caregiver*" OR "carer*" OR "health personnel" OR "health care professional*" OR "health professional*" OR "nurse*" OR "physician*" OR "doctor*" OR "GP" OR "general practitioner*" OR "psychologist*" OR "psychotherapist*" OR "family caregiver*" OR "informal caregiver*" OR "nursing student*" OR "medical student*" OR "social care professional*" OR "social worker*" OR "physiotherapist*" OR "occupational therapist*" OR "therapist*" OR "professional*" OR "health student*" OR "health care student*" OR "clinician*")) OR (AB ("caregiver*" OR "carer*" OR "health personnel" OR "health care professional*" OR "health professional*" OR "nurse*" OR "physician*" OR "doctor*" OR "GP" OR "general practitioner*" OR "psychologist*" OR "psychotherapist*" OR "family caregiver*" OR "informal caregiver*" OR "nursing student*" OR "medical student*" OR "social care professional*" OR "social worker*" OR "physiotherapist*" OR "occupational therapist*" OR "therapist*" OR "professional*" OR "health student*" OR "health care student*" OR "clinician*")) OR (MH "Caregivers") | 1.022.002 |
| 8 | #2 OR #3 OR #4 OR #5 | 1.508.357 |
| 9 | #1 AND #8 AND #6 AND #7 | 2.455 |
| 10 | #1 AND #8 AND #6 AND #7 Filters: from 2010 - 2022 | 2.001 |
| 11 | #1 AND #8 AND #6 AND #7 Filters: from 2010 - 2022, Dutch, English, German, Italian | 1.964 |

**Search strategy International Clinical Trails Registry Platform**

Date: 01.02.2023

| Component | Term | |
| --- | --- | --- |
| Dementia | Dementia* OR  Alzheimer* | |
| Self-experience Practices | Simulation* OR  augmented realit* OR  mixed realit* OR  experiential OR  virtual* OR  role play* OR  role game* OR  drama OR  theatre* OR  theatre* OR  self experience* | |
|  | | |
| ((dementia* OR alzheimer*) AND (simulation* OR augmented realit* OR mixed realit* OR experiential OR virtual* OR role play* OR role game* OR drama* OR theater* OR theatre* OR self-experience*)) | | 11 |

**Search strategy PROSPERO**

Date: 30.01.2023

| Component | Term | |
| --- | --- | --- |
| Dementia | Dementia* OR  Alzheimer* | |
| Self-experience Practices | Simulation* OR  augmented realit* OR  mixed realit* OR  experiential OR  virtual* OR  role play* OR  role game* OR  drama OR  theatre* OR  self experience* | |
|  | | |
| ((dementia* OR alzheimer*) AND (simulation* OR augmented realit* OR mixed realit* OR experiential OR virtual* OR role play* OR role game* OR drama* OR theater* OR theatre* OR self-experience*)) | | 21 |

## Chapter C: Critical Appraisal using Mixed Methods Appraisal Tool

Hong QN, Pluye P, Fàbregues S, Bartlett G, Boardman F, Cargo M, Dagenais P, Gagnon M-P, Griffiths F, Nicolau B, O’Cathain A, Rousseau M-C, Vedel I. Mixed Methods Appraisal Tool (MMAT), version 2018. Registration of Copyright (#1148552), Canadian Intellectual Property Office, Industry Canada.

*Notes:* The following extractions are mostly quotes taken directly from the publications, comments made by authors are highlighted in italics;

The critical appraisal was conducted for all included reports (but not for study protocols);

Sorted alphabetically according to the name of the first author

| [1] Adefila A, Graham S, Clouder L, Bluteau P, Ball S. myShoes – the future of experiential dementia training? Journal of Mental Health Training, Education & Practice. 2016;11(2):91-101. | | | | | |
| --- | --- | --- | --- | --- | --- |
|  | | | | | |
| **Category of study designs** | **Methodological quality criteria** | **Responses** | | | |
|  |  | Yes | No | Can’t tell | Comments |
| Screening questions  (for all types) | S1. Are there clear research questions? | x |  |  | *Clearly and detailed formulated as research objectives:*  The study objectives were to:   - collaborate with dementia experts to identify the common features of dementia and age related sensory impairments which could be simulated; - build a prototype simulation of the common features of dementia; - user test and pilot the prototype with a multi-professional group of health and social care students; - collect pre-and post-intervention data on immediate impact on perceived confidence, competence and compassion; and - analyse data to identify process outcomes (what students have learned from the immersive experience). |
|  | S2. Do the collected data allow to address the research questions? | x |  |  | Data collection included “think aloud” technique, observations and questionnaires (pre-post; with qualitative and quantitative parts) to cover all research objectives |
|  | *Further appraisal may not be feasible or appropriate when the answer is ‘No’ or ‘Can’t tell’ to one or both screening questions.* | | | | |
| 1. Qualitative | 1.1. Is the qualitative approach appropriate to answer the research question? | x |  |  | *Yes, but no in-depth findings (e.g. experiences with impact of intervention) were desired.* |
|  | 1.2. Are the qualitative data collection methods adequate to address the research question? |  |  | x | *At this point, the researchers would probably have obtained results with a higher content through interviews or focus groups.*  All participants completed pre-and post-simulation questionnaires designed to collate opinions about dementia patients’ attitudes and behaviour, and self-perceived confidence, competence and compassion scores in relation to treating patients with dementia before and after the simulation. The pre-test questionnaires requested information about students’ previous understanding of dementia. The post-test  questionnaire included questions about the impact of the resource on the participants’  understanding of dementia and how the resource could be improved. In addition, three  standardised self-reporting questionnaires were also completed by the participants prior to taking part in the simulation. (…)  During the virtual experience each participant was recorded articulating their thoughts and views as they entered the simulation and engaged with the activities. This “think aloud” technique (Cotton and Gresty, 2007) was used to gain immediate access to the thought processes occurring during immersion. Researchers also independently observed participants’ movements and involuntary physiological responses to capture non-verbal information. |
|  | 1.3. Are the findings adequately derived from the data? |  |  | x | *No statement on this.* |
|  | 1.4. Is the interpretation of results sufficiently substantiated by data? | x |  |  | *Interpretations are underpinned by quotes and further examples.* |
|  | 1.5. Is there coherence between qualitative data sources, collection, analysis and interpretation? |  |  | x | *No statement possible due to incomplete information in the paper.* |
| 2. Quantitative randomized controlled trials | 2.1. Is randomization appropriately performed? |  |  |  |  |
|  | 2.2. Are the groups comparable at baseline? |  |  |  |  |
|  | 2.3. Are there complete outcome data? |  |  |  |  |
|  | 2.4. Are outcome assessors blinded to the intervention provided? |  |  |  |  |
|  | 2.5 Did the participants adhere to the assigned intervention? |  |  |  |  |
| 3. Quantitative non-randomized | 3.1. Are the participants representative of the target population? |  | x |  | *Probably not representative of all students, as participants were self-selected.*  55 self-selecting students studying health and social care degrees, including adult and mental health nurses, clinical psychologists, occupational therapists, paramedics, physiotherapists and social workers. |
|  | 3.2. Are measurements appropriate regarding both the outcome and intervention (or exposure)? | x |  |  | *The variables to be measured were clearly defined and presumably measured accurately and are thus suitable to answer the research question. In some cases, validated scales were used.* |
|  | 3.3. Are there complete outcome data? | x |  |  | *Probably, but the number of participants (e.g. in table 1) is not specified.* |
|  | 3.4. Are the confounders accounted for in the design and analysis? |  | x |  | *No methods for controlling confounding factors were described.* |
|  | 3.5. During the study period, is the intervention administered (or exposure occurred) as intended? |  |  | x | *No statement on this.* |
| 4. Quantitative descriptive | 4.1. Is the sampling strategy relevant to address the research question? |  |  |  |  |
|  | 4.2. Is the sample representative of the target population? |  |  |  |  |
|  | 4.3. Are the measurements appropriate? |  |  |  |  |
|  | 4.4. Is the risk of nonresponse bias low? |  |  |  |  |
|  | 4.5. Is the statistical analysis appropriate to answer the research question? |  |  |  |  |
| 5. Mixed methods | 5.1. Is there an adequate rationale for using a mixed methods design to address the research question? |  |  |  |  |
|  | 5.2. Are the different components of the study effectively integrated to answer the research question? |  |  |  |  |
|  | 5.3. Are the outputs of the integration of qualitative and quantitative components adequately interpreted? |  |  |  |  |
|  | 5.4. Are divergences and inconsistencies between quantitative and qualitative results adequately addressed? |  |  |  |  |
|  | 5.5. Do the different components of the study adhere to the quality criteria of each tradition of the methods involved? |  |  |  |  |

| [2] Argyle E, Schneider J. Research based theatre in dementia knowledge transfer: views from the front line. Journal of Mental Health Training, Education & Practice. 2016;11(2):102-11. | | | | | |
| --- | --- | --- | --- | --- | --- |
|  | | | | | |
| **Category of study designs** | **Methodological quality criteria** | **Responses** | | | |
|  |  | Yes | No | Can’t tell | Comments |
| Screening questions  (for all types) | S1. Are there clear research questions? | x |  |  | *Clearly formulated as research objective:*  To examine the effectiveness of a research-based theatrical event in promoting dementia knowledge transfer with a group of front line care workers. |
|  | S2. Do the collected data allow to address the research questions? |  | x |  | *The effectiveness of an intervention should be explored with an RCT.* |
|  | *Further appraisal may not be feasible or appropriate when the answer is ‘No’ or ‘Can’t tell’ to one or both screening questions.* | | | | |
| 1. Qualitative | 1.1. Is the qualitative approach appropriate to answer the research question? | x |  |  | *Yes, the sub-goal is achievable through qualitative methods:*  These aimed to illuminate the degree to which participants had implemented their post event practice resolutions, as well as identifying potential barriers and facilitators to their achievement with these issues being explored in a semi-structured interview format. |
|  | 1.2. Are the qualitative data collection methods adequate to address the research question? | x |  |  | *See above.* |
|  | 1.3. Are the findings adequately derived from the data? | x |  |  | *Probably yes:*  From these 112 participants, 25 were randomly selected to be contacted. Initial contact was made either by e-mail or phone depending on the contact details given and short phone interviews were carried out with the eight respondents who replied to the  invitation. Again this was a fairly low response rate (32 per cent) exacerbated by the inaccessible contact details given by some respondents. Following data collection, all the pre-coded responses were entered onto a spread sheet and analysed accordingly while open ended comments and interview responses were transcribed and analysed in a multi-staged process. Elements of grounded theory were used in this process including the use of coding and analytical memos in order to identify emergent themes. |
|  | 1.4. Is the interpretation of results sufficiently substantiated by data? |  |  | x | *Remains unclear as the data interpretation was not supported with quotes.* |
|  | 1.5. Is there coherence between qualitative data sources, collection, analysis and interpretation? |  |  | x | *No statement possible due to incomplete information in the paper.* |
| 2. Quantitative randomized controlled trials | 2.1. Is randomization appropriately performed? |  |  |  |  |
|  | 2.2. Are the groups comparable at baseline? |  |  |  |  |
|  | 2.3. Are there complete outcome data? |  |  |  |  |
|  | 2.4. Are outcome assessors blinded to the intervention provided? |  |  |  |  |
|  | 2.5 Did the participants adhere to the assigned intervention? |  |  |  |  |
| 3. Quantitative non-randomized | 3.1. Are the participants representative of the target population? |  |  | x | *Unfortunately, there is a lack of detailed information on the characteristics of the participants, so that no details can be given on this.*  In order to gather respondent profiles, evaluate the event and reflect on its success in achieving its objectives, all 1,109 participants were asked to give questionnaire feedback immediately after the event. In total, 863 questionnaires were returned giving a response rate of 77 per cent.  (…)  It was found that the majority of 863 participants who returned the initial evaluation questionnaire worked with people with dementia on a regular basis (75 per cent). |
|  | 3.2. Are measurements appropriate regarding both the outcome and intervention (or exposure)? |  |  | x | *The variables to be measured were clearly defined and presumably measured accurately and are thus suitable to answer the research question. However, all questionnaires were self-developed.*  The questionnaire had been developed in the initial pilot phase of the project and in order to facilitate ease of completion and processing it was intentionally short, including only ten questions. These included pre-coded questions gathering details of participants including their employing organisation, how regularly they worked with people with dementia, how confident they were in this role and whether they had received any training in dementia care. |
|  | 3.3. Are there complete outcome data? |  | x |  | *Participants included in the intervention: n = 1109*  *Participants included in analysis: Participants who answered first questionnaire: n = 863; Participants who answered follow-up questionnaire: n = 30;* |
|  | 3.4. Are the confounders accounted for in the design and analysis? |  | x |  | *No methods for controlling confounding factors were described.* |
|  | 3.5. During the study period, is the intervention administered (or exposure occurred) as intended? |  |  | x | *No statement on this.* |
| 4. Quantitative descriptive | 4.1. Is the sampling strategy relevant to address the research question? |  |  |  |  |
|  | 4.2. Is the sample representative of the target population? |  |  |  |  |
|  | 4.3. Are the measurements appropriate? |  |  |  |  |
|  | 4.4. Is the risk of nonresponse bias low? |  |  |  |  |
|  | 4.5. Is the statistical analysis appropriate to answer the research question? |  |  |  |  |
| 5. Mixed methods | 5.1. Is there an adequate rationale for using a mixed methods design to address the research question? |  |  |  |  |
|  | 5.2. Are the different components of the study effectively integrated to answer the research question? |  |  |  |  |
|  | 5.3. Are the outputs of the integration of qualitative and quantitative components adequately interpreted? |  |  |  |  |
|  | 5.4. Are divergences and inconsistencies between quantitative and qualitative results adequately addressed? |  |  |  |  |
|  | 5.5. Do the different components of the study adhere to the quality criteria of each tradition of the methods involved? |  |  |  |  |

| [3] Baillie L, Sills E, Thomas N. Educating a health service workforce about dementia: a qualitative study. Quality in Ageing & Older Adults. 2016;17(2):119-30. | | | | | |
| --- | --- | --- | --- | --- | --- |
|  | | | | | |
| **Category of study designs** | **Methodological quality criteria** | **Responses** | | | |
|  |  | Yes | No | Can’t tell | Comments |
| Screening questions  (for all types) | S1. Are there clear research questions? | x |  |  | *Formulated as research objective:*  The study’s aim was to investigate staff perspectives of the effect of Barbara’s Story on  themselves, their colleagues and the organisation. The study was qualitative and took a social constructionist approach, with the view that people together create social phenomena (Berger and Luckman, 1966), a perspective that aligned well with the development and delivery of Barbara’s Story across the Trust. |
|  | S2. Do the collected data allow to address the research questions? | x |  |  | *The findings are presented in four themes:*  *1. Barbara’s Story as an educational device and its delivery;*  *2. effect on individual staff: emotional engagement, awareness, interactions and behaviour;*  *3. practice developments and improvements; and*  *4. organisational culture and sustainability.* |
|  | *Further appraisal may not be feasible or appropriate when the answer is ‘No’ or ‘Can’t tell’ to one or both screening questions.* | | | | |
| 1. Qualitative | 1.1. Is the qualitative approach appropriate to answer the research question? | x |  |  | “staff perspectives” |
|  | 1.2. Are the qualitative data collection methods adequate to address the research question? | x |  |  | Focus groups were used as they link individual and group interactions and subjective views are seen within a fuller social context (Burr, 1995). Focus groups also enable exploration of processes as well as outcomes (Barbour, 2007) and prompt the gathering of rich data about experiences (Plummer-D’Amato, 2008). The interactions between the participants stimulate more ideas for discussion; these data created through interactions are a fundament component of focus group methodology and can provide insights into public discourse (Kitzinger, 1994). |
|  | 1.3. Are the findings adequately derived from the data? | x |  |  | The data were analysed using the framework approach, which is a five stage process (Ritchie and Spencer, 1994): familiarization with the data; development of a thematic framework; systematic application of the thematic framework to all data; charting of coded data; and reviewing charts and searching for patterns. |
|  | 1.4. Is the interpretation of results sufficiently substantiated by data? | x |  |  | *Backed up by direct quotes* |
|  | 1.5. Is there coherence between qualitative data sources, collection, analysis and interpretation? | x |  |  |  |
| 2. Quantitative randomized controlled trials | 2.1. Is randomization appropriately performed? |  |  |  |  |
|  | 2.2. Are the groups comparable at baseline? |  |  |  |  |
|  | 2.3. Are there complete outcome data? |  |  |  |  |
|  | 2.4. Are outcome assessors blinded to the intervention provided? |  |  |  |  |
|  | 2.5 Did the participants adhere to the assigned intervention? |  |  |  |  |
| 3. Quantitative non-randomized | 3.1. Are the participants representative of the target population? |  |  |  |  |
|  | 3.2. Are measurements appropriate regarding both the outcome and intervention (or exposure)? |  |  |  |  |
|  | 3.3. Are there complete outcome data? |  |  |  |  |
|  | 3.4. Are the confounders accounted for in the design and analysis? |  |  |  |  |
|  | 3.5. During the study period, is the intervention administered (or exposure occurred) as intended? |  |  |  |  |
| 4. Quantitative descriptive | 4.1. Is the sampling strategy relevant to address the research question? |  |  |  |  |
|  | 4.2. Is the sample representative of the target population? |  |  |  |  |
|  | 4.3. Are the measurements appropriate? |  |  |  |  |
|  | 4.4. Is the risk of nonresponse bias low? |  |  |  |  |
|  | 4.5. Is the statistical analysis appropriate to answer the research question? |  |  |  |  |
| 5. Mixed methods | 5.1. Is there an adequate rationale for using a mixed methods design to address the research question? |  |  |  |  |
|  | 5.2. Are the different components of the study effectively integrated to answer the research question? |  |  |  |  |
|  | 5.3. Are the outputs of the integration of qualitative and quantitative components adequately interpreted? |  |  |  |  |
|  | 5.4. Are divergences and inconsistencies between quantitative and qualitative results adequately addressed? |  |  |  |  |
|  | 5.5. Do the different components of the study adhere to the quality criteria of each tradition of the methods involved? |  |  |  |  |

| [4] Bard JT, Chung HK, Shaia JK, Wellman LL, Elzie CA. Increased medical student understanding of dementia through virtual embodiment. Gerontology & Geriatrics Education. 2022;44(2):211-22. | | | | | |
| --- | --- | --- | --- | --- | --- |
|  | | | | | |
| **Category of study designs** | **Methodological quality criteria** | **Responses** | | | |
|  |  | Yes | No | Can’t tell | Comments |
| Screening questions  (for all types) | S1. Are there clear research questions? | x |  |  | *Formulated as research objective:*  To determine the feasibility and efficacy of virtual reality (VR) as a learning tool for preclinical medical students to generate understanding and empathy for individuals living with dementia and their families |
|  | S2. Do the collected data allow to address the research questions? | x |  |  | *Taken all together, the data presented reflect the objective and thus partly answer the research objectives.* |
|  | *Further appraisal may not be feasible or appropriate when the answer is ‘No’ or ‘Can’t tell’ to one or both screening questions.* | | | | |
| 1. Qualitative | 1.1. Is the qualitative approach appropriate to answer the research question? |  |  |  |  |
|  | 1.2. Are the qualitative data collection methods adequate to address the research question? |  |  |  |  |
|  | 1.3. Are the findings adequately derived from the data? |  |  |  |  |
|  | 1.4. Is the interpretation of results sufficiently substantiated by data? |  |  |  |  |
|  | 1.5. Is there coherence between qualitative data sources, collection, analysis and interpretation? |  |  |  |  |
| 2. Quantitative randomized controlled trials | 2.1. Is randomization appropriately performed? |  |  |  |  |
|  | 2.2. Are the groups comparable at baseline? |  |  |  |  |
|  | 2.3. Are there complete outcome data? |  |  |  |  |
|  | 2.4. Are outcome assessors blinded to the intervention provided? |  |  |  |  |
|  | 2.5 Did the participants adhere to the assigned intervention? |  |  |  |  |
| 3. Quantitative non-randomized | 3.1. Are the participants representative of the target population? |  |  | x | *As the target group of the intervention is not described, it remains unclear whether and to what extent the included participants represent the target group.* |
|  | 3.2. Are measurements appropriate regarding both the outcome and intervention (or exposure)? |  | x |  | *The variables to be measured were clearly defined and thus suitable for answering the research question: Pre-Survey: personal experiences with dementia; Post-Survey: Students thoughts about the VR experience; Both: understanding of the feelings of both caregivers and persons with dementia.*  *Some of the instruments used were self-constructed, which is to be assessed negatively. Furthermore, feasibility is missing and efficacy could be better evaluated by a different design.* |
|  | 3.3. Are there complete outcome data? | x |  |  | *Participants of the intervention: n = 150*  *Participants included in analysis: n = 150 (pre-survey: n = 149; post-survey: n = 150)* |
|  | 3.4. Are the confounders accounted for in the design and analysis? |  | x |  | *No methods for controlling confounding factors were described.* |
|  | 3.5. During the study period, is the intervention administered (or exposure occurred) as intended? |  |  | x | *No information.* |
| 4. Quantitative descriptive | 4.1. Is the sampling strategy relevant to address the research question? |  |  |  |  |
|  | 4.2. Is the sample representative of the target population? |  |  |  |  |
|  | 4.3. Are the measurements appropriate? |  |  |  |  |
|  | 4.4. Is the risk of nonresponse bias low? |  |  |  |  |
|  | 4.5. Is the statistical analysis appropriate to answer the research question? |  |  |  |  |
| 5. Mixed methods | 5.1. Is there an adequate rationale for using a mixed methods design to address the research question? |  |  |  |  |
|  | 5.2. Are the different components of the study effectively integrated to answer the research question? |  |  |  |  |
|  | 5.3. Are the outputs of the integration of qualitative and quantitative components adequately interpreted? |  |  |  |  |
|  | 5.4. Are divergences and inconsistencies between quantitative and qualitative results adequately addressed? |  |  |  |  |
|  | 5.5. Do the different components of the study adhere to the quality criteria of each tradition of the methods involved? |  |  |  |  |

| [5] Board M, Murphy J, Mitchell R, Phipps L, Fossey I. The Lived Experience of Dementia. A new resource for the health and social care workforce. 2019. | | | | | |
| --- | --- | --- | --- | --- | --- |
|  | | | | | |
| **Category of study designs** | **Methodological quality criteria** | **Responses** | | | |
|  |  | Yes | No | Can’t tell | Comments |
| Screening questions  (for all types) | S1. Are there clear research questions? | x |  |  | *Clearly formulated as research objective:*   - to evaluate via questionnaire participants perspective on the value of the app in understanding the lived experience of dementia - to conduct focus groups with the participant after a period of time in practice to see if viewing the app influenced the care they offered to patients, and in a humanised way. |
|  | S2. Do the collected data allow to address the research questions? | x |  |  | *Certainly, the data on understanding the lived experience of dementia could have been collected by a qualitative method, but a quantitative method should be seen as equivalent method to be used.* |
|  | *Further appraisal may not be feasible or appropriate when the answer is ‘No’ or ‘Can’t tell’ to one or both screening questions.* | | | | |
| 1. Qualitative | 1.1. Is the qualitative approach appropriate to answer the research question? | x |  |  | *Yes, the sub-goal is achievable through qualitative methods:*  to conduct focus groups with the participant after a period of time in practice to see if viewing the app influenced the care they offered to patients, and in a humanised way |
|  | 1.2. Are the qualitative data collection methods adequate to address the research question? | x |  |  | *See above.* |
|  | 1.3. Are the findings adequately derived from the data? |  |  | x | *Lack of data.* |
|  | 1.4. Is the interpretation of results sufficiently substantiated by data? |  |  | x | *Lack of data.* |
|  | 1.5. Is there coherence between qualitative data sources, collection, analysis and interpretation? |  |  | x | *Lack of data.* |
| 2. Quantitative randomized controlled trials | 2.1. Is randomization appropriately performed? |  |  |  |  |
|  | 2.2. Are the groups comparable at baseline? |  |  |  |  |
|  | 2.3. Are there complete outcome data? |  |  |  |  |
|  | 2.4. Are outcome assessors blinded to the intervention provided? |  |  |  |  |
|  | 2.5 Did the participants adhere to the assigned intervention? |  |  |  |  |
| 3. Quantitative non-randomized | 3.1. Are the participants representative of the target population? |  |  |  |  |
|  | 3.2. Are measurements appropriate regarding both the outcome and intervention (or exposure)? |  |  |  |  |
|  | 3.3. Are there complete outcome data? |  |  |  |  |
|  | 3.4. Are the confounders accounted for in the design and analysis? |  |  |  |  |
|  | 3.5. During the study period, is the intervention administered (or exposure occurred) as intended? |  |  |  |  |
| 4. Quantitative descriptive | 4.1. Is the sampling strategy relevant to address the research question? |  |  | x | *Lack of data.* |
|  | 4.2. Is the sample representative of the target population? |  |  | x | *Lack of data.* |
|  | 4.3. Are the measurements appropriate? |  |  | x | *Lack of data.* |
|  | 4.4. Is the risk of nonresponse bias low? |  |  | x | *Lack of data.* |
|  | 4.5. Is the statistical analysis appropriate to answer the research question? |  |  | x | *Lack of data.* |
| 5. Mixed methods | 5.1. Is there an adequate rationale for using a mixed methods design to address the research question? |  |  |  |  |
|  | 5.2. Are the different components of the study effectively integrated to answer the research question? |  |  |  |  |
|  | 5.3. Are the outputs of the integration of qualitative and quantitative components adequately interpreted? |  |  |  |  |
|  | 5.4. Are divergences and inconsistencies between quantitative and qualitative results adequately addressed? |  |  |  |  |
|  | 5.5. Do the different components of the study adhere to the quality criteria of each tradition of the methods involved? |  |  |  |  |

| [6] Campbell D, Lugger S, Sigler GS, Turkelson C. Increasing awareness, sensitivity, and empathy for Alzheimer's dementia patients using simulation. Nurse Education Today. 2021;98:e104764. | | | | | |
| --- | --- | --- | --- | --- | --- |
|  | | | | | |
| **Category of study designs** | **Methodological quality criteria** | **Responses** | | | |
|  |  | Yes | No | Can’t tell | Comments |
| Screening questions  (for all types) | S1. Are there clear research questions? | x |  |  | *Formulated as research objective:*  To evaluate perceptions of awareness, knowledge, and sensitivity of nursing students towards patients with AD before and after participation in a simulated virtual dementia experience. |
|  | S2. Do the collected data allow to address the research questions? | x |  |  | Surveys were administered prior and immediately after attendance at the VDT® sessions via validated instruments for capturing awareness, knowledge and sensitivity of future nurses concerning AD patients before and after participation in a simulated virtual reality dementia experience. |
|  | *Further appraisal may not be feasible or appropriate when the answer is ‘No’ or ‘Can’t tell’ to one or both screening questions.* | | | | |
| 1. Qualitative | 1.1. Is the qualitative approach appropriate to answer the research question? |  |  |  |  |
|  | 1.2. Are the qualitative data collection methods adequate to address the research question? |  |  |  |  |
|  | 1.3. Are the findings adequately derived from the data? |  |  |  |  |
|  | 1.4. Is the interpretation of results sufficiently substantiated by data? |  |  |  |  |
|  | 1.5. Is there coherence between qualitative data sources, collection, analysis and interpretation? |  |  |  |  |
| 2. Quantitative randomized controlled trials | 2.1. Is randomization appropriately performed? |  |  |  |  |
|  | 2.2. Are the groups comparable at baseline? |  |  |  |  |
|  | 2.3. Are there complete outcome data? |  |  |  |  |
|  | 2.4. Are outcome assessors blinded to the intervention provided? |  |  |  |  |
|  | 2.5 Did the participants adhere to the assigned intervention? |  |  |  |  |
| 3. Quantitative non-randomized | 3.1. Are the participants representative of the target population? |  |  | x | A total of 163 traditional and accelerated BSN nursing students participated in the VDT® experience. The participants included students in the traditional bachelor of science (BSN) in nursing program who were enrolled in fundamentals (n = 48), medical surgical I traditional track (n = 41), mental health (n = 34) or accelerated track medical surgical I course (n = 40).  *No statement can be made about this, as information in the manuscript is not (clearly) formulated.* |
|  | 3.2. Are measurements appropriate regarding both the outcome and intervention (or exposure)? | x |  |  | *The variables to be measured were clearly defined and thus suitable for answering the research question:*  The primary goal of this project was to evaluate perceptions of awareness, knowledge and sensitivity of future nurses concerning AD patients before and after participation in a simulated virtual reality dementia experience. Surveys were administered prior and immediately after attendance at the VDT® sessions.  *One of the instruments used was self-constructed, which is to be assessed negatively.* |
|  | 3.3. Are there complete outcome data? |  | x |  | *Participants included in the intervention: n = 163*  *Participants included in analysis: n = 70* |
|  | 3.4. Are the confounders accounted for in the design and analysis? |  | x |  | *No methods for controlling confounding factors were described.* |
|  | 3.5. During the study period, is the intervention administered (or exposure occurred) as intended? | x |  |  | *There are no changes in exposure status from the intended intervention.* |
| 4. Quantitative descriptive | 4.1. Is the sampling strategy relevant to address the research question? |  |  |  |  |
|  | 4.2. Is the sample representative of the target population? |  |  |  |  |
|  | 4.3. Are the measurements appropriate? |  |  |  |  |
|  | 4.4. Is the risk of nonresponse bias low? |  |  |  |  |
|  | 4.5. Is the statistical analysis appropriate to answer the research question? |  |  |  |  |
| 5. Mixed methods | 5.1. Is there an adequate rationale for using a mixed methods design to address the research question? |  |  |  |  |
|  | 5.2. Are the different components of the study effectively integrated to answer the research question? |  |  |  |  |
|  | 5.3. Are the outputs of the integration of qualitative and quantitative components adequately interpreted? |  |  |  |  |
|  | 5.4. Are divergences and inconsistencies between quantitative and qualitative results adequately addressed? |  |  |  |  |
|  | 5.5. Do the different components of the study adhere to the quality criteria of each tradition of the methods involved? |  |  |  |  |
| [7] de Abreu ID, Hinojosa-Lindsey M, Asghar-Ali AA. A Simulation Exercise to Raise Learners' Awareness of the Physical and Cognitive Changes in Older Adults. Academic Psychiatry. 2017;41(5):684-7. | | | | | |
|  | | | | | |
| **Category of study designs** | **Methodological quality criteria** | **Responses** | | | |
|  |  | Yes | No | Can’t tell | Comments |
| Screening questions  (for all types) | S1. Are there clear research questions? | x |  |  | *Formulated as research objectives:*   - To examine the effects of a simulation exercise on participants’ attitudes towards older individuals with cognitive impairment; - Hypothesis testing: learners would have a more positive attitude towards physical and cognitive changes in older individuals after completing the simulation exercise |
|  | S2. Do the collected data allow to address the research questions? | x |  |  | Pretest and posttest survey data collected from students at Baylor College of Medicine were analyzed to examine the effects of a simulation exercise on participants’ attitudes towards older individuals with cognitive impairment. |
|  | *Further appraisal may not be feasible or appropriate when the answer is ‘No’ or ‘Can’t tell’ to one or both screening questions.* | | | | |
| 1. Qualitative | 1.1. Is the qualitative approach appropriate to answer the research question? |  |  |  |  |
|  | 1.2. Are the qualitative data collection methods adequate to address the research question? |  |  |  |  |
|  | 1.3. Are the findings adequately derived from the data? |  |  |  |  |
|  | 1.4. Is the interpretation of results sufficiently substantiated by data? |  |  |  |  |
|  | 1.5. Is there coherence between qualitative data sources, collection, analysis and interpretation? |  |  |  |  |
| 2. Quantitative randomized controlled trials | 2.1. Is randomization appropriately performed? |  |  |  |  |
|  | 2.2. Are the groups comparable at baseline? |  |  |  |  |
|  | 2.3. Are there complete outcome data? |  |  |  |  |
|  | 2.4. Are outcome assessors blinded to the intervention provided? |  |  |  |  |
|  | 2.5 Did the participants adhere to the assigned intervention? |  |  |  |  |
| 3. Quantitative non-randomized | 3.1. Are the participants representative of the target population? |  | x |  | The participants were a convenience sample of Baylor College of Medicine (BCM) learners in their core psychiatry rotation while at the Michael E. DeBakey VA Medical Center (MEDVAMC). The participants who completed the pretest, simulation exercise, and posttest surveys came from four groups of learners: medical students, physician assistant students, pharmacy interns, and psychiatry residents at BCM. All learners were invited to participate (N = 100) via email at the beginning of their rotation. From those who were invited, 51 learners chose to participate, 49 learners completed informed consent, and 48 learners completed both the pre- and posttests. A time conflict with clinical duties was the most common reason for not participating.  *Since only about half of the originally intended target group participated, it is not possible to speak of representativeness.* |
|  | 3.2. Are measurements appropriate regarding both the outcome and intervention (or exposure)? | x |  |  | *The variables to be measured were clearly defined and thus suitable for answering the research question; good reliability and validity of the instrument used:*  To assess learners’ attitudes towards individuals with cognitive impairment, the ADQ was administered just before, and immediately after, the simulation exercise. |
|  | 3.3. Are there complete outcome data? | x |  |  | A total of 49 learners participated in this study; 48 completed both the pre- and posttests. |
|  | 3.4. Are the confounders accounted for in the design and analysis? |  | x |  | *No methods for controlling confounding factors were described.* |
|  | 3.5. During the study period, is the intervention administered (or exposure occurred) as intended? | x |  |  | *There are no changes in exposure status from the intended intervention.* |
| 4. Quantitative descriptive | 4.1. Is the sampling strategy relevant to address the research question? |  |  |  |  |
|  | 4.2. Is the sample representative of the target population? |  |  |  |  |
|  | 4.3. Are the measurements appropriate? |  |  |  |  |
|  | 4.4. Is the risk of nonresponse bias low? |  |  |  |  |
|  | 4.5. Is the statistical analysis appropriate to answer the research question? |  |  |  |  |
| 5. Mixed methods | 5.1. Is there an adequate rationale for using a mixed methods design to address the research question? |  |  |  |  |
|  | 5.2. Are the different components of the study effectively integrated to answer the research question? |  |  |  |  |
|  | 5.3. Are the outputs of the integration of qualitative and quantitative components adequately interpreted? |  |  |  |  |
|  | 5.4. Are divergences and inconsistencies between quantitative and qualitative results adequately addressed? |  |  |  |  |
|  | 5.5. Do the different components of the study adhere to the quality criteria of each tradition of the methods involved? |  |  |  |  |

| [8] Dupuis SL, Mitchell GJ, Jonas-Simpson CM, Whyte CP, Gillies JL, Carson JD. Igniting Transformative Change in Dementia Care Through Research-based Drama. Gerontologist. 2015;56(6):1042-52. | | | | | |
| --- | --- | --- | --- | --- | --- |
|  | | | | | |
| **Category of study designs** | **Methodological quality criteria** | **Responses** | | | |
|  |  | Yes | No | Can’t tell | Comments |
| Screening questions  (for all types) | S1. Are there clear research questions? | x |  |  | *Formulation as research objective:*  This article explores how audience members experienced I’m Still Here over a 1-year period and how participants described shorter-term (6 weeks) and longer-term, sustained (12 months) change. The longitudinal data provide a deeper understanding of the experiential process of participants’ personal transformations.  Underpinned by phenomenology |
|  | S2. Do the collected data allow to address the research questions? | x |  |  | The phenomenological shifts reflected in the longitudinal data suggest a process of engagement with research-based drama that involves four themes: bearing witness to suffering evokes compassion; expanding with new awareness and understanding;  finding comfort, confidence, and courage to change; and envisioning and enacting new possibilities. |
|  | *Further appraisal may not be feasible or appropriate when the answer is ‘No’ or ‘Can’t tell’ to one or both screening questions.* | | | | |
| 1. Qualitative | 1.1. Is the qualitative approach appropriate to answer the research question? | x |  |  | Our research was informed by hermeneutic phenomenology (van Manen, 1990) and focused on persons’ experiences of their lifeworld contexts. Phenomenology illuminates how the world is made meaningful and how human beings apprehend and act upon various objects of lived experience. Phenomenology is well suited for studying both illness and health care and engagement with the arts because it aims to understand how “experiential processes proceed and what is experienced through them” (Wertz, Nosek, McNiesh, & Marlow, 2011, p. 126). |
|  | 1.2. Are the qualitative data collection methods adequate to address the research question? | x |  |  | Initially, participants attended two separate focus groups (i.e., one before and one directly following the play). Audio-recorded telephone interviews were then conducted  with the participants at 6-week and 12-month intervals postperformance to examine any shorter- and longer-term impacts of the research-based drama. |
|  | 1.3. Are the findings adequately derived from the data? | x |  |  | Both the focus group and interview data were transcribed verbatim and analyzed following guidelines described by van Manen (1990) and Giorgi (2009): reading for a sense of the whole; identifying, naming, and differentiating expressions of the phenomenon (meaning units); reflecting on the significance of meaning units; identifying common and divergent expressions of the experience; identifying, clarifying,  and verifying theme clusters that reflected the essence of the whole experience; and writing a synthetic or thick description of the experience using the theme clusters and  quotes from the data. |
|  | 1.4. Is the interpretation of results sufficiently substantiated by data? | x |  |  | *Backed up by quotes and further examples.* |
|  | 1.5. Is there coherence between qualitative data sources, collection, analysis and interpretation? | x |  |  | *The intended research goals are reflected in the identified themes as well as in the data interpretation.* |
| 2. Quantitative randomized controlled trials | 2.1. Is randomization appropriately performed? |  |  |  |  |
|  | 2.2. Are the groups comparable at baseline? |  |  |  |  |
|  | 2.3. Are there complete outcome data? |  |  |  |  |
|  | 2.4. Are outcome assessors blinded to the intervention provided? |  |  |  |  |
|  | 2.5 Did the participants adhere to the assigned intervention? |  |  |  |  |
| 3. Quantitative non-randomized | 3.1. Are the participants representative of the target population? |  |  |  |  |
|  | 3.2. Are measurements appropriate regarding both the outcome and intervention (or exposure)? |  |  |  |  |
|  | 3.3. Are there complete outcome data? |  |  |  |  |
|  | 3.4. Are the confounders accounted for in the design and analysis? |  |  |  |  |
|  | 3.5. During the study period, is the intervention administered (or exposure occurred) as intended? |  |  |  |  |
| 4. Quantitative descriptive | 4.1. Is the sampling strategy relevant to address the research question? |  |  |  |  |
|  | 4.2. Is the sample representative of the target population? |  |  |  |  |
|  | 4.3. Are the measurements appropriate? |  |  |  |  |
|  | 4.4. Is the risk of nonresponse bias low? |  |  |  |  |
|  | 4.5. Is the statistical analysis appropriate to answer the research question? |  |  |  |  |
| 5. Mixed methods | 5.1. Is there an adequate rationale for using a mixed methods design to address the research question? |  |  |  |  |
|  | 5.2. Are the different components of the study effectively integrated to answer the research question? |  |  |  |  |
|  | 5.3. Are the outputs of the integration of qualitative and quantitative components adequately interpreted? |  |  |  |  |
|  | 5.4. Are divergences and inconsistencies between quantitative and qualitative results adequately addressed? |  |  |  |  |
|  | 5.5. Do the different components of the study adhere to the quality criteria of each tradition of the methods involved? |  |  |  |  |

| [9] Garrod L, Fossey J, Henshall C, Williamson S, Coates A, Green H. Evaluating dementia training for healthcare staff. The Journal of Mental Health Training, Education and Practice. 2019;14(4):277-88. | | | | | |
| --- | --- | --- | --- | --- | --- |
|  | | | | | |
| **Category of study designs** | **Methodological quality criteria** | **Responses** | | | |
|  |  | Yes | No | Can’t tell | Comments |
| Screening questions  (for all types) | S1. Are there clear research questions? | x |  |  | *Formulated as research objective:*  To report on a service evaluation of a competency-based dementia training programme for clinicians to establish its value in improving their knowledge and confidence of dementia care and to explore any resulting changes to practice. |
|  | S2. Do the collected data allow to address the research questions? | x |  |  | *Data collection included focus groups, and quantitative questionnaires to cover all research objectives* |
|  | *Further appraisal may not be feasible or appropriate when the answer is ‘No’ or ‘Can’t tell’ to one or both screening questions.* | | | | |
| 1. Qualitative | 1.1. Is the qualitative approach appropriate to answer the research question? | x |  |  | *Yes, the sub-objective (for the qualitative approach) can best be captured through a qualitative research approach.*  Skills and behavior: The effect of the training on practice was evaluated qualitatively in two FGDs, one with staff six to nine months after they had attended the training and the other with managers of staff who had attended the training six to nine months previously. |
|  | 1.2. Are the qualitative data collection methods adequate to address the research question? | x |  |  | *See above. Individual interviews would also have been possible, but this way the discussion could be more stimulating.* |
|  | 1.3. Are the findings adequately derived from the data? |  |  |  | *No evidence that findings were not adequately derived:*  Level 3 analysis – skills and behaviour. Following a thematic analysis approach (Braun and Clarke, 2006), the two members of the research team (CH, HG) who had facilitated the FGDs double coded the transcripts. Emerging themes from the transcripts were collated by both researchers who then met up to discuss and sense check their findings with one another. Following this, key themes were agreed and developed further and were then shared with the other members of the research team. |
|  | 1.4. Is the interpretation of results sufficiently substantiated by data? | x |  |  | *Interpretations are underpinned by quotes and further examples.* |
|  | 1.5. Is there coherence between qualitative data sources, collection, analysis and interpretation? | x |  |  |  |
| 2. Quantitative randomized controlled trials | 2.1. Is randomization appropriately performed? |  |  |  |  |
|  | 2.2. Are the groups comparable at baseline? |  |  |  |  |
|  | 2.3. Are there complete outcome data? |  |  |  |  |
|  | 2.4. Are outcome assessors blinded to the intervention provided? |  |  |  |  |
|  | 2.5 Did the participants adhere to the assigned intervention? |  |  |  |  |
| 3. Quantitative non-randomized | 3.1. Are the participants representative of the target population? |  | x |  | The Tier 2 training programme was run 11 times between May 2017 and the end of December 2017, with 18 places available per cohort. Attendance on the training was voluntary and places were advertised through the Learning and Development portal. Managers were encouraged to support their team members to attend. All staff attending the training were invited to participate in the evaluation. The primary target audience was Healthcare Assistants (HCAs), Registered Nurses and Allied Health Professionals (AHPs) all of whom were working in settings where they were likely to have contact with patients living with dementia, such as community hospitals, older people’s mental health wards and community teams. While the training was open to other staff, such as junior doctors, it was not specifically targeted towards them.  In total, 162 participants undertook the Tier 2 dementia training: 61 were nurses  (37.65 per cent), 50 were HCAs (30.86 per cent), 17 were AHPs (10.49 per cent), 15 were in other roles (9.25 per cent) and 9 were managers (5.55 per cent). Data regarding role were missing for 10 (6.17 per cent) individuals.  *It is not clear from the data to what extent the intended group was reached.* |
|  | 3.2. Are measurements appropriate regarding both the outcome and intervention (or exposure)? | x |  |  | *The variables to be measured were clearly defined and suitable for answering the research question; good reliability and validity of the instrument used. Only the response to brief vignettes was adapted.*  Level 1 analysis – reaction to training. The quantitative responses were analysed by calculating mean scores and the qualitative responses were analysed by categorising comments into themes.  Level 2 analysis – knowledge. Scores from each item on the ADKS, CODES and Vignettes were summed and compared across T1 and T2 using Wilcoxon signed-rank test to identify any significant changes in pre-and post-test knowledge and confidence. |
|  | 3.3. Are there complete outcome data? | x |  |  | *Participants of the intervention: n = 162*  *Participants included in analysis: Number of completed measures pre-test: n = 150-159; Number of completed measured post-test: n = 146-151* |
|  | 3.4. Are the confounders accounted for in the design and analysis? |  | x |  | *No methods for controlling confounding factors were described.* |
|  | 3.5. During the study period, is the intervention administered (or exposure occurred) as intended? | x |  |  | *There are no changes in exposure status from the intended intervention.* |
| 4. Quantitative descriptive | 4.1. Is the sampling strategy relevant to address the research question? |  |  |  |  |
|  | 4.2. Is the sample representative of the target population? |  |  |  |  |
|  | 4.3. Are the measurements appropriate? |  |  |  |  |
|  | 4.4. Is the risk of nonresponse bias low? |  |  |  |  |
|  | 4.5. Is the statistical analysis appropriate to answer the research question? |  |  |  |  |
| 5. Mixed methods | 5.1. Is there an adequate rationale for using a mixed methods design to address the research question? |  | x |  | *Due to the complexity of the research objective, which consists of many components, the application of a mixed methods design makes sense.*  *However, the rationale is not explained or described in the report.* |
|  | 5.2. Are the different components of the study effectively integrated to answer the research question? |  | x |  | *No, results were only presented side by side and not integrated.* |
|  | 5.3. Are the outputs of the integration of qualitative and quantitative components adequately interpreted? |  | x |  | *As no data integration took place, no meta-inferences were derived.* |
|  | 5.4. Are divergences and inconsistencies between quantitative and qualitative results adequately addressed? |  |  | x | *No statement possible, as data was not integrated or compared.* |
|  | 5.5. Do the different components of the study adhere to the quality criteria of each tradition of the methods involved? |  | x |  | *Presence of medium to low quality, as partial studies are of medium quality.* |

| [10] Gilmartin-Thomas JFM, McNeil J, Powell A, Malone DT, Wolfe R, Larson IC, et al. Impact of a Virtual Dementia Experience on Medical and Pharmacy Students' Knowledge and Attitudes Toward People with Dementia: A Controlled Study. J Alzheimers Dis. 2018;62(2):867-76. | | | | | |
| --- | --- | --- | --- | --- | --- |
|  | | | | | |
| **Category of study designs** | **Methodological quality criteria** | **Responses** | | | |
|  |  | Yes | No | Can’t tell | Comments |
| Screening questions  (for all types) | S1. Are there clear research questions? | x |  |  | *Formulated as research objectives:*  This study quantitatively evaluated the impact of a virtual dementia experience on medical and pharmacy students’ knowledge and attitudes toward people with dementia. |
|  | S2. Do the collected data allow to address the research questions? | x |  |  | A two-group, non-randomized controlled study for medical and pharmacy students was conducted from September to October 2016. |
|  | *Further appraisal may not be feasible or appropriate when the answer is ‘No’ or ‘Can’t tell’ to one or both screening questions.* | | | | |
| 1. Qualitative | 1.1. Is the qualitative approach appropriate to answer the research question? |  |  |  |  |
|  | 1.2. Are the qualitative data collection methods adequate to address the research question? |  |  |  |  |
|  | 1.3. Are the findings adequately derived from the data? |  |  |  |  |
|  | 1.4. Is the interpretation of results sufficiently substantiated by data? |  |  |  |  |
|  | 1.5. Is there coherence between qualitative data sources, collection, analysis and interpretation? |  |  |  |  |
| 2. Quantitative randomized controlled trials | 2.1. Is randomization appropriately performed? |  |  |  |  |
|  | 2.2. Are the groups comparable at baseline? |  |  |  |  |
|  | 2.3. Are there complete outcome data? |  |  |  |  |
|  | 2.4. Are outcome assessors blinded to the intervention provided? |  |  |  |  |
|  | 2.5 Did the participants adhere to the assigned intervention? |  |  |  |  |
| 3. Quantitative non-randomized | 3.1. Are the participants representative of the target population? | x |  |  | The participants were third year (of a five year) Bachelor of Medicine and Bachelor of Surgery (n = 69) and fourth year (of a four year) Bachelor of Pharmacy students (n = 229). The intervention was offered to students in these year levels because it was considered complementary to their clinical placements.  Reasons why pharmacy students did not undertake the intervention, despite expressing an interest, included: not being available when the intervention session was scheduled, no longer interested in undertaking the intervention, being unwell, not responding to email contact, and incorrect contact details provided to the research team.  *Out of 289 eligible participants, a total of 278 participants were included in the analysis.*  *Inclusion and exclusion criteria were clearly described, and the reasons for drop-out are also available and comprehensible.* *Considering the low drop-out rate, this item is to be rated as positive.* |
|  | 3.2. Are measurements appropriate regarding both the outcome and intervention (or exposure)? | x |  |  | *The variable to be measured as clearly defined and thus suitable for answering the research question; good reliability and validity of the instrument used:*  The impact of the intervention was evaluated using the 20-item Dementia Attitudes Scale (DAS) [11]. |
|  | 3.3. Are there complete outcome data? | x |  |  | *Participants included in the intervention: intervention group: n = 83; control group: n = 215*  *Participants included in analysis: A total of 278 students (n = 64 medical, n = 214 pharmacy) were analysed (n = 80 intervention, n = 198 control)* |
|  | 3.4. Are the confounders accounted for in the design and analysis? | x |  |  | *A suitable method to control for confounding factors was applied through matching.* |
|  | 3.5. During the study period, is the intervention administered (or exposure occurred) as intended? | x |  |  | *There are no changes in exposure status from the intended intervention.* |
| 4. Quantitative descriptive | 4.1. Is the sampling strategy relevant to address the research question? |  |  |  |  |
|  | 4.2. Is the sample representative of the target population? |  |  |  |  |
|  | 4.3. Are the measurements appropriate? |  |  |  |  |
|  | 4.4. Is the risk of nonresponse bias low? |  |  |  |  |
|  | 4.5. Is the statistical analysis appropriate to answer the research question? |  |  |  |  |
| 5. Mixed methods | 5.1. Is there an adequate rationale for using a mixed methods design to address the research question? |  |  |  |  |
|  | 5.2. Are the different components of the study effectively integrated to answer the research question? |  |  |  |  |
|  | 5.3. Are the outputs of the integration of qualitative and quantitative components adequately interpreted? |  |  |  |  |
|  | 5.4. Are divergences and inconsistencies between quantitative and qualitative results adequately addressed? |  |  |  |  |
|  | 5.5. Do the different components of the study adhere to the quality criteria of each tradition of the methods involved? |  |  |  |  |

| [11] Gilmartin-Thomas JFM, McNeil J, Powell A, Malone DT, Larson IC, O'Reilly CL, et al. Qualitative evaluation of how a virtual dementia experience impacts medical and pharmacy students' self-reported knowledge and attitudes towards people with dementia. Dementia (14713012). 2018;19(2):205-20. | | | | | |
| --- | --- | --- | --- | --- | --- |
|  | | | | | |
| **Category of study designs** | **Methodological quality criteria** | **Responses** | | | |
|  |  | Yes | No | Can’t tell | Comments |
| Screening questions  (for all types) | S1. Are there clear research questions? | x |  |  | *Formulated as research objective:*  To evaluate the impact of a novel virtual dementia experience, which used virtual reality to allow healthcare students to experience what it is like to live with dementia, on medical and pharmacy students’ self-reported knowledge and attitudes towards people with dementia. |
|  | S2. Do the collected data allow to address the research questions? | x |  |  | *Certainly, the data on knowledge and attitude could have been collected by a quantitative method, but a qualitative method should be seen as equivalent.* |
|  | *Further appraisal may not be feasible or appropriate when the answer is ‘No’ or ‘Can’t tell’ to one or both screening questions.* | | | | |
| 1. Qualitative | 1.1. Is the qualitative approach appropriate to answer the research question? | x |  |  | This study qualitatively evaluated the impact of a novel virtual dementia experience,  which used virtual reality to allow healthcare students to experience what it is like to live  with dementia, on medical and pharmacy students’ self-reported knowledge and attitudes towards people with dementia.  (…) Using the question guide, students were asked about their perception of the usefulness of the experience and how it could be improved. Additionally, students were asked to use their learnings from the experience to share their perception of whether medical environments/medical practitioners or pharmacies/pharmacists are currently dementia friendly (and why/why not) and suggestions to address this.  *Certainly, the data on knowledge and attitude could have been collected by a quantitative method, but a qualitative method should be seen as equivalent.* |
|  | 1.2. Are the qualitative data collection methods adequate to address the research question? | x |  |  | An open-ended, semi-structured question guide (McNeill & Chapman, 2005) was developed in conjunction with the research team to allow participants to raise new ideas or issues that they believed were important, which focus group facilitators could further explore by asking additional questions (Smith, 2002). The question guide was assessed for face and content validity among members of the research team prior to use. Using the question guide, students were asked about their perception of the usefulness of the experience and how it could be improved. Additionally, students were asked to use their learnings from the experience to share their perception of whether medical environments/medical practitioners or pharmacies/pharmacists are currently dementia friendly (and why/why not) and suggestions to address this. |
|  | 1.3. Are the findings adequately derived from the data? | x |  |  | The principal investigator analysed data using a thematic approach (Pope, Ziebland, &  Mays, 2000). Familiarisation with the raw data involved repeatedly listening to the  audio-recorded focus group discussions to give a sense of the approximate number of  unique themes that were emerging from the data and the amount of data that supported each theme. Following this, emerging themes were identified, defined, and named. An initial coding frame was formed by merging similar or dividing distinct themes to form key themes. Data (individual student quotes) were then indexed to the coding frame to elaborate on and provide evidence to support key themes. Finally, key themes were reviewed to ensure there were enough supporting data, and key themes were discussed with the research team to ensure the validity and credibility of data analysis and to clarify discrepancies.  *There is no evidence to suggest otherwise.* |
|  | 1.4. Is the interpretation of results sufficiently substantiated by data? | x |  |  | *The data interpretation was underpinned by quotes that correctly describe the respective topics.* |
|  | 1.5. Is there coherence between qualitative data sources, collection, analysis and interpretation? | x |  |  | *The intended research goals are reflected in the identified themes as well as in the data interpretation.* |
| 2. Quantitative randomized controlled trials | 2.1. Is randomization appropriately performed? |  |  |  |  |
|  | 2.2. Are the groups comparable at baseline? |  |  |  |  |
|  | 2.3. Are there complete outcome data? |  |  |  |  |
|  | 2.4. Are outcome assessors blinded to the intervention provided? |  |  |  |  |
|  | 2.5 Did the participants adhere to the assigned intervention? |  |  |  |  |
| 3. Quantitative non-randomized | 3.1. Are the participants representative of the target population? |  |  |  |  |
|  | 3.2. Are measurements appropriate regarding both the outcome and intervention (or exposure)? |  |  |  |  |
|  | 3.3. Are there complete outcome data? |  |  |  |  |
|  | 3.4. Are the confounders accounted for in the design and analysis? |  |  |  |  |
|  | 3.5. During the study period, is the intervention administered (or exposure occurred) as intended? |  |  |  |  |
| 4. Quantitative descriptive | 4.1. Is the sampling strategy relevant to address the research question? |  |  |  |  |
|  | 4.2. Is the sample representative of the target population? |  |  |  |  |
|  | 4.3. Are the measurements appropriate? |  |  |  |  |
|  | 4.4. Is the risk of nonresponse bias low? |  |  |  |  |
|  | 4.5. Is the statistical analysis appropriate to answer the research question? |  |  |  |  |
| 5. Mixed methods | 5.1. Is there an adequate rationale for using a mixed methods design to address the research question? |  |  |  |  |
|  | 5.2. Are the different components of the study effectively integrated to answer the research question? |  |  |  |  |
|  | 5.3. Are the outputs of the integration of qualitative and quantitative components adequately interpreted? |  |  |  |  |
|  | 5.4. Are divergences and inconsistencies between quantitative and qualitative results adequately addressed? |  |  |  |  |
|  | 5.5. Do the different components of the study adhere to the quality criteria of each tradition of the methods involved? |  |  |  |  |

| [12] Han A, Brown D. Experiences of Caregivers in a Dementia Simulation Program. Journal of Social Service Research. 2019. | | | | | |
| --- | --- | --- | --- | --- | --- |
|  | | | | | |
| **Category of study designs** | **Methodological quality criteria** | **Responses** | | | |
|  |  | Yes | No | Can’t tell | Comments |
| Screening questions  (for all types) | S1. Are there clear research questions? | x |  |  | *Formulated as research objective:*  To explore experience of caregivers of people with dementia who participated in a dementia simulation program. |
|  | S2. Do the collected data allow to address the research questions? | x |  |  | *The data reflect the objective and thus answer the research question.* |
|  | *Further appraisal may not be feasible or appropriate when the answer is ‘No’ or ‘Can’t tell’ to one or both screening questions.* | | | | |
| 1. Qualitative | 1.1. Is the qualitative approach appropriate to answer the research question? | x |  |  | *Since the aim is to explore the experiences of caregivers, the use of a qualitative design is appropriate.* |
|  | 1.2. Are the qualitative data collection methods adequate to address the research question? | x |  |  | A semi-structured interview guide was developed to use as a flexible general guide during the interview. Each interview began with a general question asking about the experience of participating in the DL program: “Could you tell me about your experience of participating in the program?” Further open-ended questions were used to explore the perceived impact of participating in the DL program on their daily lives, understanding of dementia and people with dementia, and strategies in caring for people with dementia.  *Consistency with objective apparent.* |
|  | 1.3. Are the findings adequately derived from the data? | x |  |  | The data analysis process involved using auto coding function to start coding under each of the interview questions, creating and coding at thematic nodes, and  reviewing and reorganizing thematic nodes for final themes. To increase the trustworthiness of the data analysis including credibility and transferability, member checking was used during interviews through the process of reflecting and probing and meetings among researchers were held to check whether emerging themes were  well-grounded and represented in the transcripts. In addition, interpretations and derived meanings against the transcript text were constantly checked to verify that the themes remained reflective of the transcript text, in an effort to increase analytical rigor.  *There is no evidence to suggest otherwise.* |
|  | 1.4. Is the interpretation of results sufficiently substantiated by data? | x |  |  | *The data interpretation was underpinned by quotes that correctly describe the respective topics.* |
|  | 1.5. Is there coherence between qualitative data sources, collection, analysis and interpretation? | x |  |  | *The intended research goals are reflected in the identified themes as well as in the data interpretation.* |
| 2. Quantitative randomized controlled trials | 2.1. Is randomization appropriately performed? |  |  |  |  |
|  | 2.2. Are the groups comparable at baseline? |  |  |  |  |
|  | 2.3. Are there complete outcome data? |  |  |  |  |
|  | 2.4. Are outcome assessors blinded to the intervention provided? |  |  |  |  |
|  | 2.5 Did the participants adhere to the assigned intervention? |  |  |  |  |
| 3. Quantitative non-randomized | 3.1. Are the participants representative of the target population? |  |  |  |  |
|  | 3.2. Are measurements appropriate regarding both the outcome and intervention (or exposure)? |  |  |  |  |
|  | 3.3. Are there complete outcome data? |  |  |  |  |
|  | 3.4. Are the confounders accounted for in the design and analysis? |  |  |  |  |
|  | 3.5. During the study period, is the intervention administered (or exposure occurred) as intended? |  |  |  |  |
| 4. Quantitative descriptive | 4.1. Is the sampling strategy relevant to address the research question? |  |  |  |  |
|  | 4.2. Is the sample representative of the target population? |  |  |  |  |
|  | 4.3. Are the measurements appropriate? |  |  |  |  |
|  | 4.4. Is the risk of nonresponse bias low? |  |  |  |  |
|  | 4.5. Is the statistical analysis appropriate to answer the research question? |  |  |  |  |
| 5. Mixed methods | 5.1. Is there an adequate rationale for using a mixed methods design to address the research question? |  |  |  |  |
|  | 5.2. Are the different components of the study effectively integrated to answer the research question? |  |  |  |  |
|  | 5.3. Are the outputs of the integration of qualitative and quantitative components adequately interpreted? |  |  |  |  |
|  | 5.4. Are divergences and inconsistencies between quantitative and qualitative results adequately addressed? |  |  |  |  |
|  | 5.5. Do the different components of the study adhere to the quality criteria of each tradition of the methods involved? |  |  |  |  |

| [13] Han, A., Kim, T. H., & Hong, H. (2021). A factorial randomized controlled trial to examine separate and combined effects of a simulation-based empathy enhancement program and a lecture-based education program on family caregivers of people with dementia. Aging & mental health, 25(10), 1930-1940. https://doi.org/10.1080/13607863.2020.1768214 | | | | | |
| --- | --- | --- | --- | --- | --- |
|  | | | | | |
| **Category of study designs** | **Methodological quality criteria** | **Responses** | | | |
|  |  | Yes | No | Can’t tell | Comments |
| Screening questions  (for all types) | S1. Are there clear research questions? | x |  |  | *Formulated as research objectives:*   - to evaluate simulation-based education and lecture-based education, together and separately. - to test the effect of a simulation-based empathy enhancement program, the effect of a lecture-based education session, and the combined effect of the two interventions on family caregivers of people with early to middle stages of dementia. |
|  | S2. Do the collected data allow to address the research questions? | x |  |  | *The data reflect the objective (testing the effectiveness of the simulation-based program) and thus answer the research question.* |
|  | *Further appraisal may not be feasible or appropriate when the answer is ‘No’ or ‘Can’t tell’ to one or both screening questions.* | | | | |
| 1. Qualitative | 1.1. Is the qualitative approach appropriate to answer the research question? |  |  |  |  |
|  | 1.2. Are the qualitative data collection methods adequate to address the research question? |  |  |  |  |
|  | 1.3. Are the findings adequately derived from the data? |  |  |  |  |
|  | 1.4. Is the interpretation of results sufficiently substantiated by data? |  |  |  |  |
|  | 1.5. Is there coherence between qualitative data sources, collection, analysis and interpretation? |  |  |  |  |
| 2. Quantitative randomized controlled trials | 2.1. Is randomization appropriately performed? |  |  | x | In the current study, the unit of randomization was at the individual level as recruitment  was from one hospital in one geographical area. The allocation ratio was 1:1:1:1, so the equal number of participants were assigned to one of the four groups. Random  numbers were generated using RAND and RANK functions in Excel.  *The researchers describe how the randomisation plan was created (including the use of randomisation numbers by PC programs). However, it remains unclear whether allocation was concealed.* |
|  | 2.2. Are the groups comparable at baseline? | x |  |  | Table 1 shows characteristics of participants by allocated groups and results on homogeneity test of characteristics among groups. There was no statistically significant difference in characteristics among these four groups. However, participants in the treatment as usual group were younger and higher proportion of participants were more highly educated and daughters, did not live together with relatives with dementia, and spent less time in caregiving compared to participants in the other groups.  *No conclusions about an imbalance between the four groups can be drawn from Table 1.* |
|  | 2.3. Are there complete outcome data? | x |  |  | *From the available data, there is no indication of missing data.* |
|  | 2.4. Are outcome assessors blinded to the intervention provided? |  |  | x | Limitations of the present study should be noted. Due to the nature of the interventions, blinding participants and providers to their allocated groups was not done.  *There is only one indication that participants and providers could not be blinded. It remains unclear whether assessors were blinded.* |
|  | 2.5 Did the participants adhere to the assigned intervention? |  |  | x | *No statement.* |
| 3. Quantitative non-randomized | 3.1. Are the participants representative of the target population? |  |  |  |  |
|  | 3.2. Are measurements appropriate regarding both the outcome and intervention (or exposure)? |  |  |  |  |
|  | 3.3. Are there complete outcome data? |  |  |  |  |
|  | 3.4. Are the confounders accounted for in the design and analysis? |  |  |  |  |
|  | 3.5. During the study period, is the intervention administered (or exposure occurred) as intended? |  |  |  |  |
| 4. Quantitative descriptive | 4.1. Is the sampling strategy relevant to address the research question? |  |  |  |  |
|  | 4.2. Is the sample representative of the target population? |  |  |  |  |
|  | 4.3. Are the measurements appropriate? |  |  |  |  |
|  | 4.4. Is the risk of nonresponse bias low? |  |  |  |  |
|  | 4.5. Is the statistical analysis appropriate to answer the research question? |  |  |  |  |
| 5. Mixed methods | 5.1. Is there an adequate rationale for using a mixed methods design to address the research question? |  |  |  |  |
|  | 5.2. Are the different components of the study effectively integrated to answer the research question? |  |  |  |  |
|  | 5.3. Are the outputs of the integration of qualitative and quantitative components adequately interpreted? |  |  |  |  |
|  | 5.4. Are divergences and inconsistencies between quantitative and qualitative results adequately addressed? |  |  |  |  |
|  | 5.5. Do the different components of the study adhere to the quality criteria of each tradition of the methods involved? |  |  |  |  |

| [14] Han, A., Kim, T. H., & Hong, H. (2020). Experiences of caregivers of people with dementia in a Korean dementia simulation program. Dementia (14713012), 19(7), 2415-2429. https://doi.org/10.1177/1471301218823453 | | | | | |
| --- | --- | --- | --- | --- | --- |
|  | | | | | |
| **Category of study designs** | **Methodological quality criteria** | **Responses** | | | |
|  |  | Yes | No | Can’t tell | Comments |
| Screening questions  (for all types) | S1. Are there clear research questions? | x |  |  | *Formulated as research objective:*  To explore experiences of caregivers of people with dementia who participated in a Korean dementia simulation program. |
|  | S2. Do the collected data allow to address the research questions? | x |  |  | *The data reflect the objective and thus answer the research question.* |
|  | *Further appraisal may not be feasible or appropriate when the answer is ‘No’ or ‘Can’t tell’ to one or both screening questions.* | | | | |
| 1. Qualitative | 1.1. Is the qualitative approach appropriate to answer the research question? | x |  |  | *The phenomenological approach elaborates the way we subjectively and intersubjective experience the world, ourselves and other people from a first-person perspective.* |
|  | 1.2. Are the qualitative data collection methods adequate to address the research question? | x |  |  | A semi-structured interview guide was developed by the first author with extensive experiences in developing the interview guide and qualitative research based on recommendations made by Smith, Flowers, and Larkin (2009) to use as a flexible general guide during the interview. Each interview began with a general question asking about the experience of participating in the simulation program and further open-ended questions were used to explore the perceived impact of participating in the program on their perspectives and caring for people with dementia.  *Consistency with objective apparent.* |
|  | 1.3. Are the findings adequately derived from the data? | x |  |  | Braun and Clarke’s six analytic steps for doing thematic analysis was used (Braun &  Clarke, 2006; Maguire & Delahunt, 2017). (…) To increase the trustworthiness of the data analysis process, specific strategies recommended by Nowell, Norris, White, and Moules (2017) were used. Member checking was used actively during interviews by a process of reflecting and probing and member checking at the end of the interviews was done again to verify correct understanding of what the participant had said and meant. The interviewer (the first author) asked the participant further explanations on what the participant had said and summarized what the participant had said through the interview to check if the interviewer correctly understood what the participant said and meant.  *There is no evidence to suggest otherwise.* |
|  | 1.4. Is the interpretation of results sufficiently substantiated by data? | x |  |  | *The data interpretation was underpinned by quotes that correctly describe the respective topics.* |
|  | 1.5. Is there coherence between qualitative data sources, collection, analysis and interpretation? | x |  |  | *The intended research goals are reflected in the identified themes as well as in the data interpretation.* |
| 2. Quantitative randomized controlled trials | 2.1. Is randomization appropriately performed? |  |  |  |  |
|  | 2.2. Are the groups comparable at baseline? |  |  |  |  |
|  | 2.3. Are there complete outcome data? |  |  |  |  |
|  | 2.4. Are outcome assessors blinded to the intervention provided? |  |  |  |  |
|  | 2.5 Did the participants adhere to the assigned intervention? |  |  |  |  |
| 3. Quantitative non-randomized | 3.1. Are the participants representative of the target population? |  |  |  |  |
|  | 3.2. Are measurements appropriate regarding both the outcome and intervention (or exposure)? |  |  |  |  |
|  | 3.3. Are there complete outcome data? |  |  |  |  |
|  | 3.4. Are the confounders accounted for in the design and analysis? |  |  |  |  |
|  | 3.5. During the study period, is the intervention administered (or exposure occurred) as intended? |  |  |  |  |
| 4. Quantitative descriptive | 4.1. Is the sampling strategy relevant to address the research question? |  |  |  |  |
|  | 4.2. Is the sample representative of the target population? |  |  |  |  |
|  | 4.3. Are the measurements appropriate? |  |  |  |  |
|  | 4.4. Is the risk of nonresponse bias low? |  |  |  |  |
|  | 4.5. Is the statistical analysis appropriate to answer the research question? |  |  |  |  |
| 5. Mixed methods | 5.1. Is there an adequate rationale for using a mixed methods design to address the research question? |  |  |  |  |
|  | 5.2. Are the different components of the study effectively integrated to answer the research question? |  |  |  |  |
|  | 5.3. Are the outputs of the integration of qualitative and quantitative components adequately interpreted? |  |  |  |  |
|  | 5.4. Are divergences and inconsistencies between quantitative and qualitative results adequately addressed? |  |  |  |  |
|  | 5.5. Do the different components of the study adhere to the quality criteria of each tradition of the methods involved? |  |  |  |  |
| [15] Harrington CC, Neil JA, Hardin SR, Roberson DW. Is Perception Reality? Using Person-in-Context Simulation to Promote Empathic Understanding of Dementia Among Nurse Practitioner Students. Nurs Educ Perspect. 2021;42(6):377-9. | | | | | |
|  | | | | | |
| **Category of study designs** | **Methodological quality criteria** | **Responses** | | | |
|  |  | Yes | No | Can’t tell | Comments |
| Screening questions  (for all types) | S1. Are there clear research questions? | x |  |  | *Clearly formulated:*  Two research questions were asked:  1) Is the VDT an effective learning strategy for primary care NP students?  2) How does a virtual dementia simulation experience affect FNP and AGPCNP students’ empathic understanding of the person living with dementia? |
|  | S2. Do the collected data allow to address the research questions? | x |  |  | *The data reflect the objective and thus answer the research questions.* |
|  | *Further appraisal may not be feasible or appropriate when the answer is ‘No’ or ‘Can’t tell’ to one or both screening questions.* | | | | |
| 1. Qualitative | 1.1. Is the qualitative approach appropriate to answer the research question? | x |  |  | *Since the aim is to explore the experiences of students, the use of a qualitative design is appropriate.* |
|  | 1.2. Are the qualitative data collection methods adequate to address the research question? | x |  |  | Four 45-minute focus groups consisting of four to six NP student participants were conducted in the afternoon after the students’ simulation experience. Epistemological integrity from the research design through implementation and analysis provided rigor for the qualitative component of this study. The focus groups enhanced the diversity of  experiences and the triangulation of data.  *Consistency with objective apparent.* |
|  | 1.3. Are the findings adequately derived from the data? |  |  | x | Students checked their transcripts to clarify statements and make corrections. Research team members also reviewed the transcripts to ensure epistemological integrity. Examination of learning theories and research studies in educational arenas provided additional credibility and dependability.  *There is lack of information in the report to answer this item.* |
|  | 1.4. Is the interpretation of results sufficiently substantiated by data? | x |  |  | *The data interpretation was underpinned by quotes that correctly describe the respective topics.* |
|  | 1.5. Is there coherence between qualitative data sources, collection, analysis and interpretation? | x |  |  | *The intended research goals are reflected in the identified themes as well as in the data interpretation.* |
| 2. Quantitative randomized controlled trials | 2.1. Is randomization appropriately performed? |  |  |  |  |
|  | 2.2. Are the groups comparable at baseline? |  |  |  |  |
|  | 2.3. Are there complete outcome data? |  |  |  |  |
|  | 2.4. Are outcome assessors blinded to the intervention provided? |  |  |  |  |
|  | 2.5 Did the participants adhere to the assigned intervention? |  |  |  |  |
| 3. Quantitative non-randomized | 3.1. Are the participants representative of the target population? |  |  | x | After Medical Center Institutional Review Board approval, the VDT simulation experience was provided for AGPCNP and FNP students (N = 44) in the context of clinical coursework in the study setting (a large university and college of nursing in the Southeast). The study sample (n = 20) included students who signed the informed consent, completed the VDT’s inclusive pre- and postintervention questionnaires, and participated in one of four focus groups in the afternoon after their VDT experience.  *It is not clear from the data to what extent the intended group was reached.* |
|  | 3.2. Are measurements appropriate regarding both the outcome and intervention (or exposure)? | x |  |  | *The variable to be measured was clearly defined and suitable for answering the research question; good reliability and validity of the instrument used.*  Second Wind Dreams’ (2019) Likert-style questionnaires were created using language to facilitate laypersons’ reflection on their experience with the VDT. These questionnaires were a required license component of the VDT; therefore, they were administered as written. |
|  | 3.3. Are there complete outcome data? | x |  |  | *Participants included in the analysis: n = 20 (students who signed the informed consent, completed the VDT’s inclusive pre- and post-intervention questionnaires, and participated in one of four focus groups after their VDT experience).* |
|  | 3.4. Are the confounders accounted for in the design and analysis? |  | x |  | *No methods for controlling confounding factors were described.* |
|  | 3.5. During the study period, is the intervention administered (or exposure occurred) as intended? | x |  |  | *There are no changes in exposure status from the intended intervention.* |
| 4. Quantitative descriptive | 4.1. Is the sampling strategy relevant to address the research question? |  |  |  |  |
|  | 4.2. Is the sample representative of the target population? |  |  |  |  |
|  | 4.3. Are the measurements appropriate? |  |  |  |  |
|  | 4.4. Is the risk of nonresponse bias low? |  |  |  |  |
|  | 4.5. Is the statistical analysis appropriate to answer the research question? |  |  |  |  |
| 5. Mixed methods | 5.1. Is there an adequate rationale for using a mixed methods design to address the research question? |  | x |  | *Due to the complexity of the research objective, which consists of many components, the application of a mixed methods design makes sense.*  *However, the rationale is not explained or described in the report.* |
|  | 5.2. Are the different components of the study effectively integrated to answer the research question? |  | x |  | *No, results were only presented side by side and not integrated.* |
|  | 5.3. Are the outputs of the integration of qualitative and quantitative components adequately interpreted? |  | x |  | *As no data integration took place, no meta-inferences were derived.* |
|  | 5.4. Are divergences and inconsistencies between quantitative and qualitative results adequately addressed? |  |  | x | *No statement possible, as data was not integrated or compared.* |
|  | 5.5. Do the different components of the study adhere to the quality criteria of each tradition of the methods involved? |  | x |  | *Presence of medium to low quality, as partial studies are of medium quality.* |

| [16] Hattink BJJ, Meiland FJM, Campman CAM, Rietsema J, Sitskoorn M, Dröes R-M. Zelf dementie ervaren: Ontwikkeling en evaluatie van de Into D’mentia simulator [Experiencing dementia: evaluation of Into D'mentia]. Tijdschr Gerontol Geriatr. 2015;46(5):262-81. | | | | | |
| --- | --- | --- | --- | --- | --- |
|  | | | | | |
| **Category of study designs** | **Methodological quality criteria** | **Responses** | | | |
|  |  | Yes | No | Can’t tell | Comments |
| Screening questions  (for all types) | S1. Are there clear research questions? | x |  |  | *Clearly formulated as research objective: To develop and evaluate Into D'mentia* |
|  | S2. Do the collected data allow to address the research questions? | x |  |  | *By conducting a qualitative and quantitative study: Explorative study use of semi-structured interviews and questionnaires. Qualitative components are hardly described in the manuscript, if at all.* |
|  | *Further appraisal may not be feasible or appropriate when the answer is ‘No’ or ‘Can’t tell’ to one or both screening questions.* | | | | |
| 1. Qualitative | 1.1. Is the qualitative approach appropriate to answer the research question? |  |  |  |  |
|  | 1.2. Are the qualitative data collection methods adequate to address the research question? |  |  |  |  |
|  | 1.3. Are the findings adequately derived from the data? |  |  |  |  |
|  | 1.4. Is the interpretation of results sufficiently substantiated by data? |  |  |  |  |
|  | 1.5. Is there coherence between qualitative data sources, collection, analysis and interpretation? |  |  |  |  |
| 2. Quantitative randomized controlled trials | 2.1. Is randomization appropriately performed? |  |  |  |  |
|  | 2.2. Are the groups comparable at baseline? |  |  |  |  |
|  | 2.3. Are there complete outcome data? |  |  |  |  |
|  | 2.4. Are outcome assessors blinded to the intervention provided? |  |  |  |  |
|  | 2.5 Did the participants adhere to the assigned intervention? |  |  |  |  |
| 3. Quantitative non-randomized | 3.1. Are the participants representative of the target population? |  |  | x | *For informal caregivers and professionals; In the end, 33 people participated in the study. All which stem from Tilburg area (80 was foreseen). There were several participants who did not fill out the questionnaires at follow-up.* |
|  | 3.2. Are measurements appropriate regarding both the outcome and intervention (or exposure)? | x |  |  | *Short description: this article describes the development of Into D’mentia and a first (explorative) evaluation on the user-friendliness and effects. Regarding the effectiveness (secondary outcomes):*  *Informal caregiver: empathy (IRI), sense of competence (SSCQ), coping strategies (BriefCope), and quality of relationship (questions LSoG)*  *Professional: Empathy (IRI), experience-oriented skills (BOD-list), attitudes (ADQ), work satisfaction (MAS-GZ).* |
|  | 3.3. Are there complete outcome data? |  | x |  | *20/29 completed the last questionnaire (table 4).* |
|  | 3.4. Are the confounders accounted for in the design and analysis? |  | x |  | *Statistical analysis comprised descriptive statistics and other statistics to compare mean differences, like the t-test. No adjustment has taken place.* |
|  | 3.5. During the study period, is the intervention administered (or exposure occurred) as intended? | x |  |  | There are no changes in exposure status from the intended intervention. |
| 4. Quantitative descriptive | 4.1. Is the sampling strategy relevant to address the research question? |  |  |  |  |
|  | 4.2. Is the sample representative of the target population? |  |  |  |  |
|  | 4.3. Are the measurements appropriate? |  |  |  |  |
|  | 4.4. Is the risk of nonresponse bias low? |  |  |  |  |
|  | 4.5. Is the statistical analysis appropriate to answer the research question? |  |  |  |  |
| 5. Mixed methods | 5.1. Is there an adequate rationale for using a mixed methods design to address the research question? |  |  |  |  |
|  | 5.2. Are the different components of the study effectively integrated to answer the research question? |  |  |  |  |
|  | 5.3. Are the outputs of the integration of qualitative and quantitative components adequately interpreted? |  |  |  |  |
|  | 5.4. Are divergences and inconsistencies between quantitative and qualitative results adequately addressed? |  |  |  |  |
|  | 5.5. Do the different components of the study adhere to the quality criteria of each tradition of the methods involved? |  |  |  |  |

| [17] Haugland, V. L., & Reime, M. H. (2018). Scenario-based simulation training as a method to increase nursing students' competence in demanding situations in dementia care. A mixed method study. Nurse Education in Practice, 33, 164-171. <https://doi.org/10.1016/j.nepr.2018.08.008> | | | | | |
| --- | --- | --- | --- | --- | --- |
|  | | | | | |
| **Category of study designs** | **Methodological quality criteria** | **Responses** | | | |
|  |  | Yes | No | Can’t tell | Comments |
| Screening questions  (for all types) | S1. Are there clear research questions? | x |  |  | *Formulated as research objective:*  To explore if scenario-based simulation training can increase nursing students’ communication skills in demanding situations in dementia care, improve their ethical reflection and become more aware of situations considered as use of coercion. |
|  | S2. Do the collected data allow to address the research questions? | x |  |  | *Party, but data collection included focus groups, quantitative questionnaires and observations to cover all research objectives.* |
|  | *Further appraisal may not be feasible or appropriate when the answer is ‘No’ or ‘Can’t tell’ to one or both screening questions.* | | | | |
| 1. Qualitative | 1.1. Is the qualitative approach appropriate to answer the research question? |  |  | x | *No approach described.* |
|  | 1.2. Are the qualitative data collection methods adequate to address the research question? | x |  |  | Two focus groups were carried out inspired from descriptions from Krueger and Casey (2015), using opening, introductory, transitional, key and ending questions.  We used focus groups, questionnaires and observations to explore the participants'  experiences. This methodology was expected to complement the different aspects of the purpose of the study (Creswell, 2014; Tashakkori and Teddlie, 2010). A mixed method approach gave us the opportunity to triangulate findings from the overall data during the interpretation process.  The focus groups were based on experiences and observations from the scenarios while the questionnaires focused on simulation as didactic method as well. |
|  | 1.3. Are the findings adequately derived from the data? |  |  |  | The analyses were performed according to qualitative content analysis described by Graneheim and Lundman (2004). To obtain a sense of the whole, the two authors read the transcript several times separately. Each researcher identified the natural meaning units of the text, condensed it to a description close to the text; the manifest content.  Then the researchers discussed the manifest content, how to interpret and present it; the latent content. Through discussions and reflections of the latent content, sub-themes and themes were decided (Table 2). An example of the analytical process is presented in Table 3. |
|  | 1.4. Is the interpretation of results sufficiently substantiated by data? | x |  |  | *Interpretations are underpinned by quotes and further examples.* |
|  | 1.5. Is there coherence between qualitative data sources, collection, analysis and interpretation? | x |  |  | *The intended research goals are reflected in the identified themes as well as in the data interpretation.* |
| 2. Quantitative randomized controlled trials | 2.1. Is randomization appropriately performed? |  |  |  |  |
|  | 2.2. Are the groups comparable at baseline? |  |  |  |  |
|  | 2.3. Are there complete outcome data? |  |  |  |  |
|  | 2.4. Are outcome assessors blinded to the intervention provided? |  |  |  |  |
|  | 2.5 Did the participants adhere to the assigned intervention? |  |  |  |  |
| 3. Quantitative non-randomized | 3.1. Are the participants representative of the target population? |  |  |  |  |
|  | 3.2. Are measurements appropriate regarding both the outcome and intervention (or exposure)? |  |  |  |  |
|  | 3.3. Are there complete outcome data? |  |  |  |  |
|  | 3.4. Are the confounders accounted for in the design and analysis? |  |  |  |  |
|  | 3.5. During the study period, is the intervention administered (or exposure occurred) as intended? |  |  |  |  |
| 4. Quantitative descriptive | 4.1. Is the sampling strategy relevant to address the research question? |  |  | x | Twelve first-year bachelor nursing students were invited to participate in the simulation training course and the study during their practice placement in nursing homes.  *No statement on sampling strategy.* |
|  | 4.2. Is the sample representative of the target population? |  |  | x | *No statement can be made on this; the demographic data is only presented in brief. Furthermore, the sample is small, so that a representation cannot be concluded.*  *As the target group of the intervention is not described, it remains unclear whether and to what extent the included participants represent the target group.* |
|  | 4.3. Are the measurements appropriate? |  | x |  | *The variables to be measured are partly clearly defined (consisting of participant demographics and open-ended questions on simulation as a didactic method, knowledge of legislation, learning experiences and recommendations for further simulation training) and accurately measured (see overview of questions and categories in Table 4).*  *To which extent validated instruments were used cannot be specified.* |
|  | 4.4. Is the risk of nonresponse bias low? |  |  | x | *N = 12 low, but data are available for all 12 included participants.* |
|  | 4.5. Is the statistical analysis appropriate to answer the research question? | x |  |  | Questionnaires were analysed according to Malterud's (2011) method of content analysis. The open-ended questions were transcribed verbatim. Results were obtained by counting distribution of textual elements in qualitative data, and respondents' answers were placed in relevant categories. The categories represent empirical findings. Finally, the frequency for each category was summarized (Table 4). Polit  and Beck (2012) describe this as a method of transforming qualitative data into numerical values. Observations are presented as counts on the checklist for each team (Table 5). |
| 5. Mixed methods | 5.1. Is there an adequate rationale for using a mixed methods design to address the research question? | x |  |  | Complexity of the research objective, consisting of many components:  concurrent mixed-method design was chosen (Fig. 1). We used focus groups, questionnaires and observations to explore the participants' experiences. This methodology was expected to complement the different aspects of the purpose of the study (Creswell, 2014; Tashakkori and Teddlie, 2010). A mixed method approach gave us the opportunity to triangulate findings from the overall data during the  interpretation process. |
|  | 5.2. Are the different components of the study effectively integrated to answer the research question? |  | x |  | *No, results were only presented side by side and not integrated.* |
|  | 5.3. Are the outputs of the integration of qualitative and quantitative components adequately interpreted? |  | x |  | *As no data integration took place, no meta-inferences were derived.* |
|  | 5.4. Are divergences and inconsistencies between quantitative and qualitative results adequately addressed? |  |  | x | *No statement possible, as data was not integrated or compared.* |
|  | 5.5. Do the different components of the study adhere to the quality criteria of each tradition of the methods involved? |  | x |  | *Presence of low quality, as partial studies are of medium to low quality.* |

| [18] Heward M, Board M, Spriggs A, Emerson L, Murphy J. Impact of 'DEALTS2' education intervention on trainer dementia knowledge and confidence to utilise innovative training approaches: A national pre-test - post-test survey. Nurse Educ Today. 2021;97:104694. | | | | | |
| --- | --- | --- | --- | --- | --- |
|  | | | | | |
| **Category of study designs** | **Methodological quality criteria** | **Responses** | | | |
|  |  | Yes | No | Can’t tell | Comments |
| Screening questions  (for all types) | S1. Are there clear research questions? | x |  |  | *Clearly formulated research questions (in Heward et al, 2019):*  The following research questions will be addressed:   - How has DEALTS 2 been implemented (adopted and/or adapted) by those attending Train the Trainer workshops across England? - What are trainers’ perceptions and experiences of DEALTS 2? - What are barriers and enablers to the implementation of DEALTS 2 across England? - What are health professionals’ experiences of DEALTS 2? - What is the impact of DEALTS 2 on trainers and health professional’s knowledge of dementia and care approach? - Are there wider impacts from DEALTS 2 in individual NHS Trusts, including integration of theory into practice and service improvement? |
|  | S2. Do the collected data allow to address the research questions? | x |  |  | In this paper we report the quantitative results from the pre-test – post-test survey completed by trainers that attended the DEALTS2 TTT workshops in Phase 1 of the study.  *Taken all together, the data presented reflect the objective and thus answer the research questions.* |
|  | *Further appraisal may not be feasible or appropriate when the answer is ‘No’ or ‘Can’t tell’ to one or both screening questions.* | | | | |
| 1. Qualitative | 1.1. Is the qualitative approach appropriate to answer the research question? |  |  |  |  |
|  | 1.2. Are the qualitative data collection methods adequate to address the research question? |  |  |  |  |
|  | 1.3. Are the findings adequately derived from the data? |  |  |  |  |
|  | 1.4. Is the interpretation of results sufficiently substantiated by data? |  |  |  |  |
|  | 1.5. Is there coherence between qualitative data sources, collection, analysis and interpretation? |  |  |  |  |
| 2. Quantitative randomized controlled trials | 2.1. Is randomization appropriately performed? |  |  |  |  |
|  | 2.2. Are the groups comparable at baseline? |  |  |  |  |
|  | 2.3. Are there complete outcome data? |  |  |  |  |
|  | 2.4. Are outcome assessors blinded to the intervention provided? |  |  |  |  |
|  | 2.5 Did the participants adhere to the assigned intervention? |  |  |  |  |
| 3. Quantitative non-randomized | 3.1. Are the participants representative of the target population? |  |  | x | *In this paper and in the protocol (Heward et al., 2019) there is little meaningful data on participants and the target group of the training respectively of the evaluation. It is not clear from the data to what extent the intended group was reached.* |
|  | 3.2. Are measurements appropriate regarding both the outcome and intervention (or exposure)? |  |  | x | *The variable to be measured was clearly defined and suitable for answering the research question; probably self-developed instruments (missing data).*  The pre-test – post-test survey using Likert scales and closed ended questions measured dementia knowledge scores, satisfaction, spread of implementation, and confidence. |
|  | 3.3. Are there complete outcome data? | x |  |  | *Participants included in the intervention: n = 199*  *Participants included in analysis: n = 183* |
|  | 3.4. Are the confounders accounted for in the design and analysis? |  | x |  | *No methods for controlling confounding factors were described.* |
|  | 3.5. During the study period, is the intervention administered (or exposure occurred) as intended? | x |  |  | *There are no changes in exposure status from the intended intervention.* |
| 4. Quantitative descriptive | 4.1. Is the sampling strategy relevant to address the research question? |  |  |  |  |
|  | 4.2. Is the sample representative of the target population? |  |  |  |  |
|  | 4.3. Are the measurements appropriate? |  |  |  |  |
|  | 4.4. Is the risk of nonresponse bias low? |  |  |  |  |
|  | 4.5. Is the statistical analysis appropriate to answer the research question? |  |  |  |  |
| 5. Mixed methods | 5.1. Is there an adequate rationale for using a mixed methods design to address the research question? |  |  |  |  |
|  | 5.2. Are the different components of the study effectively integrated to answer the research question? |  |  |  |  |
|  | 5.3. Are the outputs of the integration of qualitative and quantitative components adequately interpreted? |  |  |  |  |
|  | 5.4. Are divergences and inconsistencies between quantitative and qualitative results adequately addressed? |  |  |  |  |
|  | 5.5. Do the different components of the study adhere to the quality criteria of each tradition of the methods involved? |  |  |  |  |

| [19] Jeong H, Kim TH, Han AR. Effects of Korean dementia simulation program for caregivers of the elderly with dementia pilot study. European Neuropsychopharmacology. 2019;29:S130-S1. | | | | | |
| --- | --- | --- | --- | --- | --- |
|  | | | | | |
| **Category of study designs** | **Methodological quality criteria** | **Responses** | | | |
|  |  | Yes | No | Can’t tell | Comments |
| Screening questions  (for all types) | S1. Are there clear research questions? | x |  |  | *Formulated as research objective:*  To evaluate the effectiveness having experience using simulation program for dementia patient caregivers on the empathy in Korea. |
|  | S2. Do the collected data allow to address the research questions? |  | x |  | *The measurement of the effectiveness of an intervention can only be achieved through an randomised controlled trial. The present pre-post test design can only provide information about the correlation and thus the relationship to the intervention.* |
|  | *Further appraisal may not be feasible or appropriate when the answer is ‘No’ or ‘Can’t tell’ to one or both screening questions.* | | | | |
| 1. Qualitative | 1.1. Is the qualitative approach appropriate to answer the research question? |  |  |  |  |
|  | 1.2. Are the qualitative data collection methods adequate to address the research question? |  |  |  |  |
|  | 1.3. Are the findings adequately derived from the data? |  |  |  |  |
|  | 1.4. Is the interpretation of results sufficiently substantiated by data? |  |  |  |  |
|  | 1.5. Is there coherence between qualitative data sources, collection, analysis and interpretation? |  |  |  |  |
| 2. Quantitative randomized controlled trials | 2.1. Is randomization appropriately performed? |  |  |  |  |
|  | 2.2. Are the groups comparable at baseline? |  |  |  |  |
|  | 2.3. Are there complete outcome data? |  |  |  |  |
|  | 2.4. Are outcome assessors blinded to the intervention provided? |  |  |  |  |
|  | 2.5 Did the participants adhere to the assigned intervention? |  |  |  |  |
| 3. Quantitative non-randomized | 3.1. Are the participants representative of the target population? |  |  | x | *The objective included "dementia patient caregivers in Korea"; the 28 caregivers included (n = 16 family caregivers and n = 12 formal caregivers) reflect this group.*  *However, this does not provide a basis for extending the results to the entire group of caregivers in Korea.* |
|  | 3.2. Are measurements appropriate regarding both the outcome and intervention (or exposure)? | x |  |  | We tested for the effect of the program on the empathy through Dementia Attitude Scale(DAS), Korean version of Interpersonal Reactivity Index (K-IRI), short Zarit Burden  Inventory (S-ZBI), Center for Epidemiologic Studies Depression Scale –10 (CES-D-10), Brief coping orientation to problems experienced (Brief COPE), and General Self-efficacy  Scale (GSE).  *The variables to be measured were clearly defined and therefore suitable for answering the research objective. The instruments used were validated instruments*. |
|  | 3.3. Are there complete outcome data? |  |  | x | *Participants included in the intervention: No information provided*  *Participants included in analysis: n = 28 (n = 16 family caregivers and n = 12 formal caregivers)* |
|  | 3.4. Are the confounders accounted for in the design and analysis? |  | x |  | *No methods for controlling confounding factors were described.* |
|  | 3.5. During the study period, is the intervention administered (or exposure occurred) as intended? |  |  | x | *No information provided* |
| 4. Quantitative descriptive | 4.1. Is the sampling strategy relevant to address the research question? |  |  |  |  |
|  | 4.2. Is the sample representative of the target population? |  |  |  |  |
|  | 4.3. Are the measurements appropriate? |  |  |  |  |
|  | 4.4. Is the risk of nonresponse bias low? |  |  |  |  |
|  | 4.5. Is the statistical analysis appropriate to answer the research question? |  |  |  |  |
| 5. Mixed methods | 5.1. Is there an adequate rationale for using a mixed methods design to address the research question? |  |  |  |  |
|  | 5.2. Are the different components of the study effectively integrated to answer the research question? |  |  |  |  |
|  | 5.3. Are the outputs of the integration of qualitative and quantitative components adequately interpreted? |  |  |  |  |
|  | 5.4. Are divergences and inconsistencies between quantitative and qualitative results adequately addressed? |  |  |  |  |
|  | 5.5. Do the different components of the study adhere to the quality criteria of each tradition of the methods involved? |  |  |  |  |

| [20] Jütten LH, Mark RE, Sitskoorn MM. Can the Mixed Virtual Reality Simulator Into D'mentia Enhance Empathy and Understanding and Decrease Burden in Informal Dementia Caregivers? Dementia and Geriatric Cognitive Disorders Extra. 2018;8(3):453-66. | | | | | |
| --- | --- | --- | --- | --- | --- |
|  | | | | | |
| **Category of study designs** | **Methodological quality criteria** | **Responses** | | | |
|  |  | Yes | No | Can’t tell | Comments |
| Screening questions  (for all types) | S1. Are there clear research questions? | x |  |  | *Formulated as research objective:*  To examine whether the Into D'mentia training increased understanding of dementia and cognitive empathy in informal caregivers (secondary aims) whether the Into D'mentia training decreased caregiver burden, depression, and anxiety, and/or enhanced sense of competence and relationship quality with the care receiver. |
|  | S2. Do the collected data allow to address the research questions? | x |  |  | *The data reflect the objective and thus answer the research question:*  The current study is a quasi-experimental longitudinal investigation into the effectiveness of the Into D’mentia simulator. The outcome variables were assessed at four time points: 1 week before the Into D’mentia training (T1), 1 week after the training (T2), 2.5 months after the training (T3), and 15 months after the training (T4) to examine both short and long-term effects. |
|  | *Further appraisal may not be feasible or appropriate when the answer is ‘No’ or ‘Can’t tell’ to one or both screening questions.* | | | | |
| 1. Qualitative | 1.1. Is the qualitative approach appropriate to answer the research question? |  |  |  |  |
|  | 1.2. Are the qualitative data collection methods adequate to address the research question? |  |  |  |  |
|  | 1.3. Are the findings adequately derived from the data? |  |  |  |  |
|  | 1.4. Is the interpretation of results sufficiently substantiated by data? |  |  |  |  |
|  | 1.5. Is there coherence between qualitative data sources, collection, analysis and interpretation? |  |  |  |  |
| 2. Quantitative randomized controlled trials | 2.1. Is randomization appropriately performed? |  |  |  |  |
|  | 2.2. Are the groups comparable at baseline? |  |  |  |  |
|  | 2.3. Are there complete outcome data? |  |  |  |  |
|  | 2.4. Are outcome assessors blinded to the intervention provided? |  |  |  |  |
|  | 2.5 Did the participants adhere to the assigned intervention? |  |  |  |  |
| 3. Quantitative non-randomized | 3.1. Are the participants representative of the target population? |  |  | x | The target population of the intervention was described; the sample (incl. inclusion and exclusion criteria) was equally clearly stated. However, the extent to which the target population was thus represented remains unclear, as also presented by Jütten et al. (2018):  However, the caregiver population is very heterogeneous; caregivers differ from each other on a number of caregiver-related variables (e.g., age, sex, educational level), care receiver variables (e.g., time since diagnosis), and background variables (e.g., time spent on caregiving, whether or not they live with the care receiver). In addition, caregivers differ in their baseline empathy levels. |
|  | 3.2. Are measurements appropriate regarding both the outcome and intervention (or exposure)? | x |  |  | *The variables to be measured were clearly defined and thus suitable for answering the research question; good reliability and validity of the questionnaires used. The questions of the semi-structured interview were disclosed in Table 2.* |
|  | 3.3. Are there complete outcome data? | x |  |  | *Participants included in the intervention: n = 201 (n = 145 in the intervention group; n = 56 control group); Participants included in analysis: n = 201 (n = 145 in the intervention group; n = 56 control group).* |
|  | 3.4. Are the confounders accounted for in the design and analysis? |  |  | x | *A suitable method for controlling confounding factors was applied by matching, but the impact of matching cannot completely eliminate confounding:*  The groups were (successfully) group-matched on sex and level of education. However, the control group was slightly (but significantly) older than the intervention group, spent more hours on caregiving, and experienced more anxiety and burden than the intervention group. |
|  | 3.5. During the study period, is the intervention administered (or exposure occurred) as intended? | x |  |  | *Compared to the published protocol (describes intended intervention), no changes could be identified.* |
| 4. Quantitative descriptive | 4.1. Is the sampling strategy relevant to address the research question? |  |  |  |  |
|  | 4.2. Is the sample representative of the target population? |  |  |  |  |
|  | 4.3. Are the measurements appropriate? |  |  |  |  |
|  | 4.4. Is the risk of nonresponse bias low? |  |  |  |  |
|  | 4.5. Is the statistical analysis appropriate to answer the research question? |  |  |  |  |
| 5. Mixed methods | 5.1. Is there an adequate rationale for using a mixed methods design to address the research question? |  |  |  |  |
|  | 5.2. Are the different components of the study effectively integrated to answer the research question? |  |  |  |  |
|  | 5.3. Are the outputs of the integration of qualitative and quantitative components adequately interpreted? |  |  |  |  |
|  | 5.4. Are divergences and inconsistencies between quantitative and qualitative results adequately addressed? |  |  |  |  |
|  | 5.5. Do the different components of the study adhere to the quality criteria of each tradition of the methods involved? |  |  |  |  |

| [21] Kimzey M, Mastel-Smith B. Impact of dementia simulation on nursing students: When empathy breeds awareness. Teaching & Learning in Nursing. 2021. | | | | | |
| --- | --- | --- | --- | --- | --- |
|  | | | | | |
| **Category of study designs** | **Methodological quality criteria** | **Responses** | | | |
|  |  | Yes | No | Can’t tell | Comments |
| Screening questions  (for all types) | S1. Are there clear research questions? | x |  |  | *Clearly formulated as research objective:*  The purpose of this study was to explore the impact of the Virtual Dementia Tour on nursing students’ attitudes toward and empathy for people with dementia, dementia knowledge, self-confidence to care for people with dementia and impact on future nursing care. |
|  | S2. Do the collected data allow to address the research questions? | x |  |  | *The data reflect the objective and thus answer the research question:*  This article describes the qualitative strand of an embedded mixed methods study. The qualitative strand provided a better understanding of the VDT intervention because it gave the students a voice. The qualitative findings supported the quantitative findings (Kimzey et al., 2019). Phenomenology describes how people perceive experiences within a particular context (Portney & Watkins, 2015), in this case students' perceptions of the intervention. |
|  | *Further appraisal may not be feasible or appropriate when the answer is ‘No’ or ‘Can’t tell’ to one or both screening questions.* | | | | |
| 1. Qualitative | 1.1. Is the qualitative approach appropriate to answer the research question? | x |  |  | This approach recognized the subjectivity of the VDT experience, allowed the researcher to be active in the process, and the data was from the students experiencing the VDT (Bradshaw et al., 2017). Descriptive phenomenology allowed the researchers to focus on the description of students’ experiences in the simulation. |
|  | 1.2. Are the qualitative data collection methods adequate to address the research question? | x |  |  | Data were derived from two sources. Students answered via paper and pencil two open-ended questions after the Virtual Dementia Tour: “How did Virtual Dementia Tour impact or change you?” and “What would you do differently because of Virtual Dementia Tour?” After post-test data was collected, students were invited to partici-pate in a one time, audio taped focus group interview. During focus groups, a semi-structured interview schedule guided data collection and explored students’ perceptions of dementia and the impact the Virtual Dementia Tour had on attitudes toward and empathy for people with dementia, dementia knowledge and self-confidence to care for people with dementia. |
|  | 1.3. Are the findings adequately derived from the data? | x |  |  | Colaizzi’s (1978) seven-step method guided qualitative data analysis. |
|  | 1.4. Is the interpretation of results sufficiently substantiated by data? | x |  |  | *Interpretations are underpinned by quotes and further examples.* |
|  | 1.5. Is there coherence between qualitative data sources, collection, analysis and interpretation? | x |  |  | *The intended research goals are reflected in the identified themes as well as in the data interpretation.* |
| 2. Quantitative randomized controlled trials | 2.1. Is randomization appropriately performed? |  |  |  |  |
|  | 2.2. Are the groups comparable at baseline? |  |  |  |  |
|  | 2.3. Are there complete outcome data? |  |  |  |  |
|  | 2.4. Are outcome assessors blinded to the intervention provided? |  |  |  |  |
|  | 2.5 Did the participants adhere to the assigned intervention? |  |  |  |  |
| 3. Quantitative non-randomized | 3.1. Are the participants representative of the target population? |  |  |  |  |
|  | 3.2. Are measurements appropriate regarding both the outcome and intervention (or exposure)? |  |  |  |  |
|  | 3.3. Are there complete outcome data? |  |  |  |  |
|  | 3.4. Are the confounders accounted for in the design and analysis? |  |  |  |  |
|  | 3.5. During the study period, is the intervention administered (or exposure occurred) as intended? |  |  |  |  |
| 4. Quantitative descriptive | 4.1. Is the sampling strategy relevant to address the research question? |  |  |  |  |
|  | 4.2. Is the sample representative of the target population? |  |  |  |  |
|  | 4.3. Are the measurements appropriate? |  |  |  |  |
|  | 4.4. Is the risk of nonresponse bias low? |  |  |  |  |
|  | 4.5. Is the statistical analysis appropriate to answer the research question? |  |  |  |  |
| 5. Mixed methods | 5.1. Is there an adequate rationale for using a mixed methods design to address the research question? |  |  |  |  |
|  | 5.2. Are the different components of the study effectively integrated to answer the research question? |  |  |  |  |
|  | 5.3. Are the outputs of the integration of qualitative and quantitative components adequately interpreted? |  |  |  |  |
|  | 5.4. Are divergences and inconsistencies between quantitative and qualitative results adequately addressed? |  |  |  |  |
|  | 5.5. Do the different components of the study adhere to the quality criteria of each tradition of the methods involved? |  |  |  |  |

| [22] Kimzey, M., Mastel-Smith, B., & Seale, A. (2019). Effects of Dementia-Specific Education for Nursing Students. Nurse Educator, 44(6), 338-341. https://doi.org/10.1097/NNE.0000000000000623 | | | | | |
| --- | --- | --- | --- | --- | --- |
|  | | | | | |
| **Category of study designs** | **Methodological quality criteria** | **Responses** | | | |
|  |  | Yes | No | Can’t tell | Comments |
| Screening questions  (for all types) | S1. Are there clear research questions? | x |  |  | *Formulated as research objective:*  To determine the effects of a virtual dementia experience on nursing students' attitudes and empathy for people with dementia, dementia knowledge, and self-confidence for dementia care. |
|  | S2. Do the collected data allow to address the research questions? |  | x |  | *The measurement of the effectiveness of an intervention can only be achieved through an RCT. The present quasi-experimental design (with pre-/post-test) can only provide information about the correlation and thus the relationship of outcomes to the intervention.* |
|  | *Further appraisal may not be feasible or appropriate when the answer is ‘No’ or ‘Can’t tell’ to one or both screening questions.* | | | | |
| 1. Qualitative | 1.1. Is the qualitative approach appropriate to answer the research question? |  |  |  |  |
|  | 1.2. Are the qualitative data collection methods adequate to address the research question? |  |  |  |  |
|  | 1.3. Are the findings adequately derived from the data? |  |  |  |  |
|  | 1.4. Is the interpretation of results sufficiently substantiated by data? |  |  |  |  |
|  | 1.5. Is there coherence between qualitative data sources, collection, analysis and interpretation? |  |  |  |  |
| 2. Quantitative randomized controlled trials | 2.1. Is randomization appropriately performed? |  |  |  |  |
|  | 2.2. Are the groups comparable at baseline? |  |  |  |  |
|  | 2.3. Are there complete outcome data? |  |  |  |  |
|  | 2.4. Are outcome assessors blinded to the intervention provided? |  |  |  |  |
|  | 2.5 Did the participants adhere to the assigned intervention? |  |  |  |  |
| 3. Quantitative non-randomized | 3.1. Are the participants representative of the target population? |  |  | x | The convenience sample consisted of baccalaureate students concurrently enrolled in a required Behavioral Health course in a nursing program in southwest United States.  *The objective included "nursing students"; the 112 nursing students from the university reflect this group. However, this does not provide a basis for extending the results to the entire group of nursing students.* |
|  | 3.2. Are measurements appropriate regarding both the outcome and intervention (or exposure)? | x |  |  | *The variables to be measured were clearly defined and therefore suitable for answering the research objective. The instruments used were validated instruments: kDAS, IRI, DKAT2, and CODE.* |
|  | 3.3. Are there complete outcome data? | x |  |  | *Participants included in the intervention: n = 112 nursing students (IG: n = 56 students, CG: n = 56 students).*  *Participants included in analysis: n = 108 (There were 81 matched paired pre-test/post-test surveys; 47 students in the intervention group completed the virtual dementia experience, and 34 were in the control group.)* |
|  | 3.4. Are the confounders accounted for in the design and analysis? | x |  |  | *A suitable method to control for confounding factors was applied through matching.* |
|  | 3.5. During the study period, is the intervention administered (or exposure occurred) as intended? | x |  |  | *There are no changes in exposure status from the intended intervention.* |
| 4. Quantitative descriptive | 4.1. Is the sampling strategy relevant to address the research question? |  |  |  |  |
|  | 4.2. Is the sample representative of the target population? |  |  |  |  |
|  | 4.3. Are the measurements appropriate? |  |  |  |  |
|  | 4.4. Is the risk of nonresponse bias low? |  |  |  |  |
|  | 4.5. Is the statistical analysis appropriate to answer the research question? |  |  |  |  |
| 5. Mixed methods | 5.1. Is there an adequate rationale for using a mixed methods design to address the research question? |  |  |  |  |
|  | 5.2. Are the different components of the study effectively integrated to answer the research question? |  |  |  |  |
|  | 5.3. Are the outputs of the integration of qualitative and quantitative components adequately interpreted? |  |  |  |  |
|  | 5.4. Are divergences and inconsistencies between quantitative and qualitative results adequately addressed? |  |  |  |  |
|  | 5.5. Do the different components of the study adhere to the quality criteria of each tradition of the methods involved? |  |  |  |  |

| [23] Kimzey M, Patterson J, Mastel-Smith B. Effects of Simulation on Nursing Students' Dementia Knowledge and Empathy: A Mixed Method Study. Issues in Mental Health Nursing. 2020. | | | | | |
| --- | --- | --- | --- | --- | --- |
|  | | | | | |
| **Category of study designs** | **Methodological quality criteria** | **Responses** | | | |
|  |  | Yes | No | Can’t tell | Comments |
| Screening questions  (for all types) | S1. Are there clear research questions? | x |  |  | *Formulated as research objective:*  To determine the effects of dementia simulation on nursing student's dementia knowledge and empathy for people living with dementia.  (Hypothesis: Nursing students will report improved dementia knowledge and empathy for people with dementia after the dementia simulation.  Qualitative research question: How did the dementia simulation affect nursing students’ knowledge of dementia and empathy for people with dementia?) |
|  | S2. Do the collected data allow to address the research questions? | x |  |  | *Data collection included focus groups and quantitative questionnaires (pre-post; before and after the simulation) to cover the research objective.* |
|  | *Further appraisal may not be feasible or appropriate when the answer is ‘No’ or ‘Can’t tell’ to one or both screening questions.* | | | | |
| 1. Qualitative | 1.1. Is the qualitative approach appropriate to answer the research question? | x |  |  | The qualitative strand of the project consisted of eight focus groups consisting of  six and eight students per group and occurred immediately after the debriefing of the intervention. A semi-structured interview guide was used (…) were prompted to  discuss thoughts, feelings, motivation to help, and perspective-taking. Sample questions included “What feelings did you experience during the simulation?” and “How is your current life different than the life of someone with dementia?” |
|  | 1.2. Are the qualitative data collection methods adequate to address the research question? | x |  |  | *Yes, see above.* |
|  | 1.3. Are the findings adequately derived from the data? | x |  |  | Thematic analysis was utilized for the qualitative data. One co-investigator and an additional researcher with extensive qualitative research experience reviewed the verbatim transcripts. Both members coded the data independently and met regularly for interpretive convergence (Saldana, 2016).  *Probably yes* |
|  | 1.4. Is the interpretation of results sufficiently substantiated by data? | x |  |  | *The data interpretation was underpinned by quotes that correctly describe the respective topics.* |
|  | 1.5. Is there coherence between qualitative data sources, collection, analysis and interpretation? | x |  |  | *The intended research goals are reflected in the identified themes as well as in the data interpretation.* |
| 2. Quantitative randomized controlled trials | 2.1. Is randomization appropriately performed? |  |  |  |  |
|  | 2.2. Are the groups comparable at baseline? |  |  |  |  |
|  | 2.3. Are there complete outcome data? |  |  |  |  |
|  | 2.4. Are outcome assessors blinded to the intervention provided? |  |  |  |  |
|  | 2.5 Did the participants adhere to the assigned intervention? |  |  |  |  |
| 3. Quantitative non-randomized | 3.1. Are the participants representative of the target population? |  |  | x | A convenience sample consisted of undergraduate nursing students enrolled in the required Behavioral Health course. Students completed the intervention in the Health  Professions Care Lab (HPCL) at the school of nursing. Out of 72 students enrolled in the course, 55 students completed surveys and participated in focus groups.  *However, this does not provide a basis for extending the results to the entire group of physical therapy students.* |
|  | 3.2. Are measurements appropriate regarding both the outcome and intervention (or exposure)? | x |  |  | *The variables to be measured were clearly defined and therefore suitable for answering the research objective. The instruments used were validated instruments: DKAT2, CSES* |
|  | 3.3. Are there complete outcome data? |  |  | x | *Participants included in the intervention: n = 72 (students enrolled in the course)*  *Participants included in analysis: n = 55 (completed surveys and participated in focus groups).* |
|  | 3.4. Are the confounders accounted for in the design and analysis? |  | x |  | *No methods to control for confounding factors were applied.* |
|  | 3.5. During the study period, is the intervention administered (or exposure occurred) as intended? | x |  |  | *There are no changes in exposure status from the intended intervention.* |
| 4. Quantitative descriptive | 4.1. Is the sampling strategy relevant to address the research question? |  |  |  |  |
|  | 4.2. Is the sample representative of the target population? |  |  |  |  |
|  | 4.3. Are the measurements appropriate? |  |  |  |  |
|  | 4.4. Is the risk of nonresponse bias low? |  |  |  |  |
|  | 4.5. Is the statistical analysis appropriate to answer the research question? |  |  |  |  |
| 5. Mixed methods | 5.1. Is there an adequate rationale for using a mixed methods design to address the research question? | x |  |  | This study used a mixed methods one group quasi-experimental embedded design. Mixed methods research is beneficial when the research question is one in which the use of one method is inadequate, particularly when seeking a broader and deeper understanding of a complex phenomenon (Doyle et al., 2016). |
|  | 5.2. Are the different components of the study effectively integrated to answer the research question? |  |  | x | *The results were presented merged, but it is unclear how the analysis was done.* |
|  | 5.3. Are the outputs of the integration of qualitative and quantitative components adequately interpreted? |  |  | x | *Probably yes, but there’s no description of data analysis* |
|  | 5.4. Are divergences and inconsistencies between quantitative and qualitative results adequately addressed? |  |  | x | *See above.* |
|  | 5.5. Do the different components of the study adhere to the quality criteria of each tradition of the methods involved? | x |  |  |  |

| [24] Kobiske KR, Deprey SM. A Dementia Simulation as a Teaching Strategy for Nursing and Physical Therapy Students: A Qualitative Study. Clinical Simulation in Nursing. 2022;67:11-7. | | | | | |
| --- | --- | --- | --- | --- | --- |
|  | | | | | |
| **Category of study designs** | **Methodological quality criteria** | **Responses** | | | |
|  |  | Yes | No | Can’t tell | Comments |
| Screening questions  (for all types) | S1. Are there clear research questions? | x |  |  | *Formulated as research objective, however, vague, broad intention:*  To understand baccalaureate nursing (BSN) student and doctor of physical therapy (DPT) student perspectives of the impact of ADRD after experiencing a virtual dementia simulation. |
|  | S2. Do the collected data allow to address the research questions? | x |  |  | *The data reflect the objective and thus answer the research question.* |
|  | *Further appraisal may not be feasible or appropriate when the answer is ‘No’ or ‘Can’t tell’ to one or both screening questions.* | | | | |
| 1. Qualitative | 1.1. Is the qualitative approach appropriate to answer the research question? | x |  |  | A qualitative design was set in an interpretive phenomenology frame to study the experience using insights from participants. Phenomenology is a qualitative experience or perspective-based research design (Munhall, 2012). Participants were asked to respond in narrative format to two semi-structured, open-ended questions to gain an understanding of the student perspective during the simulation experience. |
|  | 1.2. Are the qualitative data collection methods adequate to address the research question? |  |  | x | *Consistency with objective apparent; It is unclear why interviews were not used, which might have led to more comprehensive results.*  Participants were asked to respond in narrative format to two semi-structured, open-ended questions to gain an understanding of the student perspective during the simulation experience. Due to the large sample size, data was collected via survey format which allowed participants the freedom to respond as much as they wanted. |
|  | 1.3. Are the findings adequately derived from the data? | x |  |  | Thematic analysis occurred using a three-step process of (a) familiarizing self with data, (b) searching for meanings and themes, and (c) organizing themes into meaningful wholeness (Sundler et al., 2019). Each researcher completed this three-step process individually. Researchers used the following three-step process of (a) searched for commonalities through reading and coding narratives, (b) journaled reflectively for meanings and patterns to ensure reflexivity, credibility, and transferability (Sundler et al., 2019 ) (c) created a coding system to organize themes. Re- searchers then compared findings. On initial review, re- searchers found identical results for themes for each question. After a thorough discussion on the meaning and understanding of participant narratives, a final theme was added to the thematic analysis results.  *There is no evidence to suggest otherwise.* |
|  | 1.4. Is the interpretation of results sufficiently substantiated by data? | x |  |  | *The data interpretation was underpinned by quotes that correctly describe the respective topics.* |
|  | 1.5. Is there coherence between qualitative data sources, collection, analysis and interpretation? | x |  |  | *The intended research goals are reflected in the identified themes as well as in the data interpretation.* |
| 2. Quantitative randomized controlled trials | 2.1. Is randomization appropriately performed? |  |  |  |  |
|  | 2.2. Are the groups comparable at baseline? |  |  |  |  |
|  | 2.3. Are there complete outcome data? |  |  |  |  |
|  | 2.4. Are outcome assessors blinded to the intervention provided? |  |  |  |  |
|  | 2.5 Did the participants adhere to the assigned intervention? |  |  |  |  |
| 3. Quantitative non-randomized | 3.1. Are the participants representative of the target population? |  |  |  |  |
|  | 3.2. Are measurements appropriate regarding both the outcome and intervention (or exposure)? |  |  |  |  |
|  | 3.3. Are there complete outcome data? |  |  |  |  |
|  | 3.4. Are the confounders accounted for in the design and analysis? |  |  |  |  |
|  | 3.5. During the study period, is the intervention administered (or exposure occurred) as intended? |  |  |  |  |
| 4. Quantitative descriptive | 4.1. Is the sampling strategy relevant to address the research question? |  |  |  |  |
|  | 4.2. Is the sample representative of the target population? |  |  |  |  |
|  | 4.3. Are the measurements appropriate? |  |  |  |  |
|  | 4.4. Is the risk of nonresponse bias low? |  |  |  |  |
|  | 4.5. Is the statistical analysis appropriate to answer the research question? |  |  |  |  |
| 5. Mixed methods | 5.1. Is there an adequate rationale for using a mixed methods design to address the research question? |  |  |  |  |
|  | 5.2. Are the different components of the study effectively integrated to answer the research question? |  |  |  |  |
|  | 5.3. Are the outputs of the integration of qualitative and quantitative components adequately interpreted? |  |  |  |  |
|  | 5.4. Are divergences and inconsistencies between quantitative and qualitative results adequately addressed? |  |  |  |  |
|  | 5.5. Do the different components of the study adhere to the quality criteria of each tradition of the methods involved? |  |  |  |  |

| [25] Kontos, P. C., Mitchell, G. J., Mistry, B., & Ballon, B. (2010). Using drama to improve person-centred dementia care. Int J Older People Nurs, 5(2), 159-168. https://doi.org/10.1111/j.1748-3743.2010.00221.x | | | | | |
| --- | --- | --- | --- | --- | --- |
|  | | | | | |
| **Category of study designs** | **Methodological quality criteria** | **Responses** | | | |
|  |  | Yes | No | Can’t tell | Comments |
| Screening questions  (for all types) | S1. Are there clear research questions? | x |  |  | *Formulated as research objective:*  The purpose of this study is to discuss the qualitative evaluation of the effectiveness of the drama-based components of the intervention. |
|  | S2. Do the collected data allow to address the research questions? |  | x |  | *However, it remains questionable to what extent effectiveness can be captured by qualitative data collection and analysis methods.* |
|  | *Further appraisal may not be feasible or appropriate when the answer is ‘No’ or ‘Can’t tell’ to one or both screening questions.* | | | | |
| 1. Qualitative | 1.1. Is the qualitative approach appropriate to answer the research question? |  |  |  |  |
|  | 1.2. Are the qualitative data collection methods adequate to address the research question? |  |  |  |  |
|  | 1.3. Are the findings adequately derived from the data? |  |  |  |  |
|  | 1.4. Is the interpretation of results sufficiently substantiated by data? |  |  |  |  |
|  | 1.5. Is there coherence between qualitative data sources, collection, analysis and interpretation? |  |  |  |  |
| 2. Quantitative randomized controlled trials | 2.1. Is randomization appropriately performed? |  |  |  |  |
|  | 2.2. Are the groups comparable at baseline? |  |  |  |  |
|  | 2.3. Are there complete outcome data? |  |  |  |  |
|  | 2.4. Are outcome assessors blinded to the intervention provided? |  |  |  |  |
|  | 2.5 Did the participants adhere to the assigned intervention? |  |  |  |  |
| 3. Quantitative non-randomized | 3.1. Are the participants representative of the target population? |  |  |  |  |
|  | 3.2. Are measurements appropriate regarding both the outcome and intervention (or exposure)? |  |  |  |  |
|  | 3.3. Are there complete outcome data? |  |  |  |  |
|  | 3.4. Are the confounders accounted for in the design and analysis? |  |  |  |  |
|  | 3.5. During the study period, is the intervention administered (or exposure occurred) as intended? |  |  |  |  |
| 4. Quantitative descriptive | 4.1. Is the sampling strategy relevant to address the research question? |  |  |  |  |
|  | 4.2. Is the sample representative of the target population? |  |  |  |  |
|  | 4.3. Are the measurements appropriate? |  |  |  |  |
|  | 4.4. Is the risk of nonresponse bias low? |  |  |  |  |
|  | 4.5. Is the statistical analysis appropriate to answer the research question? |  |  |  |  |
| 5. Mixed methods | 5.1. Is there an adequate rationale for using a mixed methods design to address the research question? |  |  |  |  |
|  | 5.2. Are the different components of the study effectively integrated to answer the research question? |  |  |  |  |
|  | 5.3. Are the outputs of the integration of qualitative and quantitative components adequately interpreted? |  |  |  |  |
|  | 5.4. Are divergences and inconsistencies between quantitative and qualitative results adequately addressed? |  |  |  |  |
|  | 5.5. Do the different components of the study adhere to the quality criteria of each tradition of the methods involved? |  |  |  |  |

| [26] Leah V, Combes J, McMillan M, Russell L, McCune K. Experiences of using simulation in dementia education. Nurs Older People. 2017;29(8):27-34. | | | | | |
| --- | --- | --- | --- | --- | --- |
|  | | | | | |
| **Category of study designs** | **Methodological quality criteria** | **Responses** | | | |
|  |  | Yes | No | Can’t tell | Comments |
| Screening questions  (for all types) | S1. Are there clear research questions? | x |  |  | *Formulation as research objectives:*   - How applicable and acceptable the design was to the participants. - To what extent simulation training met the participants’ needs in terms of developing their confidence in supporting people with dementia who were distressed. |
|  | S2. Do the collected data allow to address the research questions? | x |  |  | *Data collected and analysed reflect the research objectives.* |
|  | *Further appraisal may not be feasible or appropriate when the answer is ‘No’ or ‘Can’t tell’ to one or both screening questions.* | | | | |
| 1. Qualitative | 1.1. Is the qualitative approach appropriate to answer the research question? |  |  | x | *In principle, qualitative design is capable of achieving the research objectives. However, no rationale or justification for its use within the study report was given.* |
|  | 1.2. Are the qualitative data collection methods adequate to address the research question? |  |  | x | *There is a lack of information to answer this question.* |
|  | 1.3. Are the findings adequately derived from the data? |  |  | x | *There is a lack of information to answer this question.* |
|  | 1.4. Is the interpretation of results sufficiently substantiated by data? |  |  | x | *There is a lack of information to answer this question.* |
|  | 1.5. Is there coherence between qualitative data sources, collection, analysis and interpretation? |  |  | x | *There is a lack of information to answer this question.* |
| 2. Quantitative randomized controlled trials | 2.1. Is randomization appropriately performed? |  |  |  |  |
|  | 2.2. Are the groups comparable at baseline? |  |  |  |  |
|  | 2.3. Are there complete outcome data? |  |  |  |  |
|  | 2.4. Are outcome assessors blinded to the intervention provided? |  |  |  |  |
|  | 2.5 Did the participants adhere to the assigned intervention? |  |  |  |  |
| 3. Quantitative non-randomized | 3.1. Are the participants representative of the target population? |  |  |  |  |
|  | 3.2. Are measurements appropriate regarding both the outcome and intervention (or exposure)? |  |  |  |  |
|  | 3.3. Are there complete outcome data? |  |  |  |  |
|  | 3.4. Are the confounders accounted for in the design and analysis? |  |  |  |  |
|  | 3.5. During the study period, is the intervention administered (or exposure occurred) as intended? |  |  |  |  |
| 4. Quantitative descriptive | 4.1. Is the sampling strategy relevant to address the research question? |  |  |  |  |
|  | 4.2. Is the sample representative of the target population? |  |  |  |  |
|  | 4.3. Are the measurements appropriate? |  |  |  |  |
|  | 4.4. Is the risk of nonresponse bias low? |  |  |  |  |
|  | 4.5. Is the statistical analysis appropriate to answer the research question? |  |  |  |  |
| 5. Mixed methods | 5.1. Is there an adequate rationale for using a mixed methods design to address the research question? |  |  |  |  |
|  | 5.2. Are the different components of the study effectively integrated to answer the research question? |  |  |  |  |
|  | 5.3. Are the outputs of the integration of qualitative and quantitative components adequately interpreted? |  |  |  |  |
|  | 5.4. Are divergences and inconsistencies between quantitative and qualitative results adequately addressed? |  |  |  |  |
|  | 5.5. Do the different components of the study adhere to the quality criteria of each tradition of the methods involved? |  |  |  |  |

| [27] Lorio AK, Gore JB, Warthen L, Housley SN, Burgess EO. Teaching dementia care to physical therapy doctoral students: A multimodal experiential learning approach. Gerontol Geriatr Educ. 2016. | | | | | |
| --- | --- | --- | --- | --- | --- |
|  | | | | | |
| **Category of study designs** | **Methodological quality criteria** | **Responses** | | | |
|  |  | Yes | No | Can’t tell | Comments |
| Screening questions  (for all types) | S1. Are there clear research questions? | x |  |  | *Clearly formulated research objective:*  To describe a physical therapy program implemented a 12-hour multimodal experiential learning module designed   - to educate their students on the challenges associated with dementia, - to increase knowledge and confidence when treating these patients. |
|  | S2. Do the collected data allow to address the research questions? | x |  |  | *Yes, statements on the correlation between intervention and outcomes can be made.* |
|  | *Further appraisal may not be feasible or appropriate when the answer is ‘No’ or ‘Can’t tell’ to one or both screening questions.* | | | | |
| 1. Qualitative | 1.1. Is the qualitative approach appropriate to answer the research question? |  |  |  |  |
|  | 1.2. Are the qualitative data collection methods adequate to address the research question? |  |  |  |  |
|  | 1.3. Are the findings adequately derived from the data? |  |  |  |  |
|  | 1.4. Is the interpretation of results sufficiently substantiated by data? |  |  |  |  |
|  | 1.5. Is there coherence between qualitative data sources, collection, analysis and interpretation? |  |  |  |  |
| 2. Quantitative randomized controlled trials | 2.1. Is randomization appropriately performed? |  |  |  |  |
|  | 2.2. Are the groups comparable at baseline? |  |  |  |  |
|  | 2.3. Are there complete outcome data? |  |  |  |  |
|  | 2.4. Are outcome assessors blinded to the intervention provided? |  |  |  |  |
|  | 2.5 Did the participants adhere to the assigned intervention? |  |  |  |  |
| 3. Quantitative non-randomized | 3.1. Are the participants representative of the target population? |  |  | x | Thirty-one adult doctor of physical therapy (DPT) students, from the Department of  Physical Therapy at Georgia State University participated in this multimodal experiential  learning module as part of the 3rd-year DPT geriatrics course. This group includes 14  males and 17 females. There are 26 Whites, one African American, two Asians, and one Hispanic represented in the class. The class consists of 25 students between ages 22 and 29, three students between the ages of 30 and 39, and one student older than age 40.  *This does not provide a basis for extending the results to the entire group of physical therapy students.* |
|  | 3.2. Are measurements appropriate regarding both the outcome and intervention (or exposure)? | x |  |  | *The variables to be measured were clearly defined and therefore suitable for answering the research objective. The instruments used were validated instruments: CODE; KITE; and VDT Pre-Post-Test by Beville.* |
|  | 3.3. Are there complete outcome data? | x |  |  | *Participants included in the intervention: n = 31.*  *Participants included in analysis: Included all participants (pre n = 31 and post n = 30).* |
|  | 3.4. Are the confounders accounted for in the design and analysis? |  | x |  | *No methods to control for confounding factors were applied.* |
|  | 3.5. During the study period, is the intervention administered (or exposure occurred) as intended? | x |  |  | *There are no changes in exposure status from the intended intervention.* |
| 4. Quantitative descriptive | 4.1. Is the sampling strategy relevant to address the research question? |  |  |  |  |
|  | 4.2. Is the sample representative of the target population? |  |  |  |  |
|  | 4.3. Are the measurements appropriate? |  |  |  |  |
|  | 4.4. Is the risk of nonresponse bias low? |  |  |  |  |
|  | 4.5. Is the statistical analysis appropriate to answer the research question? |  |  |  |  |
| 5. Mixed methods | 5.1. Is there an adequate rationale for using a mixed methods design to address the research question? |  |  |  |  |
|  | 5.2. Are the different components of the study effectively integrated to answer the research question? |  |  |  |  |
|  | 5.3. Are the outputs of the integration of qualitative and quantitative components adequately interpreted? |  |  |  |  |
|  | 5.4. Are divergences and inconsistencies between quantitative and qualitative results adequately addressed? |  |  |  |  |
|  | 5.5. Do the different components of the study adhere to the quality criteria of each tradition of the methods involved? |  |  |  |  |

| [28] Maharaj T. Live Model Simulation: Improving Nursing Students' Attitudes and Knowledge of Alzheimer's Disease: The University of Texas at Tyler; 2015. | | | | | |
| --- | --- | --- | --- | --- | --- |
|  | | | | | |
| **Category of study designs** | **Methodological quality criteria** | **Responses** | | | |
|  |  | Yes | No | Can’t tell | Comments |
| Screening questions  (for all types) | S1. Are there clear research questions? | x |  |  | *Formulated as research objective (Concretisation of the objective possible: control intervention):*   - To determine whether live-model simulation used as a teaching modality could improve students’ knowledge of AD and their attitudes toward older adults with ADRD. |
|  | S2. Do the collected data allow to address the research questions? | x |  |  | *Yes, statements on the causality between intervention and outcomes can be made via an RCT.* |
|  | *Further appraisal may not be feasible or appropriate when the answer is ‘No’ or ‘Can’t tell’ to one or both screening questions.* | | | | |
| 1. Qualitative | 1.1. Is the qualitative approach appropriate to answer the research question? |  |  |  |  |
|  | 1.2. Are the qualitative data collection methods adequate to address the research question? |  |  |  |  |
|  | 1.3. Are the findings adequately derived from the data? |  |  |  |  |
|  | 1.4. Is the interpretation of results sufficiently substantiated by data? |  |  |  |  |
|  | 1.5. Is there coherence between qualitative data sources, collection, analysis and interpretation? |  |  |  |  |
| 2. Quantitative randomized controlled trials | 2.1. Is randomization appropriately performed? |  |  | x | Students were randomized to intervention (AD simulation and role-play) or control (non-AD simulation and role-play). Randomization was accomplished by using a table of random numbers. Based on the randomization students were assigned to non-AD (CHF) patient care or AD (Geri) patient care in their simulation lab experience. Just prior to the simulation experience, students were given a piece of paper with a code (AD? or CHF) designating control or intervention group; they wrote their de-identifying password on the back of it, then placed the paper in an envelope which was later matched with pre and post-test data.  *There is a lack of significant information on how the randomisation table was created, how the allocation was consequently carried out, and whether the randomisation was carried out concealed.* |
|  | 2.2. Are the groups comparable at baseline? | x |  |  | *There is no statistically significant indications of imbalance in the baseline data.* |
|  | 2.3. Are there complete outcome data? | x |  |  | *The numbers between baseline and post surveys are the same. There is no evidence of missing data sets.* |
|  | 2.4. Are outcome assessors blinded to the intervention provided? |  |  | x | *Information is missing.* |
|  | 2.5 Did the participants adhere to the assigned intervention? |  |  | x | *Information is missing.* |
| 3. Quantitative non-randomized | 3.1. Are the participants representative of the target population? |  |  |  |  |
|  | 3.2. Are measurements appropriate regarding both the outcome and intervention (or exposure)? |  |  |  |  |
|  | 3.3. Are there complete outcome data? |  |  |  |  |
|  | 3.4. Are the confounders accounted for in the design and analysis? |  |  |  |  |
|  | 3.5. During the study period, is the intervention administered (or exposure occurred) as intended? |  |  |  |  |
| 4. Quantitative descriptive | 4.1. Is the sampling strategy relevant to address the research question? |  |  |  |  |
|  | 4.2. Is the sample representative of the target population? |  |  |  |  |
|  | 4.3. Are the measurements appropriate? |  |  |  |  |
|  | 4.4. Is the risk of nonresponse bias low? |  |  |  |  |
|  | 4.5. Is the statistical analysis appropriate to answer the research question? |  |  |  |  |
| 5. Mixed methods | 5.1. Is there an adequate rationale for using a mixed methods design to address the research question? |  |  |  |  |
|  | 5.2. Are the different components of the study effectively integrated to answer the research question? |  |  |  |  |
|  | 5.3. Are the outputs of the integration of qualitative and quantitative components adequately interpreted? |  |  |  |  |
|  | 5.4. Are divergences and inconsistencies between quantitative and qualitative results adequately addressed? |  |  |  |  |
|  | 5.5. Do the different components of the study adhere to the quality criteria of each tradition of the methods involved? |  |  |  |  |

| [29] Maskeliūnas R, Damaševičius R, Lethin C, Paulauskas A, Esposito A, Catena M, et al. Serious Game iDO: Towards Better Education in Dementia Care. Information. 2019;10(11). | | | | | |
| --- | --- | --- | --- | --- | --- |
|  | | | | | |
| **Category of study designs** | **Methodological quality criteria** | **Responses** | | | |
|  |  | Yes | No | Can’t tell | Comments |
| Screening questions  (for all types) | S1. Are there clear research questions? | x |  |  | *Formulated as research objectives (not clear and poorly tailored):*   - to design an interactive mobile serious game aiming to allow caregivers to manage, a series of real world-based scenarios for dementia care and learn how to apply the problem-solving activities virtually developed to their daily routines, - to elaborate on our previous work and experience presented in reference. |
|  | S2. Do the collected data allow to address the research questions? |  | x |  | *The data from the pre-post design may provide preliminary indications but may not fully achieve the research objective.* |
|  | *Further appraisal may not be feasible or appropriate when the answer is ‘No’ or ‘Can’t tell’ to one or both screening questions.* | | | | |
| 1. Qualitative | 1.1. Is the qualitative approach appropriate to answer the research question? |  |  |  |  |
|  | 1.2. Are the qualitative data collection methods adequate to address the research question? |  |  |  |  |
|  | 1.3. Are the findings adequately derived from the data? |  |  |  |  |
|  | 1.4. Is the interpretation of results sufficiently substantiated by data? |  |  |  |  |
|  | 1.5. Is there coherence between qualitative data sources, collection, analysis and interpretation? |  |  |  |  |
| 2. Quantitative randomized controlled trials | 2.1. Is randomization appropriately performed? |  |  |  |  |
|  | 2.2. Are the groups comparable at baseline? |  |  |  |  |
|  | 2.3. Are there complete outcome data? |  |  |  |  |
|  | 2.4. Are outcome assessors blinded to the intervention provided? |  |  |  |  |
|  | 2.5 Did the participants adhere to the assigned intervention? |  |  |  |  |
| 3. Quantitative non-randomized | 3.1. Are the participants representative of the target population? |  |  | x | *The sample consisted of professionals (social care workers), people with early signs of dementia and informal caregivers. It is not stated how many participants were included in the intervention. Taken all together, this does not provide a basis for extrapolating the results to the entire group of informal caregivers and professionals.* |
|  | 3.2. Are measurements appropriate regarding both the outcome and intervention (or exposure)? | x |  |  | *The variables to be measured were clearly defined and therefore suitable for answering the research objective. The instruments used were validated instruments: DAS, GDS, SUS.* |
|  | 3.3. Are there complete outcome data? |  |  | x | *Participants included in the intervention: not clearly stated.*  *Participants included in analysis: n = 62 (Professionals: n = 12; Informal caregivers: n = 36; People with dementia: n = 14).* |
|  | 3.4. Are the confounders accounted for in the design and analysis? |  | x |  | *A suitable method to control for confounding factors was not described.* |
|  | 3.5. During the study period, is the intervention administered (or exposure occurred) as intended? |  |  | x | *No statement can be made because the number of participants included in the intervention is not clear.* |
| 4. Quantitative descriptive | 4.1. Is the sampling strategy relevant to address the research question? |  |  |  |  |
|  | 4.2. Is the sample representative of the target population? |  |  |  |  |
|  | 4.3. Are the measurements appropriate? |  |  |  |  |
|  | 4.4. Is the risk of nonresponse bias low? |  |  |  |  |
|  | 4.5. Is the statistical analysis appropriate to answer the research question? |  |  |  |  |
| 5. Mixed methods | 5.1. Is there an adequate rationale for using a mixed methods design to address the research question? |  |  |  |  |
|  | 5.2. Are the different components of the study effectively integrated to answer the research question? |  |  |  |  |
|  | 5.3. Are the outputs of the integration of qualitative and quantitative components adequately interpreted? |  |  |  |  |
|  | 5.4. Are divergences and inconsistencies between quantitative and qualitative results adequately addressed? |  |  |  |  |
|  | 5.5. Do the different components of the study adhere to the quality criteria of each tradition of the methods involved? |  |  |  |  |

| [30] Mastel-Smith B, Kimzey M, Zhaomin H. Dementia Care Education for Nursing Students. Journal of Nursing Education. 2019;58(3):136-43. | | | | | |
| --- | --- | --- | --- | --- | --- |
|  | | | | | |
| **Category of study designs** | **Methodological quality criteria** | **Responses** | | | |
|  |  | Yes | No | Can’t tell | Comments |
| Screening questions  (for all types) | S1. Are there clear research questions? | x |  |  | *Clearly formulated as research objective:*   - To learn whether receiving the De­mentia Care Bootcamp plus a dementia-specific clinical experi­ence had a greater influence on baccalaureate nursing students’ attitudes toward dementia, empathy, dementia knowledge, and confidence for dementia care, compared with students who re­ceived only the Dementia Care Bootcamp. |
|  | S2. Do the collected data allow to address the research questions? | x |  |  | *The data reflect the objective and thus answer the research question.* |
|  | *Further appraisal may not be feasible or appropriate when the answer is ‘No’ or ‘Can’t tell’ to one or both screening questions.* | | | | |
| 1. Qualitative | 1.1. Is the qualitative approach appropriate to answer the research question? |  |  |  |  |
|  | 1.2. Are the qualitative data collection methods adequate to address the research question? |  |  |  |  |
|  | 1.3. Are the findings adequately derived from the data? |  |  |  |  |
|  | 1.4. Is the interpretation of results sufficiently substantiated by data? |  |  |  |  |
|  | 1.5. Is there coherence between qualitative data sources, collection, analysis and interpretation? |  |  |  |  |
| 2. Quantitative randomized controlled trials | 2.1. Is randomization appropriately performed? |  |  |  |  |
|  | 2.2. Are the groups comparable at baseline? |  |  |  |  |
|  | 2.3. Are there complete outcome data? |  |  |  |  |
|  | 2.4. Are outcome assessors blinded to the intervention provided? |  |  |  |  |
|  | 2.5 Did the participants adhere to the assigned intervention? |  |  |  |  |
| 3. Quantitative non-randomized | 3.1. Are the participants representative of the target population? |  |  | x | A total of 100 undergraduate nursing students enrolled in the course NURS3513, Psychiatric Mental Health Nursing, dur­ing the spring of 2018 participated in this study. Based on an a priori power analysis and findings from Elvish et al. (2014), this sample provided sufficient statistical power at .8, using a significance of .05, and reported effect sizes of r = -0.56 for confidence in dementia care (large effect) and r = -0.44 for de­mentia knowledge (medium to large effect).  *Sufficient sample size available, but it remains unclear to whom the intervention is targeted.* |
|  | 3.2. Are measurements appropriate regarding both the outcome and intervention (or exposure)? | x |  |  | *The variables to be measured were clearly defined and therefore suitable for answering the research objective. The instruments used were validated and reliable instruments:*   - *Knowledge of dementia care: Dementia Knowledge Assessment Tool Version 2 (DKAT2),* - *Attitude: General perceptions of persons with AD and related dementias & feelings toward this population: Dementia Attitudes Scale (DAS),* - *Empathy: Interpersonal Reactivity Index (IRI),* - *Confidence in working with people with dementia: Confidence in Dementia Scale (CODE),* - *Fantasy/Perspective Taking/Empathic Concern/Personal Distress (IRI Subscales).* |
|  | 3.3. Are there complete outcome data? | x |  |  | *Participants included in the intervention: n = 100.*  *Participants included in analysis: n = 100.* |
|  | 3.4. Are the confounders accounted for in the design and analysis? | x |  |  | The pre- and post-test data were then matched and merged based on the independent variable created for further analyses. |
|  | 3.5. During the study period, is the intervention administered (or exposure occurred) as intended? |  |  | x | *Unclear because of missing information.* |
| 4. Quantitative descriptive | 4.1. Is the sampling strategy relevant to address the research question? |  |  |  |  |
|  | 4.2. Is the sample representative of the target population? |  |  |  |  |
|  | 4.3. Are the measurements appropriate? |  |  |  |  |
|  | 4.4. Is the risk of nonresponse bias low? |  |  |  |  |
|  | 4.5. Is the statistical analysis appropriate to answer the research question? |  |  |  |  |
| 5. Mixed methods | 5.1. Is there an adequate rationale for using a mixed methods design to address the research question? |  |  |  |  |
|  | 5.2. Are the different components of the study effectively integrated to answer the research question? |  |  |  |  |
|  | 5.3. Are the outputs of the integration of qualitative and quantitative components adequately interpreted? |  |  |  |  |
|  | 5.4. Are divergences and inconsistencies between quantitative and qualitative results adequately addressed? |  |  |  |  |
|  | 5.5. Do the different components of the study adhere to the quality criteria of each tradition of the methods involved? |  |  |  |  |

| [31] Mastel-Smith B, Kimzey M, Garner J, Shoair OA, Stocks E, Wallace T. Dementia care boot camp: interprofessional education for healthcare students. Journal of Interprofessional Care. 2019. | | | | | |
| --- | --- | --- | --- | --- | --- |
|  | | | | | |
| **Category of study designs** | **Methodological quality criteria** | **Responses** | | | |
|  |  | Yes | No | Can’t tell | Comments |
| Screening questions  (for all types) | S1. Are there clear research questions? | x |  |  | *Clearly formulated as research objective(s), hypotheses, and questions. A statement on the intention of integrating the results (in the sense of an Mixed-Methods approach) is missing:*  The purposes of this mixed methods study were to:   - a) examine the effect of a 16-h program on pharmacy, nursing, and occupational therapy students’ dementia knowledge and attitudes toward people with dementia, confidence for dementia care and empathy; - b) understand the relationships among outcome variables so that future efforts might focus on activities which have the greatest effect on students’ confidence to care for people with dementia; and - c) elicit students’ responses to interprofessional education using team-based learning   Quantitative hypotheses: H1: Nursing, occupational therapy assistant, pharmacy, and psychology students who participate in a 16-h IPE program will report improved attitudes toward people with dementia, dementia knowledge, confidence for  dementia care and empathy at the end of the program and three months later. H2: There will be a significant, positive relationship among dementia knowledge, dementia attitudes, confidence in dementia care and empathy.  Qualitative research question: What are students’ experiences with the dementia care Bootcamp? |
|  | S2. Do the collected data allow to address the research questions? | x |  |  | *Data collection included focus groups and quantitative questionnaires to cover the research objectives.* |
|  | *Further appraisal may not be feasible or appropriate when the answer is ‘No’ or ‘Can’t tell’ to one or both screening questions.* | | | | |
| 1. Qualitative | 1.1. Is the qualitative approach appropriate to answer the research question? | x |  |  | Krueger and Casey’s (2000) categories of focus group questions were employed, that is; opening, introductory, transition, key and ending questions. An interview guide was used and included questions about what went well, what needed improvement, from which activities did they learn new information, what was it about the activity that facilitated learning, barriers to learning, and suggestions for future programs. |
|  | 1.2. Are the qualitative data collection methods adequate to address the research question? | x |  |  | *Yes, see above.* |
|  | 1.3. Are the findings adequately derived from the data? | x |  |  | Colaizzi’s (1978) seven-step method guided qualitative data analysis. Three researchers individually a) read and reread transcripts, b) identified significant statements and c) meanings, d) categorized meanings into clusters of themes and  validated with transcripts, and e) integrated clusters into a description of the phenomenon. Three participants f) reviewed the written narrative and theoretical model and g) their suggestions were incorporated into a final description. Researchers set  aside previous opinions, took notes during focus groups and created a codebook to further ensure credibility. |
|  | 1.4. Is the interpretation of results sufficiently substantiated by data? | x |  |  | *The data interpretation was underpinned by quotes that correctly describe the respective themes (see also table 7).* |
|  | 1.5. Is there coherence between qualitative data sources, collection, analysis and interpretation? | x |  |  | *The intended research goals are reflected in the identified themes as well as in the data interpretation.* |
| 2. Quantitative randomized controlled trials | 2.1. Is randomization appropriately performed? |  |  |  |  |
|  | 2.2. Are the groups comparable at baseline? |  |  |  |  |
|  | 2.3. Are there complete outcome data? |  |  |  |  |
|  | 2.4. Are outcome assessors blinded to the intervention provided? |  |  |  |  |
|  | 2.5 Did the participants adhere to the assigned intervention? |  |  |  |  |
| 3. Quantitative non-randomized | 3.1. Are the participants representative of the target population? | x |  |  | We aimed to recruit 20 students from each discipline (nursing, occupational therapy assistant, pharmacy and psychology) for a total 80 participants.  Learners were strategically placed in interprofessional teams consisting of nursing, pharmacy, occupational therapy assistant, and psychology students.  *There is evidence that the included patients correspond to the target population of the intervention.* |
|  | 3.2. Are measurements appropriate regarding both the outcome and intervention (or exposure)? | x |  |  | *The variables to be measured were clearly defined and therefore suitable for answering the research objective. The instruments used were validated instruments: DKAT2, DAS, IRI, CODE* |
|  | 3.3. Are there complete outcome data? |  |  | x | *Participants included in the intervention: not clearly stated*  *Participants included in analysis: n = 43 (nursing: n = 19; occupational therapy assistants: n = 13; pharmacy: n = 3; psychology: n = 8)*  *Lack of information.* |
|  | 3.4. Are the confounders accounted for in the design and analysis? |  | x |  | As for validity, at least half of the students reported previous interactions with people with dementia, a possible confounding variable.  *No methods to control for confounding factors were applied.* |
|  | 3.5. During the study period, is the intervention administered (or exposure occurred) as intended? | x |  |  | *There are no changes in exposure status from the intended intervention reported.* |
| 4. Quantitative descriptive | 4.1. Is the sampling strategy relevant to address the research question? |  |  |  |  |
|  | 4.2. Is the sample representative of the target population? |  |  |  |  |
|  | 4.3. Are the measurements appropriate? |  |  |  |  |
|  | 4.4. Is the risk of nonresponse bias low? |  |  |  |  |
|  | 4.5. Is the statistical analysis appropriate to answer the research question? |  |  |  |  |
| 5. Mixed methods | 5.1. Is there an adequate rationale for using a mixed methods design to address the research question? |  |  | x | An embedded mixed methods design was used in which qualitative focus group data were embedded within a major quasiexperimental pre-post design. A pre-post design lacks the rigor of randomization; however, we felt that all students should receive the entire program (in contrast to a comparison group that completed an online module, for example). The quantitative data was used to test the theory that predicts that education will positively influence health professional students’ dementia knowledge, attitudes toward people with dementia, empathy and confidence for dementia care at a state university and junior college in the southern U.S. The qualitative data were embedded  in the larger intervention trial for the purpose of explaining the quantitative data and enhancing future education efforts.  *The rationale for using the MMD remains unclear.* |
|  | 5.2. Are the different components of the study effectively integrated to answer the research question? |  | x |  | *The results were presented separately.* |
|  | 5.3. Are the outputs of the integration of qualitative and quantitative components adequately interpreted? |  | x |  | *See above.* |
|  | 5.4. Are divergences and inconsistencies between quantitative and qualitative results adequately addressed? |  | x |  | *See above* |
|  | 5.5. Do the different components of the study adhere to the quality criteria of each tradition of the methods involved? | x |  |  | *The qualitative data strand is of high quality, whereas the quantitative data strand is of moderate quality.* |

| [32] Meyer K, James D, Amezaga B, White C. Simulation learning to train healthcare students in person-centered dementia care. Gerontology & Geriatrics Education. 2020. | | | | | |
| --- | --- | --- | --- | --- | --- |
|  | | | | | |
| **Category of study designs** | **Methodological quality criteria** | **Responses** | | | |
|  |  | Yes | No | Can’t tell | Comments |
| Screening questions  (for all types) | S1. Are there clear research questions? | x |  |  | *Formulated as research objective and clearly stated research question:*   - to examine *how* a simulation training program may prepare healthcare trainees to treat individuals living with dementia. - Research question: How does participation in a dementia simulation program contribute to healthcare students’ abilities to deliver person-centered care to individuals living with dementia? |
|  | S2. Do the collected data allow to address the research questions? | x |  |  | *The data reflect the objective and thus answer the research question.* |
|  | *Further appraisal may not be feasible or appropriate when the answer is ‘No’ or ‘Can’t tell’ to one or both screening questions.* | | | | |
| 1. Qualitative | 1.1. Is the qualitative approach appropriate to answer the research question? | x |  |  | However, these questions do not indicate how the tour improves understanding of dementia nor changes in participants’ abilities to deliver high quality healthcare to individuals living with dementia. Qualitative data can provide more nuanced information as to how dementia simulation learning improves healthcare students’ understanding of what it is like to live with dementia and how the program may affect delivery of care to people living with dementia. Using qualitative methods has been recommended to better understand how simulation learning increases participant insight and empathy (Mosher-Ashley, 1996). In this study, we build on previous evaluations of VDT by using multiple sources of data, including student reflection papers and in-depth interviews with students and faculty (Beville, 2002; Werner et al., 2014). In doing so, this study will respond to calls for additional research on the benefits of provider training on dementia care (Weiss et al., 2017). |
|  | 1.2. Are the qualitative data collection methods adequate to address the research question? | x |  |  | *Consistency with objective apparent; correct to address research question:*  The researchers used a thematic analytic method to answer the research question and  applied this approach to two sources of data: 1) in-depth, semi-structured one-on-one  interviews with students who completed VDT within the previous 6-months and teaching faculty who included VDT into their courses, as well as 2) student reflection papers completed within 1-week of participating in VDT. Whereas interview methods were selected to allow examination of how students applied lessons learned from VDT given a time lag between program participation and data collection, document review of reflection papers enabled examination of students’ more immediate VDT experiences given a shorter recall period since participating in VDT. |
|  | 1.3. Are the findings adequately derived from the data? | x |  |  | We applied a thematic analysis to the data, as this method allowed us the flexibility to code data both inductively and deductively (Braun & Clarke, 2006). After reviewing transcribed interviews and reflection papers, DJ and KM collaboratively devised a coding tree and definitions. Once members of the study team provided feedback on the initial coding tree, KM completed the first round of coding. Coding was completed using color-coded text in Microsoft Word documents corresponding to codes. Text excerpts were then organized by code in another Microsoft Word document such that excerpts falling under the same code could be evaluated for internal consistency. After completing the first round of coding, DJ and KM met to review each transcript and reflection paper and to discuss the appropriateness of the code applications and to identify emerging codes. All coding discrepancies were discussed until agreement was reached. A third round of coding was conducted to update all data files with codes that were added. (Analysis during the third round of coding occurred in NVivo for Mac to better accommodate the additional codes.) Results were discussed with the study team during a video-phone meeting. Each author had access to the drafted results section, all data, the coding tree, and a list of text excerpts included under each code. Table 3  summarizes the steps taken by the research team to ensure study rigor and trustworthiness (Nowell, Norris, White, & Moules, 2017).  *There is no evidence to suggest otherwise.* |
|  | 1.4. Is the interpretation of results sufficiently substantiated by data? | x |  |  | *The data interpretation was underpinned by quotes that correctly describe the respective topics.* |
|  | 1.5. Is there coherence between qualitative data sources, collection, analysis and interpretation? | x |  |  | *The intended research goals are reflected in the identified themes as well as in the data interpretation.* |
| 2. Quantitative randomized controlled trials | 2.1. Is randomization appropriately performed? |  |  |  |  |
|  | 2.2. Are the groups comparable at baseline? |  |  |  |  |
|  | 2.3. Are there complete outcome data? |  |  |  |  |
|  | 2.4. Are outcome assessors blinded to the intervention provided? |  |  |  |  |
|  | 2.5 Did the participants adhere to the assigned intervention? |  |  |  |  |
| 3. Quantitative non-randomized | 3.1. Are the participants representative of the target population? |  |  |  |  |
|  | 3.2. Are measurements appropriate regarding both the outcome and intervention (or exposure)? |  |  |  |  |
|  | 3.3. Are there complete outcome data? |  |  |  |  |
|  | 3.4. Are the confounders accounted for in the design and analysis? |  |  |  |  |
|  | 3.5. During the study period, is the intervention administered (or exposure occurred) as intended? |  |  |  |  |
| 4. Quantitative descriptive | 4.1. Is the sampling strategy relevant to address the research question? |  |  |  |  |
|  | 4.2. Is the sample representative of the target population? |  |  |  |  |
|  | 4.3. Are the measurements appropriate? |  |  |  |  |
|  | 4.4. Is the risk of nonresponse bias low? |  |  |  |  |
|  | 4.5. Is the statistical analysis appropriate to answer the research question? |  |  |  |  |
| 5. Mixed methods | 5.1. Is there an adequate rationale for using a mixed methods design to address the research question? |  |  |  |  |
|  | 5.2. Are the different components of the study effectively integrated to answer the research question? |  |  |  |  |
|  | 5.3. Are the outputs of the integration of qualitative and quantitative components adequately interpreted? |  |  |  |  |
|  | 5.4. Are divergences and inconsistencies between quantitative and qualitative results adequately addressed? |  |  |  |  |
|  | 5.5. Do the different components of the study adhere to the quality criteria of each tradition of the methods involved? |  |  |  |  |

| [33] Peng X, Wu L, Xie X, Dai M, Wang D. Impact of Virtual Dementia Tour on empathy level of nursing students: A quasi-experimental study. Int J Nurs Sci. 2020;7(3):258-61. | | | | | |
| --- | --- | --- | --- | --- | --- |
|  | | | | | |
| **Category of study designs** | **Methodological quality criteria** | **Responses** | | | |
|  |  | Yes | No | Can’t tell | Comments |
| Screening questions  (for all types) | S1. Are there clear research questions? | x |  |  | *Clearly formulated as research objective:*  To evaluate the effect of Virtual Dementia Tour (VDT) upon nursing students’ empathy level and propose practical rationales for optimizing future dementia care. |
|  | S2. Do the collected data allow to address the research questions? | x |  |  | The level of empathy was measured by the Chinese version of Jefferson Scale of Empathy-Health Professional Students (JSE-HPS). |
|  | *Further appraisal may not be feasible or appropriate when the answer is ‘No’ or ‘Can’t tell’ to one or both screening questions.* | | | | |
| 1. Qualitative | 1.1. Is the qualitative approach appropriate to answer the research question? |  |  |  |  |
|  | 1.2. Are the qualitative data collection methods adequate to address the research question? |  |  |  |  |
|  | 1.3. Are the findings adequately derived from the data? |  |  |  |  |
|  | 1.4. Is the interpretation of results sufficiently substantiated by data? |  |  |  |  |
|  | 1.5. Is there coherence between qualitative data sources, collection, analysis and interpretation? |  |  |  |  |
| 2. Quantitative randomized controlled trials | 2.1. Is randomization appropriately performed? |  |  |  |  |
|  | 2.2. Are the groups comparable at baseline? |  |  |  |  |
|  | 2.3. Are there complete outcome data? |  |  |  |  |
|  | 2.4. Are outcome assessors blinded to the intervention provided? |  |  |  |  |
|  | 2.5 Did the participants adhere to the assigned intervention? |  |  |  |  |
| 3. Quantitative non-randomized | 3.1. Are the participants representative of the target population? |  | x |  | As a popular evidence-based study tool, VDT has been employed for boosting empathy and optimizing the delivery of care [12]. To the best of our knowledge, it has not been applied in China. With the largest population of demented patients in the world [13], China has an urgent need for training dedicated providers for dementia cares. Therefore the objective of this study was to explore the impact of VDT upon empathy of nursing students, summarize the experiences of participating in VDT training and provide rationales for optimizing future nursing practices for dementia.  *Patients included in the study only partially cover the target group of the intervention.* |
|  | 3.2. Are measurements appropriate regarding both the outcome and intervention (or exposure)? | x |  |  | A set of demographic questionnaire and JSE-HPS with a randomly generated number was distributed to each participant and completed before September 15. The participants were required to remember the random number assigned to each one.  On September 15, a pilot trial of 6 nursing students was conducted. On October 14, all participants watched the movie together. On October 16 and 17, each of them was “garbed” in the same way and took the VDT in turns on specific timepoints. From October 18 to 22, JSE-HPS was re-assessed and each questionnaire was taken back  with the assigned random number. A semi-structured interview was conducted separately. |
|  | 3.3. Are there complete outcome data? | x |  |  | *It seems that there were no dropouts of participants; also the time points of data collection by instrument were completely reported.* |
|  | 3.4. Are the confounders accounted for in the design and analysis? |  | x |  | *No methods for controlling confounding factors were described.* |
|  | 3.5. During the study period, is the intervention administered (or exposure occurred) as intended? | x |  |  | *There are no changes in exposure status from the intended intervention reported.* |
| 4. Quantitative descriptive | 4.1. Is the sampling strategy relevant to address the research question? |  |  |  |  |
|  | 4.2. Is the sample representative of the target population? |  |  |  |  |
|  | 4.3. Are the measurements appropriate? |  |  |  |  |
|  | 4.4. Is the risk of nonresponse bias low? |  |  |  |  |
|  | 4.5. Is the statistical analysis appropriate to answer the research question? |  |  |  |  |
| 5. Mixed methods | 5.1. Is there an adequate rationale for using a mixed methods design to address the research question? |  |  |  |  |
|  | 5.2. Are the different components of the study effectively integrated to answer the research question? |  |  |  |  |
|  | 5.3. Are the outputs of the integration of qualitative and quantitative components adequately interpreted? |  |  |  |  |
|  | 5.4. Are divergences and inconsistencies between quantitative and qualitative results adequately addressed? |  |  |  |  |
|  | 5.5. Do the different components of the study adhere to the quality criteria of each tradition of the methods involved? |  |  |  |  |

| [34] Prins, M., Veerbeek, M., Willemse, B. M., & Pot, A. M. (2020). Use and impact of the Alzheimer Experience: a free online media production to raise public awareness and enhance knowledge and understanding of dementia. *Aging & mental health*, *24*(6), 985-992. https://doi.org/10.1080/13607863.2019.1579781 | | | | | |
| --- | --- | --- | --- | --- | --- |
|  | | | | | |
| **Category of study designs** | **Methodological quality criteria** | **Responses** | | | |
|  |  | Yes | No | Can’t tell | Comments |
| Screening questions  (for all types) | S1. Are there clear research questions? | x |  |  | *Formulated as research objective:*  To evaluate an online media production called 'the Alzheimer Experience' (AlzExp), which was developed to raise public awareness and enhance knowledge and understanding of dementia. |
|  | S2. Do the collected data allow to address the research questions? | x |  |  | Before and after watching AlzExp, all visitors of AlzExp (February–June 2012) were asked to complete questionnaires on demographics, knowledge about dementia, attitude towards people with dementia (person-centeredness) and the use of AlzExp. |
|  | *Further appraisal may not be feasible or appropriate when the answer is ‘No’ or ‘Can’t tell’ to one or both screening questions.* | | | | |
| 1. Qualitative | 1.1. Is the qualitative approach appropriate to answer the research question? |  |  |  |  |
|  | 1.2. Are the qualitative data collection methods adequate to address the research question? |  |  |  |  |
|  | 1.3. Are the findings adequately derived from the data? |  |  |  |  |
|  | 1.4. Is the interpretation of results sufficiently substantiated by data? |  |  |  |  |
|  | 1.5. Is there coherence between qualitative data sources, collection, analysis and interpretation? |  |  |  |  |
| 2. Quantitative randomized controlled trials | 2.1. Is randomization appropriately performed? |  |  |  |  |
|  | 2.2. Are the groups comparable at baseline? |  |  |  |  |
|  | 2.3. Are there complete outcome data? |  |  |  |  |
|  | 2.4. Are outcome assessors blinded to the intervention provided? |  |  |  |  |
|  | 2.5 Did the participants adhere to the assigned intervention? |  |  |  |  |
| 3. Quantitative non-randomized | 3.1. Are the participants representative of the target population? |  | x |  | *The research objectives also included raising public awareness; however, mainly caregivers could be included.*  *It appeared that participants in the evaluation of the Alzheimer Experience were mainly people who worked with people with dementia or who had someone with*  *dementia among their acquaintances. This makes it hard to generalize the findings to the wider public.* |
|  | 3.2. Are measurements appropriate regarding both the outcome and intervention (or exposure)? | x |  |  | *The variables to be measured were clearly defined and accurately measured: Use, Impact (Knowledge, Attitudes) and thus suitable for answering the research question.*  *Some of the instruments used were self-constructed, which is to be assessed negatively. In some cases, individual items were taken from validated scales.* |
|  | 3.3. Are there complete outcome data? |  | x |  | *Out of a total of 998 people who agreed to participate in the evaluation, only 220 (22%) could be included in the analysis.*  *In total, 8374 people visited the Alzheimer Experience website during the analysis period.* |
|  | 3.4. Are the confounders accounted for in the design and analysis? |  | x |  | *No methods for controlling confounding factors were described.* |
|  | 3.5. During the study period, is the intervention administered (or exposure occurred) as intended? | x |  |  | *There are no changes in exposure status from the intended intervention.* |
| 4. Quantitative descriptive | 4.1. Is the sampling strategy relevant to address the research question? |  |  |  |  |
|  | 4.2. Is the sample representative of the target population? |  |  |  |  |
|  | 4.3. Are the measurements appropriate? |  |  |  |  |
|  | 4.4. Is the risk of nonresponse bias low? |  |  |  |  |
|  | 4.5. Is the statistical analysis appropriate to answer the research question? |  |  |  |  |
| 5. Mixed methods | 5.1. Is there an adequate rationale for using a mixed methods design to address the research question? |  |  |  |  |
|  | 5.2. Are the different components of the study effectively integrated to answer the research question? |  |  |  |  |
|  | 5.3. Are the outputs of the integration of qualitative and quantitative components adequately interpreted? |  |  |  |  |
|  | 5.4. Are divergences and inconsistencies between quantitative and qualitative results adequately addressed? |  |  |  |  |
|  | 5.5. Do the different components of the study adhere to the quality criteria of each tradition of the methods involved? |  |  |  |  |

| [35] Sharkey F, Moore K, Slater P, Hassan F. The Impact of a Virtual Reality Training Programme on Health Professional Knowledge, Understanding and Empathy in Relation to Dementia. 2019 | | | | | |
| --- | --- | --- | --- | --- | --- |
|  | | | | | |
| **Category of study designs** | **Methodological quality criteria** | **Responses** | | | |
|  |  | Yes | No | Can’t tell | Comments |
| Screening questions  (for all types) | S1. Are there clear research questions? | x |  |  | *Formulated as research objectives:*   - To explore and examine the impact of a VDT programme on participants in terms of their professional knowledge, understanding and empathy in the provision of person-centred care - The authors aimed to provide an evidence base of the impact of VDT on views and perceptions of care and provide a strong and critical evidence-base for maximising its potential based on a comprehensive analysis of the research results |
|  | S2. Do the collected data allow to address the research questions? | x |  |  | *Taken all together, the data presented reflect the objective and thus answer the research objectives.* |
|  | *Further appraisal may not be feasible or appropriate when the answer is ‘No’ or ‘Can’t tell’ to one or both screening questions.* | | | | |
| 1. Qualitative | 1.1. Is the qualitative approach appropriate to answer the research question? | x |  |  | Focus groups were undertaken to evaluate the views of the opinions of participants in the VDT® programme and to gain an insight into the perceived impact of the training on practice. A discussion schedule based on the aims and objectives of the study and a review of the literature was developed and with consent, discussions were digitally recorded. |
|  | 1.2. Are the qualitative data collection methods adequate to address the research question? | x |  |  | *Yes, see above.* |
|  | 1.3. Are the findings adequately derived from the data? | x |  |  | All digital recordings were transcribed verbatim and then subject to thematic analysis using the six-step framework advocated by Braun and Clarke (2006) which involves familiarising yourself with the data, generating initial codes, searching for themes, reviewing themes, defining and naming themes and producing the report.  *Furthermore, measures to ensure rigour were described*. |
|  | 1.4. Is the interpretation of results sufficiently substantiated by data? | x |  |  | *The data interpretation was underpinned by quotes that correctly describe the respective topics.* |
|  | 1.5. Is there coherence between qualitative data sources, collection, analysis and interpretation? | x |  |  | Five themes emerged from the thematic content analysis of the qualitative data: (1) immersive learning; (2) VDT ® experience; (3) empathy related responses; (4) authenticity and transferability and (5) suggestions for improvement.  *The intended research goals are reflected in the identified themes as well as in the data interpretation.* |
| 2. Quantitative randomized controlled trials | 2.1. Is randomization appropriately performed? |  |  |  |  |
|  | 2.2. Are the groups comparable at baseline? |  |  |  |  |
|  | 2.3. Are there complete outcome data? |  |  |  |  |
|  | 2.4. Are outcome assessors blinded to the intervention provided? |  |  |  |  |
|  | 2.5 Did the participants adhere to the assigned intervention? |  |  |  |  |
| 3. Quantitative non-randomized | 3.1. Are the participants representative of the target population? |  |  | x | Participants were drawn from across one of the five Health and Social Care Trusts in Northern Ireland that have been using the VDT® as a training tool. Participation was voluntary and open to health professionals, voluntary groups and members of the community. Over the period of two weeks, there were a total of 240 participants.  *Participants included: Health professionals, Informal Caregivers, voluntary groups, members of the community;*  *Since the target population of the intervention programme is not clearly described, it remains unclear whether the included participants represent the target group.* |
|  | 3.2. Are measurements appropriate regarding both the outcome and intervention (or exposure)? | x |  |  | The ‘Empathy and Understanding in Dementia Index’, a valid and reliable assessment tool designed to measure the impact of training on empathetic understanding and person-centred practice in dementia was used and reported to have strong psychometric properties (Slater et al. in press). It consisted of 19 items in total, 15 items, measuring empathy, understand and person-centred care. Four additional items measured experience of previous training.  *The variables to be measured were clearly defined and therefore suitable for answering the research objective.* |
|  | 3.3. Are there complete outcome data? |  | x |  | *Phase 1: n = 223 (completed questionnaires for all time points); n = 83 (3 months post intervention)* |
|  | 3.4. Are the confounders accounted for in the design and analysis? |  | x |  | *No methods to control for confounding factors were applied.* |
|  | 3.5. During the study period, is the intervention administered (or exposure occurred) as intended? | x |  |  | *There are no changes in exposure status from the intended intervention.* |
| 4. Quantitative descriptive | 4.1. Is the sampling strategy relevant to address the research question? |  |  |  |  |
|  | 4.2. Is the sample representative of the target population? |  |  |  |  |
|  | 4.3. Are the measurements appropriate? |  |  |  |  |
|  | 4.4. Is the risk of nonresponse bias low? |  |  |  |  |
|  | 4.5. Is the statistical analysis appropriate to answer the research question? |  |  |  |  |
| 5. Mixed methods | 5.1. Is there an adequate rationale for using a mixed methods design to address the research question? |  | x |  | *However, no rationale provided for using mixed methods design resp. integrating both data strands.* |
|  | 5.2. Are the different components of the study effectively integrated to answer the research question? |  | x |  | *The results were only presented side by side and not integrated.* |
|  | 5.3. Are the outputs of the integration of qualitative and quantitative components adequately interpreted? |  | x |  | *See above.* |
|  | 5.4. Are divergences and inconsistencies between quantitative and qualitative results adequately addressed? |  | x |  | *See above* |
|  | 5.5. Do the different components of the study adhere to the quality criteria of each tradition of the methods involved? | x |  |  | *Presence of medium quality, as partial studies are of medium to high quality.* |

| [36] Slater, P., Hasson, F., Gillen, P., Gallen, A., & Parlour, R. (2019). Virtual simulation training: Imaged experience of dementia. International Journal of Older People Nursing, 14(3), N.PAG-N.PAG. https://doi.org/10.1111/opn.12243 | | | | | |
| --- | --- | --- | --- | --- | --- |
|  | | | | | |
| **Category of study designs** | **Methodological quality criteria** | **Responses** | | | |
|  |  | Yes | No | Can’t tell | Comments |
| Screening questions  (for all types) | S1. Are there clear research questions? | x |  |  | *Formulated* as research objectives:   - To investigate the impact of the immersive Virtual Dementia Tour (VDT®) on empathy of health and social care staff and caregivers. - To explore the impact of an interactive training experience on moral, emotive, behavioral and cognitive elements of empathy |
|  | S2. Do the collected data allow to address the research questions? | x |  |  | Given the dearth of research exploring the impact of virtual training on the four components of empathy, a qualitative exploratory design was adopted. |
|  | *Further appraisal may not be feasible or appropriate when the answer is ‘No’ or ‘Can’t tell’ to one or both screening questions.* | | | | |
| 1. Qualitative | 1.1. Is the qualitative approach appropriate to answer the research question? | x |  |  | *See above.* |
|  | 1.2. Are the qualitative data collection methods adequate to address the research question? | x |  |  | An interview schedule, based on the aims of the study and the four component  model (Morse et al., 1992), guided by three broad aspects: (1) experiences of the VDT® programme (2) the benefits and (3) impact of the programme on practice. Probes were used to clarify the meaning of responses. A mixture of one‐to‐one face‐to‐face (undertaken in the home and Health Service Executive premises) and telephone  interviews were conducted that lasted between 15 and 35 min and with permission were digitally recorded and supplemented by field notes. |
|  | 1.3. Are the findings adequately derived from the data? |  |  | x | *Data analysis and methods for compliance with the rigour were comprehensively presented. Deductive approach (following Morse et al.) does not correspond to thematic analysis.* |
|  | 1.4. Is the interpretation of results sufficiently substantiated by data? | x |  |  | *Interpretation is underpinned by quotes.* |
|  | 1.5. Is there coherence between qualitative data sources, collection, analysis and interpretation? |  |  | x | *The intended research goals are reflected in the identified themes as well as in the data interpretation. Deductive approach (following Morse et al.) does not correspond to thematic analysis.* |
| 2. Quantitative randomized controlled trials | 2.1. Is randomization appropriately performed? |  |  |  |  |
|  | 2.2. Are the groups comparable at baseline? |  |  |  |  |
|  | 2.3. Are there complete outcome data? |  |  |  |  |
|  | 2.4. Are outcome assessors blinded to the intervention provided? |  |  |  |  |
|  | 2.5 Did the participants adhere to the assigned intervention? |  |  |  |  |
| 3. Quantitative non-randomized | 3.1. Are the participants representative of the target population? |  |  |  |  |
|  | 3.2. Are measurements appropriate regarding both the outcome and intervention (or exposure)? |  |  |  |  |
|  | 3.3. Are there complete outcome data? |  |  |  |  |
|  | 3.4. Are the confounders accounted for in the design and analysis? |  |  |  |  |
|  | 3.5. During the study period, is the intervention administered (or exposure occurred) as intended? |  |  |  |  |
| 4. Quantitative descriptive | 4.1. Is the sampling strategy relevant to address the research question? |  |  |  |  |
|  | 4.2. Is the sample representative of the target population? |  |  |  |  |
|  | 4.3. Are the measurements appropriate? |  |  |  |  |
|  | 4.4. Is the risk of nonresponse bias low? |  |  |  |  |
|  | 4.5. Is the statistical analysis appropriate to answer the research question? |  |  |  |  |
| 5. Mixed methods | 5.1. Is there an adequate rationale for using a mixed methods design to address the research question? |  |  |  |  |
|  | 5.2. Are the different components of the study effectively integrated to answer the research question? |  |  |  |  |
|  | 5.3. Are the outputs of the integration of qualitative and quantitative components adequately interpreted? |  |  |  |  |
|  | 5.4. Are divergences and inconsistencies between quantitative and qualitative results adequately addressed? |  |  |  |  |
|  | 5.5. Do the different components of the study adhere to the quality criteria of each tradition of the methods involved? |  |  |  |  |

| [37] Slater P, Hasson F, Gillen P. A research evaluation of an interactive training experience: The virtual Dementia Tour (VDT). Ulster University; 2017. | | | | | |
| --- | --- | --- | --- | --- | --- |
|  | | | | | |
| **Category of study designs** | **Methodological quality criteria** | **Responses** | | | |
|  |  | Yes | No | Can’t tell | Comments |
| Screening questions  (for all types) | S1. Are there clear research questions? | x |  |  | *Clearly formulated as research objectives:*   - To explore the experiences and perceptions of impact on the practice of health and social care staff and carers who participated in the VDTR training. - To explore the perceptions of impact of VDTR upon delivery of Dementia care in the future. - To consider participant reaction to how the VDTR programme was conducted and how this may be improved. |
|  | S2. Do the collected data allow to address the research questions? | x |  |  | Given the lack of research in this area, a qualitative research design comprising of semistructured, face-to-face and telephone interviews, was used to evaluate the perceptions of participants in the VDTR programme. |
|  | Further appraisal may not be feasible or appropriate when the answer is ‘No’ or ‘Can’t tell’ to one or both screening questions. | | | | |
| 1. Qualitative | 1.1. Is the qualitative approach appropriate to answer the research question? | x |  |  | *See above.* |
|  | 1.2. Are the qualitative data collection methods adequate to address the research question? | x |  |  | Semi-structured interviews provide a standardised structure to the interview process but provide flexibility in the phasing and ordering of the questions (van Teijlingen 2014). Telephone interviews helped provide flexibility to work with participants’ busy work schedules, and use economic and human resources effectively (Musselwhite et al. 2007) by reducing travelling, given the wide geographical spread of participants and the research team.  A short interview schedule was developed based on the aims and objectives of the study and a review of the literature (see Appendix 1). The schedule highlights three broad aspects to be addressed: (1) experiences of the VDTR programme (2) the benefits of VDTR Programme and (3) impact of the VDTR programme on practice. Probes were used to clarify the meanings of responses. |
|  | 1.3. Are the findings adequately derived from the data? | x |  |  | *Data analysis and methods for compliance with rigour have been comprehensively presented. The application can be seen in the results.* |
|  | 1.4. Is the interpretation of results sufficiently substantiated by data? | x |  |  | *Interpretation is underpinned by quotes and further examples within the text.* |
|  | 1.5. Is there coherence between qualitative data sources, collection, analysis and interpretation? | x |  |  |  |
| 2. Quantitative randomized controlled trials | 2.1. Is randomization appropriately performed? |  |  |  |  |
|  | 2.2. Are the groups comparable at baseline? |  |  |  |  |
|  | 2.3. Are there complete outcome data? |  |  |  |  |
|  | 2.4. Are outcome assessors blinded to the intervention provided? |  |  |  |  |
|  | 2.5 Did the participants adhere to the assigned intervention? |  |  |  |  |
| 3. Quantitative non-randomized | 3.1. Are the participants representative of the target population? |  |  |  |  |
|  | 3.2. Are measurements appropriate regarding both the outcome and intervention (or exposure)? |  |  |  |  |
|  | 3.3. Are there complete outcome data? |  |  |  |  |
|  | 3.4. Are the confounders accounted for in the design and analysis? |  |  |  |  |
|  | 3.5. During the study period, is the intervention administered (or exposure occurred) as intended? |  |  |  |  |
| 4. Quantitative descriptive | 4.1. Is the sampling strategy relevant to address the research question? |  |  |  |  |
|  | 4.2. Is the sample representative of the target population? |  |  |  |  |
|  | 4.3. Are the measurements appropriate? |  |  |  |  |
|  | 4.4. Is the risk of nonresponse bias low? |  |  |  |  |
|  | 4.5. Is the statistical analysis appropriate to answer the research question? |  |  |  |  |
| 5. Mixed methods | 5.1. Is there an adequate rationale for using a mixed methods design to address the research question? |  |  |  |  |
|  | 5.2. Are the different components of the study effectively integrated to answer the research question? |  |  |  |  |
|  | 5.3. Are the outputs of the integration of qualitative and quantitative components adequately interpreted? |  |  |  |  |
|  | 5.4. Are divergences and inconsistencies between quantitative and qualitative results adequately addressed? |  |  |  |  |
|  | 5.5. Do the different components of the study adhere to the quality criteria of each tradition of the methods involved? |  |  |  |  |

| [38] Slater P, Hasson F, Moore K, Sharkey F. Simulated Based Dementia Training: Impact on Empathic Understanding and Behaviour Among Professionals and Carers. Clinical Simulation in Nursing. 2021;55:43-51. | | | | | |
| --- | --- | --- | --- | --- | --- |
|  | | | | | |
| **Category of study designs** | **Methodological quality criteria** | **Responses** | | | |
|  |  | Yes | No | Can’t tell | Comments |
| Screening questions  (for all types) | S1. Are there clear research questions? | x |  |  | *Clearly formulated as research objectives:*   - To examine the impact of the virtual dementia tour on empathetic thinking, understanding and person care. - To examine how a virtual reality programme for health professionals and carers impact on understanding of the condition and in particular the empathetic understanding of its impact on cognitions, emotions and behaviours. |
|  | S2. Do the collected data allow to address the research questions? | x |  |  | *The data collected and analysed reflect the objective and thus answer the research question.* |
|  | *Further appraisal may not be feasible or appropriate when the answer is ‘No’ or ‘Can’t tell’ to one or both screening questions.* | | | | |
| 1. Qualitative | 1.1. Is the qualitative approach appropriate to answer the research question? |  |  |  |  |
|  | 1.2. Are the qualitative data collection methods adequate to address the research question? |  |  |  |  |
|  | 1.3. Are the findings adequately derived from the data? |  |  |  |  |
|  | 1.4. Is the interpretation of results sufficiently substantiated by data? |  |  |  |  |
|  | 1.5. Is there coherence between qualitative data sources, collection, analysis and interpretation? |  |  |  |  |
| 2. Quantitative randomized controlled trials | 2.1. Is randomization appropriately performed? |  |  |  |  |
|  | 2.2. Are the groups comparable at baseline? |  |  |  |  |
|  | 2.3. Are there complete outcome data? |  |  |  |  |
|  | 2.4. Are outcome assessors blinded to the intervention provided? |  |  |  |  |
|  | 2.5 Did the participants adhere to the assigned intervention? |  |  |  |  |
| 3. Quantitative non-randomized | 3.1. Are the participants representative of the target population? |  |  | x | *Target population of the intervention is not described, only the participants (Carers and multi-health professionals) included. It is unclear to what extent this represents the target population.* |
|  | 3.2. Are measurements appropriate regarding both the outcome and intervention (or exposure)? | x |  |  | An assessment tool was designed and psychometrically tested to measure these aspects (Slater, Hasson, Moore, Sharkey, 2020). The tool development was based on previous qualitative findings (Slater Hasson, Gillen, 2017) and further qualitative and quantitative research work into the effect of VR on participants empathetic understanding of living with dementia and reported strong psychometric properties (Slater, Hasson, Moore, Sharkey, 2020). It consisted of 15 items, rated on a 5-point Likert scale ranging from strongly disagree to strongly agree, measuring 3 constructs - empathy, understand of behaviours and role of the person in care decisions. Four additional items measured a fourth construct - experience of previous training. Additional demographic details were collected and experience/importance of knowledge of dementia. All four constructs had acceptable Cronbach alpha scores above the threshold of 0.7 and therefore considered stable. |
|  | 3.3. Are there complete outcome data? |  |  | x | *Participants included in the intervention: not clearly stated*  *Participants included in analysis: n = 223 (n = 82, 3-month follow-up)* |
|  | 3.4. Are the confounders accounted for in the design and analysis? |  | x |  | *No methods for controlling confounding factors were described.* |
|  | 3.5. During the study period, is the intervention administered (or exposure occurred) as intended? |  |  | x | *See above. It is unclear, how many participants were included in the intervention.* |
| 4. Quantitative descriptive | 4.1. Is the sampling strategy relevant to address the research question? |  |  |  |  |
|  | 4.2. Is the sample representative of the target population? |  |  |  |  |
|  | 4.3. Are the measurements appropriate? |  |  |  |  |
|  | 4.4. Is the risk of nonresponse bias low? |  |  |  |  |
|  | 4.5. Is the statistical analysis appropriate to answer the research question? |  |  |  |  |
| 5. Mixed methods | 5.1. Is there an adequate rationale for using a mixed methods design to address the research question? |  |  |  |  |
|  | 5.2. Are the different components of the study effectively integrated to answer the research question? |  |  |  |  |
|  | 5.3. Are the outputs of the integration of qualitative and quantitative components adequately interpreted? |  |  |  |  |
|  | 5.4. Are divergences and inconsistencies between quantitative and qualitative results adequately addressed? |  |  |  |  |
|  | 5.5. Do the different components of the study adhere to the quality criteria of each tradition of the methods involved? |  |  |  |  |

| [40] Sung HC, Su HF, Lee WL, Yamakawa M, Wang HM. Effects of a dementia virtual reality-based training with peer support for home care workers: A cluster randomized controlled trial. Int J Geriatr Psychiatry. 2022;37(9). | | | | | |
| --- | --- | --- | --- | --- | --- |
|  | | | | | |
| **Category of study designs** | **Methodological quality criteria** | **Responses** | | | |
|  |  | Yes | No | Can’t tell | Comments |
| Screening questions  (for all types) | S1. Are there clear research questions? | x |  |  | *Formulated as research objective:*  To evaluate the effects of a dementia VR‐based training with peer support on dementia knowledge, attitude, competence, and empathy of home care workers. |
|  | S2. Do the collected data allow to address the research questions? | x |  |  | *The data reflect the objective (testing the effect of the multicomponent intervention program) and thus answer the research question.* |
|  | *Further appraisal may not be feasible or appropriate when the answer is ‘No’ or ‘Can’t tell’ to one or both screening questions.* | | | | |
| 1. Qualitative | 1.1. Is the qualitative approach appropriate to answer the research question? |  |  |  |  |
|  | 1.2. Are the qualitative data collection methods adequate to address the research question? |  |  |  |  |
|  | 1.3. Are the findings adequately derived from the data? |  |  |  |  |
|  | 1.4. Is the interpretation of results sufficiently substantiated by data? |  |  |  |  |
|  | 1.5. Is there coherence between qualitative data sources, collection, analysis and interpretation? |  |  |  |  |
| 2. Quantitative randomized controlled trials | 2.1. Is randomization appropriately performed? | x |  |  | *The researchers describe how the randomisation plan was created, how the allocation worked and that the allocation was done concealed.* |
|  | 2.2. Are the groups comparable at baseline? | x |  |  | There were no significant differences between two groups at baseline.  N*o conclusions about an imbalance between the four groups can be drawn from Table 1.* |
|  | 2.3. Are there complete outcome data? | x |  |  | *From the available data, there is no indication of missing data.* |
|  | 2.4. Are outcome assessors blinded to the intervention provided? | x |  |  | *The data assessors were blinded to the group allocations.* |
|  | 2.5 Did the participants adhere to the assigned intervention? |  |  | x | *No statement exists on this.* |
| 3. Quantitative non-randomized | 3.1. Are the participants representative of the target population? |  |  |  |  |
|  | 3.2. Are measurements appropriate regarding both the outcome and intervention (or exposure)? |  |  |  |  |
|  | 3.3. Are there complete outcome data? |  |  |  |  |
|  | 3.4. Are the confounders accounted for in the design and analysis? |  |  |  |  |
|  | 3.5. During the study period, is the intervention administered (or exposure occurred) as intended? |  |  |  |  |
| 4. Quantitative descriptive | 4.1. Is the sampling strategy relevant to address the research question? |  |  |  |  |
|  | 4.2. Is the sample representative of the target population? |  |  |  |  |
|  | 4.3. Are the measurements appropriate? |  |  |  |  |
|  | 4.4. Is the risk of nonresponse bias low? |  |  |  |  |
|  | 4.5. Is the statistical analysis appropriate to answer the research question? |  |  |  |  |
| 5. Mixed methods | 5.1. Is there an adequate rationale for using a mixed methods design to address the research question? |  |  |  |  |
|  | 5.2. Are the different components of the study effectively integrated to answer the research question? |  |  |  |  |
|  | 5.3. Are the outputs of the integration of qualitative and quantitative components adequately interpreted? |  |  |  |  |
|  | 5.4. Are divergences and inconsistencies between quantitative and qualitative results adequately addressed? |  |  |  |  |
|  | 5.5. Do the different components of the study adhere to the quality criteria of each tradition of the methods involved? |  |  |  |  |

| [41] Torrence C, Bhanu A, Bertrand J, Dye C, Truong K, Madathil KC. Preparing future health care workers for interactions with people with dementia: A mixed methods study. Gerontol Geriatr Educ. 2022. | | | | | |
| --- | --- | --- | --- | --- | --- |
|  | | | | | |
| **Category of study designs** | **Methodological quality criteria** | **Responses** | | | |
|  |  | Yes | No | Can’t tell | Comments |
| Screening questions  (for all types) | S1. Are there clear research questions? | x |  |  | *Formulated as research objectives:*   - To assess the effectiveness of virtual reality as a delivery format for dementia tours compared to dementia tours that physically alter sensations, - To compare the effectiveness of deficit-focused dementia sensitivity training to reading strength-focused case studies, a traditional instruction method. |
|  | S2. Do the collected data allow to address the research questions? |  |  | x | *Effectiveness of an intervention should be explored with an Randomised Controlled Trial.* |
|  | *Further appraisal may not be feasible or appropriate when the answer is ‘No’ or ‘Can’t tell’ to one or both screening questions.* | | | | |
| 1. Qualitative | 1.1. Is the qualitative approach appropriate to answer the research question? | x |  |  | Qualitatively, the researchers seek to understand participants’ experiences in the three conditions, how the dementia tours in the physical and virtual formats compare to using case studies for building empathy. |
|  | 1.2. Are the qualitative data collection methods adequate to address the research question? | x |  |  | (…) interviewers used an interview reaction sheet to document the behaviors of the participants. Following completion of the posttest, using a retrospective think-aloud protocol, participants were asked to reflect on their reactions, thoughts, and behaviors while engaged in their assigned condition. |
|  | 1.3. Are the findings adequately derived from the data? | x |  |  | An inductive thematic approach guided analysis of data in the qualitative strand. This approach was chosen to limit the influence of any potential pre-conceptions that might constrain the analysis. |
|  | 1.4. Is the interpretation of results sufficiently substantiated by data? | x |  |  | *The data interpretation was underpinned by quotes that correctly describe the respective topics.* |
|  | 1.5. Is there coherence between qualitative data sources, collection, analysis and interpretation? | x |  |  | *The intended research goals are reflected in the identified themes as well as in the data interpretation.* |
| 2. Quantitative randomized controlled trials | 2.1. Is randomization appropriately performed? |  |  |  |  |
|  | 2.2. Are the groups comparable at baseline? |  |  |  |  |
|  | 2.3. Are there complete outcome data? |  |  |  |  |
|  | 2.4. Are outcome assessors blinded to the intervention provided? |  |  |  |  |
|  | 2.5 Did the participants adhere to the assigned intervention? |  |  |  |  |
| 3. Quantitative non-randomized | 3.1. Are the participants representative of the target population? |  |  | x | University students, mainly nursing and public health sciences program students were recruited.  *However, this does not provide a basis for extending the results to the entire group of university students.* |
|  | 3.2. Are measurements appropriate regarding both the outcome and intervention (or exposure)? | x |  |  | *The variables to be measured were clearly defined and therefore suitable for answering the research objective. The instruments used were validated instruments:*   - *Participant knowledge: Knowledge of Alzheimer’s Disease Scale,* - *Participant attitudes and beliefs about living with dementia: Dementia Attitudes Scale,* - *Perceived ability: Caregiving Preparedness Scale.* |
|  | 3.3. Are there complete outcome data? | x |  |  | *Participants included in the intervention: n = 41 (CS (n = 14), PDT (n = 14), and VRDT (n = 13)),*  *Participants included in analysis: n = 41 (CS (n = 14), PDT (n = 14), and VRDT (n = 13)).* |
|  | 3.4. Are the confounders accounted for in the design and analysis? |  | x |  | *No methods to control for confounding factors were applied.* |
|  | 3.5. During the study period, is the intervention administered (or exposure occurred) as intended? | x |  |  | *There are no changes in exposure status from the intended intervention.* |
| 4. Quantitative descriptive | 4.1. Is the sampling strategy relevant to address the research question? |  |  |  |  |
|  | 4.2. Is the sample representative of the target population? |  |  |  |  |
|  | 4.3. Are the measurements appropriate? |  |  |  |  |
|  | 4.4. Is the risk of nonresponse bias low? |  |  |  |  |
|  | 4.5. Is the statistical analysis appropriate to answer the research question? |  |  |  |  |
| 5. Mixed methods | 5.1. Is there an adequate rationale for using a mixed methods design to address the research question? | x |  |  | The rationale for collecting quantitative and qualitative data was that both forms would provide greater insight than the data interpreted in isolation.  *However, no rationale provided for integrating both data strands.* |
|  | 5.2. Are the different components of the study effectively integrated to answer the research question? |  |  | x | *The results were presented merged, but it is unclear how the analysis was done.* |
|  | 5.3. Are the outputs of the integration of qualitative and quantitative components adequately interpreted? |  |  | x | *Probably yes, but there is no description of the data analysis.* |
|  | 5.4. Are divergences and inconsistencies between quantitative and qualitative results adequately addressed? |  |  | x | *See above* |
|  | 5.5. Do the different components of the study adhere to the quality criteria of each tradition of the methods involved? |  |  | x | *Presence of medium quality, as partial studies are of medium to high quality.* |

| [42] Wijma EM, Veerbeek MA, Prins M, Pot AM, Willemse BM. A virtual reality intervention to improve the understanding and empathy for people with dementia in informal caregivers: results of a pilot study. Aging & Mental Health. 2017;22. | | | | | |
| --- | --- | --- | --- | --- | --- |
|  | | | | | |
| **Category of study designs** | **Methodological quality criteria** | **Responses** | | | |
|  |  | Yes | No | Can’t tell | Comments |
| Screening questions  (for all types) | S1. Are there clear research questions? | x |  |  | *Clear research questions described:*  (1) Feasibility and acceptability: what are the dropout and attendance rates? How is TDL evaluated by informal caregivers in terms of usefulness, ease of use, satisfaction  (Lund, 2001), and acceptability (Proctor et al., 2011)?  (2) Impact: what is the impact of TDL on informal caregivers with respect to their person-centred attitude, empathy (primary outcomes), and their perceived burden  of care, perceived competence, and quality of the relationship (secondary outcomes)?  (3) Are there groups of informal caregivers that especially benefit from TDL, based on their demographics? |
|  | S2. Do the collected data allow to address the research questions? | x |  |  |  |
|  | *Further appraisal may not be feasible or appropriate when the answer is ‘No’ or ‘Can’t tell’ to one or both screening questions.* | | | | |
| 1. Qualitative | 1.1. Is the qualitative approach appropriate to answer the research question? |  |  |  |  |
|  | 1.2. Are the qualitative data collection methods adequate to address the research question? |  |  |  |  |
|  | 1.3. Are the findings adequately derived from the data? |  |  |  |  |
|  | 1.4. Is the interpretation of results sufficiently substantiated by data? |  |  |  |  |
|  | 1.5. Is there coherence between qualitative data sources, collection, analysis and interpretation? |  |  |  |  |
| 2. Quantitative randomized controlled trials | 2.1. Is randomization appropriately performed? |  |  |  |  |
|  | 2.2. Are the groups comparable at baseline? |  |  |  |  |
|  | 2.3. Are there complete outcome data? |  |  |  |  |
|  | 2.4. Are outcome assessors blinded to the intervention provided? |  |  |  |  |
|  | 2.5 Did the participants adhere to the assigned intervention? |  |  |  |  |
| 3. Quantitative non-randomized | 3.1. Are the participants representative of the target population? | x |  |  | *The research objectives includes testing the feasibility and efficacy of VR for preclinical medical students. For this reason, the researchers only included medical students (second-year, pre-clinical). However, the extent to which the results are transferable to other students in this setting is questionable.* |
|  | 3.2. Are measurements appropriate regarding both the outcome and intervention (or exposure)? |  | x |  | *The variables to be measured were clearly defined and thus suitable for answering the research question: Pre-Survey: personal experiences with dementia; Post-Survey: Students thoughts about the VR experience; Both: understanding of the feelings of both caregivers and persons with dementia.*  *Some of the instruments used were self-constructed, which is to be assessed negatively. Furthermore, feasibility and efficacy could be better evaluated by a different design.* |
|  | 3.3. Are there complete outcome data? | x |  |  | *Participants of the intervention: n = 150,*  *Participants included in analysis: n = 150 (pre-survey: n = 149; post-survey: n = 150).* |
|  | 3.4. Are the confounders accounted for in the design and analysis? |  | x |  | *No methods for controlling confounding factors were described.* |
|  | 3.5. During the study period, is the intervention administered (or exposure occurred) as intended? | x |  |  | *There are no changes in exposure status from the intended intervention.* |
| 4. Quantitative descriptive | 4.1. Is the sampling strategy relevant to address the research question? |  |  |  |  |
|  | 4.2. Is the sample representative of the target population? |  |  |  |  |
|  | 4.3. Are the measurements appropriate? |  |  |  |  |
|  | 4.4. Is the risk of nonresponse bias low? |  |  |  |  |
|  | 4.5. Is the statistical analysis appropriate to answer the research question? |  |  |  |  |
| 5. Mixed methods | 5.1. Is there an adequate rationale for using a mixed methods design to address the research question? |  |  |  |  |
|  | 5.2. Are the different components of the study effectively integrated to answer the research question? |  |  |  |  |
|  | 5.3. Are the outputs of the integration of qualitative and quantitative components adequately interpreted? |  |  |  |  |
|  | 5.4. Are divergences and inconsistencies between quantitative and qualitative results adequately addressed? |  |  |  |  |
|  | 5.5. Do the different components of the study adhere to the quality criteria of each tradition of the methods involved? |  |  |  |  |

## Chapter D: Tabular presentation of publication trend by modality of intervention*

| **Modality of intervention** | **Year of publication/ reporting**** | **Number of publications/ year** | **References of included intervention programmes***** |
| --- | --- | --- | --- |
| Passive interventions | 2015 | 1 | [8] |
|  | 2016 | 1 | [3] |
|  | 2019 | 1 | [34] |
| Interactive interventions | 2015 | 1 | [28] |
|  | 2017 | 2 | [7, 37] |
|  | 2018 | 1 | [17] |
|  | 2019 | 5 | [12, 14, 19, 35, 36] |
|  | 2020 | 2 | [13, 32] |
|  | 2021 | 3 | [6, 15, 38] |
|  | 2022 | 2 | [24, 41] |
| Immersive interventions | 2015 | 1 | [16] |
|  | 2016 | 1 | [1] |
|  | 2018 | 1 | [20] |
|  | 2019 | 2 | [5, 29] |
|  | 2021 | 1 | [39] |
|  | 2022 | 2 | [4, 41] |
| Multicomponent interventions | 2010 | 1 | [25] |
|  | 2016 | 2 | [2, 27] |
|  | 2017 | 2 | [26, 42] |
|  | 2018 | 3 | [10, 11, 22] |
|  | 2019 | 3 | [9, 30, 31] |
|  | 2020 | 4 | [13, 18, 23, 33] |
|  | 2021 | 1 | [21] |
|  | 2022 | 1 | [40] |

*Note(s):* * Study protocols and registrations were not included in the analysis; ** Date of the first published official version (usually online); preprint and preproof versions were not taken into account; *** Publications describing multiple intervention programmes (across modalities) are listed per intervention programme.

## Chapter E: Graphical presentation of publication trend by modality of intervention^*,***^

*Note(s):* * Study protocols and registrations were not included in the analysis; ** Date of the first published official version (usually online); preprint and preproof versions were not taken into account; ***Publications describing multiple intervention programs (across modalities) are listed per intervention programme.

## Chapter F: Barriers and Facilitators per intervention type*

| **Intervention type** | **Barriers** | **Facilitators** |
| --- | --- | --- |
| Film interventions | - Caution required in selecting dissemination channels - Preference for initial scenes over final scenes - Time conflicts with professional work hindrance | - Supportive roles (e.g., managers or dementia officers) - Organisational involvement - Providing multiple access points to the intervention - Implementing the intervention over an extended period - Adopting a first-person perspective in the development |
| Theatre interventions | *None reported* | *None reported* |
| Physical simulation interventions | - High resource intensity - Time-intensive - Time conflicts with professional work hindrance - Stress caution during training | - Exchanging experiences and debriefing sessions |
| Role-play interventions | - High resource intensity - Intensive supervision of amateur actors - Importance of joint reflection with participants lacking experience with people with dementia | - Constant availability of students (as actors) - Provision of briefings - Clear role descriptions - Suitability as an alternative to clinical practice |
| Game interventions | *None reported* | *None reported* |
| Virtual Reality interventions | - Challenges with VR goggles for participants with visual deficits - Motion sickness - Prohibitive initial cost of equipment and software | - Previous VR experience |
| Mixed Reality interventions | - High cost | - Sharing experiences (e.g., through group meetings or follow-up sessions) - Condition progression |
| Combination of self-experience practices | - Tension between information and entertainment in training intervention - High resource intensity - Caution of emotional reactions | - Accommodation of staff availability - Availability of resources - Collaboration with local healthcare providers - Strong debriefing skills and experience of facilitator - Time to practice skills in safe environment |
| Combination of self-experience practices with other learning methods | - Caution of anxiety and stress - Dependency and incurring costs when involving external actors - Emphasis on early-stage Alzheimer’s neglecting other dementia forms and stages | - Mutual support among participants - Face-to-face contact with people with dementia - Training delivered either face-to-face or through a combination of online and face-to-face programmes - Consecutive days of training - Comprehensive and coordinated content integrated into curricula - Team-based approaches - Reflection and discussion sessions - Affordability - Ease of access - Short conversations or reflections after watching a film |

*Note(s):* * Study protocols and registrations were not included in the analysis

## Chapter G: Intended and reported learning outcomes of the included reports (categorised by modality and type of intervention)

| **Modality** | **Type** | **Learning outcomes** [43, 44] | | | | **Reference of included report **,***** |
| --- | --- | --- | --- | --- | --- | --- |
|  |  | **Intended/Reported*** | | | |  |
|  |  | Reaction | Learning | Behavior | Results |  |
| Passive interventions | Film interventions | x/x | x/x | x/x | x/x | [3] |
|  |  | x/x | x/x | -/- | -/- | [34] |
|  | Theatre interventions | -/- | x/x | x/x | -/- | [8] |
| Interactive interventions | Physical simulation interventions | x/x | x/x | x/x | -/- | [12] |
|  |  | x/x | x/x | x/x | -/- | [14] |
|  |  | -/x | x/x | x/x | -/- | [13] |
|  |  | x/x | x/x | -/- | -/- | [19] |
|  |  | -/- | x/x | -/- | -/- | [7] |
|  |  | x/x | x/x | -/- | -/- | [41] |
|  |  | -/x | x/x | -/- | -/- | [6] |
|  |  | x/x | x/x | -/- | -/x | [15] |
|  |  | x/x | x/x | -/- | -/- | [24] |
|  |  | -/x | x/x | -/- | -/- | [32] |
|  |  | x/x | x/x | -/x | -/- | [35] |
|  |  | x/x | x/x | x/x | -/- | [37] |
|  |  | x/x | x/x | -/x | -/- | [36] |
|  |  | x/x | x/x | x/- | -/- | [38] |
|  | Role-play interventions | -/- | x/x | -/- | -/- | [17] |
|  |  | -/- | x/x | -/- | -/- | [28] |
| Immersive interventions | Game interventions | -/- | x/x | x/x | -/- | [29] |
|  | Virtual Reality interventions | -/x | -/x | x/x | -/- | [5] |
|  |  | x/x | x/x | -/- | -/- | [4] |
|  |  | x/x | x/x | -/- | -/- | [39] |
|  |  | x/x | x/x | -/- | -/- | [1] |
|  |  | x/x | x/x | -/- | -/- | [41] |
|  | Mixed Reality interventions | x/x | x/x | -/x | -/- | [16] |
|  |  | x/x | x/x | -/- | -/- | [20] |
| Multicomponent interventions | Combination of self-experience practices | x/x | x/x | x/x | -/- | [2] |
|  |  | x/x | x/x | x/x | x/- | [25] |
|  |  | -/x | x/x | -/- | -/- | [26] |
|  |  | x/x | x/x | -/- | -/- | [33] |
|  |  | x/x | x/x | -/- | -/- | [27] |
|  | Combination of self-experience practices with other learning methods | x/x | x/x | x/x | x/- | [9] |
|  |  | x/x | x/x | x/x | x/x | [18] |
|  |  | -/- | x/x | -/- | -/- | [10] |
|  |  | -/x | x/x | -/- | -/- | [11] |
|  |  | x/x | x/x | x/- | -/- | [21] |
|  |  | x/x | x/x | -/- | -/- | [40] |
|  |  | x/x | x/x | -/- | -/- | [22] |
|  |  | x/x | x/x | -/- | -/- | [23] |
|  |  | x/x | x/x | -/- | -/- | [30] |
|  |  | x/x | x/x | -/- | -/- | [31] |
|  |  | x/x | x/x | -/- | -/- | [42] |
|  |  | -/x | x/x | x/x | -/- | [13] |

*Note(s):* * x = reported in the publication, - = not reported in the publication; ** Study protocols and registrations were not included in the analysis; *** Publications describing multiple intervention programmes (across modalities) are listed per intervention programme

# References

1. Adefila A, Graham S, Clouder L, Bluteau P, Ball S. myShoes – the future of experiential dementia training? Journal of Mental Health Training, Education & Practice. 2016;11(2):91-101. doi: 10.1108/JMHTEP-10-2015-0048. PubMed PMID: 115235953. Language: English. Entry Date: 20180117. Revision Date: 20190204. Publication Type: Article.

2. Argyle E, Schneider J. Research based theatre in dementia knowledge transfer: views from the front line. Journal of Mental Health Training, Education & Practice. 2016;11(2):102-11. doi: 10.1108/JMHTEP-09-2015-0047. PubMed PMID: 115235952. Language: English. Entry Date: 20180117. Revision Date: 20180118. Publication Type: Article.

3. Baillie L, Sills E, Thomas N. Educating a health service workforce about dementia: a qualitative study. Quality in Ageing & Older Adults. 2016;17(2):119-30. doi: 10.1108/QAOA-11-2015-0051. PubMed PMID: 115728176. Language: English. Entry Date: 20180430. Revision Date: 20180430. Publication Type: Article.

4. Bard JT, Chung HK, Shaia JK, Wellman LL, Elzie CA. Increased medical student understanding of dementia through virtual embodiment. Gerontology & Geriatrics Education. 2022;44(2):211-22. Epub 20220422. doi: 10.1080/02701960.2022.2067850. PubMed PMID: 35451921.

5. Board M, Murphy J, Mitchell R, Phipps L, Fossey I. The Lived Experience of Dementia. A new resource for the health and social care workforce. 2019.

6. Campbell D, Lugger S, Sigler GS, Turkelson C. Increasing awareness, sensitivity, and empathy for Alzheimer's dementia patients using simulation. Nurse Education Today. 2021;98:e104764. doi: 10.1016/j.nedt.2021.104764. PubMed PMID: 148733179. Language: English. Entry Date: 20210222. Revision Date: 20210222. Publication Type: Article.

7. de Abreu ID, Hinojosa-Lindsey M, Asghar-Ali AA. A Simulation Exercise to Raise Learners' Awareness of the Physical and Cognitive Changes in Older Adults. Academic Psychiatry. 2017;41(5):684-7. doi: 10.1007/s40596-017-0775-4. PubMed PMID: WOS:000413546500025.

8. Dupuis SL, Mitchell GJ, Jonas-Simpson CM, Whyte CP, Gillies JL, Carson JD. Igniting Transformative Change in Dementia Care Through Research-based Drama. Gerontologist. 2015;56(6):1042-52. doi: 10.1093/geront/gnv062. PubMed PMID: 119547578. Language: English. Entry Date: 20161123. Revision Date: 20171201. Publication Type: Article.

9. Garrod L, Fossey J, Henshall C, Williamson S, Coates A, Green H. Evaluating dementia training for healthcare staff. The Journal of Mental Health Training, Education and Practice. 2019;14(4):277-88. doi: 10.1108/jmhtep-10-2018-0062.

10. Gilmartin-Thomas JFM, McNeil J, Powell A, Malone DT, Wolfe R, Larson IC, et al. Impact of a Virtual Dementia Experience on Medical and Pharmacy Students' Knowledge and Attitudes Toward People with Dementia: A Controlled Study. J Alzheimers Dis. 2018;62(2):867-76. doi: 10.3233/JAD-170982. PubMed PMID: 29480198.

11. Gilmartin-Thomas JFM, McNeil J, Powell A, Malone DT, Larson IC, O'Reilly CL, et al. Qualitative evaluation of how a virtual dementia experience impacts medical and pharmacy students' self-reported knowledge and attitudes towards people with dementia. Dementia (14713012). 2018;19(2):205-20. doi: 10.1177/1471301218770270. PubMed PMID: 141167977. Language: English. Entry Date: 20200120. Revision Date: 20200120. Publication Type: Article.

12. Han A, Brown D. Experiences of Caregivers in a Dementia Simulation Program. Journal of Social Service Research. 2019. doi: 10.1080/01488376.2018.1524814. PubMed PMID: WOS:000507244500006.

13. Han A, Kim TH, Hong H. A factorial randomized controlled trial to examine separate and combined effects of a simulation-based empathy enhancement program and a lecture-based education program on family caregivers of people with dementia. Aging & Mental Health. 2020. doi: 10.1080/13607863.2020.1768214. PubMed PMID: 152609887. Language: English. Entry Date: 20210929. Revision Date: 20210929. Publication Type: Article.

14. Han A, Kim TH, Hong H. Experiences of caregivers of people with dementia in a Korean dementia simulation program. Dementia. 2019;19(7):2415-29. doi: 10.1177/1471301218823453. PubMed PMID: 148979304. Language: English. Entry Date: 20210305. Revision Date: 20210305. Publication Type: Article.

15. Harrington CC, Neil JA, Hardin SR, Roberson DW. Is Perception Reality? Using Person-in-Context Simulation to Promote Empathic Understanding of Dementia Among Nurse Practitioner Students. Nurs Educ Perspect. 2021;42(6):377-9. doi: 10.1097/01.NEP.0000000000000780. PubMed PMID: 33555835.

16. Hattink BJJ, Meiland FJM, Campman CAM, Rietsema J, Sitskoorn M, Dröes R-M. Zelf dementie ervaren: Ontwikkeling en evaluatie van de Into D’mentia simulator [Experiencing dementia: evaluation of Into D'mentia]. Tijdschr Gerontol Geriatr. 2015;46(5):262-81. doi: 10.1007/s12439-015-0130-8. PubMed PMID: 25968906.

17. Haugland VL, Reime MH. Scenario-based simulation training as a method to increase nursing students' competence in demanding situations in dementia care. A mixed method study. Nurse Education in Practice. 2018;33:164-71. doi: 10.1016/j.nepr.2018.08.008. PubMed PMID: 132941334. Language: English. Entry Date: 20181114. Revision Date: 20181114. Publication Type: Article.

18. Heward M, Board M, Spriggs A, Emerson L, Murphy J. Impact of 'DEALTS2' education intervention on trainer dementia knowledge and confidence to utilise innovative training approaches: A national pre-test - post-test survey. Nurse Educ Today. 2020;97:104694. Epub 20201207. doi: 10.1016/j.nedt.2020.104694. PubMed PMID: 33321300.

19. Jeong H, Kim TH, Han AR. Effects of Korean dementia simulation program for caregivers of the elderly with dementia pilot study. European Neuropsychopharmacology. 2019;29:S130-S1. doi: 10.1016/j.euroneuro.2019.09.215.

20. Jütten LH, Mark RE, Sitskoorn MM. Can the Mixed Virtual Reality Simulator Into D'mentia Enhance Empathy and Understanding and Decrease Burden in Informal Dementia Caregivers? Dementia and Geriatric Cognitive Disorders Extra. 2018;8(3):453-66. doi: 10.1159/000494660. PubMed PMID: WOS:000465207700013.

21. Kimzey M, Mastel-Smith B. Impact of dementia simulation on nursing students: When empathy breeds awareness. Teaching & Learning in Nursing. 2021. doi: 10.1016/j.teln.2021.11.010. PubMed PMID: 155726447. Language: English. Entry Date: 20220323. Revision Date: 20220323. Publication Type: Article.

22. Kimzey M, Mastel-Smith B, Seale A. Effects of Dementia-Specific Education for Nursing Students. Nurse Educator. 2018;44(6):338-41. doi: 10.1097/NNE.0000000000000623. PubMed PMID: 140958833. Language: English. Entry Date: 20200103. Revision Date: 20200106. Publication Type: Article.

23. Kimzey M, Patterson J, Mastel-Smith B. Effects of Simulation on Nursing Students' Dementia Knowledge and Empathy: A Mixed Method Study. Issues in Mental Health Nursing. 2020. doi: 10.1080/01612840.2020.1797252. PubMed PMID: 149091834. Language: English. Entry Date: 20210311. Revision Date: 20220525. Publication Type: Article.

24. Kobiske KR, Deprey SM. A Dementia Simulation as a Teaching Strategy for Nursing and Physical Therapy Students: A Qualitative Study. Clinical Simulation in Nursing. 2022;67:11-7. doi: 10.1016/j.ecns.2022.03.007. PubMed PMID: 156983697. Language: English. Entry Date: 20220602. Revision Date: 20220602. Publication Type: Article.

25. Kontos PC, Mitchell GJ, Mistry B, Ballon B. Using drama to improve person-centred dementia care. Int J Older People Nurs. 2010;5(2):159-68. doi: 10.1111/j.1748-3743.2010.00221.x. PubMed PMID: 20925717; PubMed Central PMCID: PMCPMC3786173.

26. Leah V, Combes J, McMillan M, Russell L, McCune K. Experiences of using simulation in dementia education. Nurs Older People. 2017;29(8):27-34. doi: 10.7748/nop.2017.e901. PubMed PMID: 29124914.

27. Lorio AK, Gore JB, Warthen L, Housley SN, Burgess EO. Teaching dementia care to physical therapy doctoral students: A multimodal experiential learning approach. Gerontol Geriatr Educ. 2016. Epub 20160217. doi: 10.1080/02701960.2015.1115979. PubMed PMID: 26885693.

28. Maharaj T. Live Model Simulation: Improving Nursing Students' Attitudes and Knowledge of Alzheimer's Disease: The University of Texas at Tyler; 2015.

29. Maskeliūnas R, Damaševičius R, Lethin C, Paulauskas A, Esposito A, Catena M, et al. Serious Game iDO: Towards Better Education in Dementia Care. Information. 2019;10(11). doi: 10.3390/info10110355.

30. Mastel-Smith B, Kimzey M, Zhaomin H. Dementia Care Education for Nursing Students. Journal of Nursing Education. 2019;58(3):136-43. doi: 10.3928/01484834-20190221-03. PubMed PMID: 135096606. Language: English. Entry Date: 20190308. Revision Date: 20190315. Publication Type: Article.

31. Mastel-Smith B, Kimzey M, Garner J, Shoair OA, Stocks E, Wallace T. Dementia care boot camp: interprofessional education for healthcare students. Journal of Interprofessional Care. 2019. doi: 10.1080/13561820.2019.1696287. PubMed PMID: 147339147. Language: English. Entry Date: 20201206. Revision Date: 20220125. Publication Type: Article.

32. Meyer K, James D, Amezaga B, White C. Simulation learning to train healthcare students in person-centered dementia care. Gerontology & Geriatrics Education. 2020. doi: 10.1080/02701960.2020.1838503. PubMed PMID: 157383476. Language: English. Entry Date: 20220623. Revision Date: 20220623. Publication Type: Article.

33. Peng XY, Wu LF, Xie XS, Dai MJ, Wang DH. Impact of Virtual Dementia Tour on empathy level of nursing students: A quasi-experimental study. International Journal of Nursing Sciences. 2020;7(3):258-61. doi: 10.1016/j.ijnss.2020.06.010. PubMed PMID: WOS:000558676500002.

34. Prins M, Veerbeek M, Willemse BM, Pot AM. Use and impact of the Alzheimer Experience: a free online media production to raise public awareness and enhance knowledge and understanding of dementia. Aging & Mental Health. 2019;24(6):985-92. doi: 10.1080/13607863.2019.1579781. PubMed PMID: WOS:000536716600017.

35. Sharkey F, Moore K, Slater P, Hassan F. The Impact of a Virtual Reality Training Programme on Health Professional Knowledge, Understanding and Empathy in Relation to Dementia. 2019.

36. Slater P, Hasson F, Gillen P, Gallen A, Parlour R. Virtual simulation training: Imaged experience of dementia. International Journal of Older People Nursing. 2019;14(3):e12243. doi: 10.1111/opn.12243. PubMed PMID: 138311881. Language: English. Entry Date: 20190831. Revision Date: 20200831. Publication Type: Article.

37. Slater P, Hasson F, Gillen P. A research evaluation of an interactive training experience: The virtual Dementia Tour (VDT). Ulster University, 2017.

38. Slater P, Hasson F, Moore K, Sharkey F. Simulated Based Dementia Training: Impact on Empathic Understanding and Behaviour Among Professionals and Carers. Clinical Simulation in Nursing. 2021;55:43-51. doi: 10.1016/j.ecns.2021.04.004. PubMed PMID: 150445186. Language: English. Entry Date: 20210608. Revision Date: 20210608. Publication Type: Article.

39. Stargatt J, Bhar S, Petrovich T, Bhowmik J, Sykes D, Burns K. The Effects of Virtual Reality-Based Education on Empathy and Understanding of the Physical Environment for Dementia Care Workers in Australia: A Controlled Study. J Alzheimers Dis. 2021;84(3):1247-57. doi: 10.3233/jad-210723. PubMed PMID: 34633323.

40. Sung HC, Su HF, Lee WL, Yamakawa M, Wang HM. Effects of a dementia virtual reality-based training with peer support for home care workers: A cluster randomized controlled trial. Int J Geriatr Psychiatry. 2022;37(9). doi: 10.1002/gps.5799. PubMed PMID: 35996760.

41. Torrence C, Bhanu A, Bertrand J, Dye C, Truong K, Madathil KC. Preparing future health care workers for interactions with people with dementia: A mixed methods study. Gerontol Geriatr Educ. 2022. doi: 10.1080/02701960.2022.2042805. PubMed PMID: 35393916.

42. Wijma EM, Veerbeek MA, Prins M, Pot AM, Willemse BM. A virtual reality intervention to improve the understanding and empathy for people with dementia in informal caregivers: results of a pilot study. Aging & Mental Health. 2017;22. doi: 10.1080/13607863.2017.1348470. PubMed PMID: 132729047. Language: English. Entry Date: 20181103. Revision Date: 20200224. Publication Type: Article.

43. Kirkpatrick DL. Techniques for evaluating training programmes. Training and development journal. 1979:178-92.

44. Kirkpatrick JD, Kirkpatrick WK. Kirkpatrick's Four Level of Training Evaluation: Association for Talent Development; 2016. 256 p.
